# Synthesis of Bioconjugation Reagents for Use in Covalent Cross-Linking of Proteins by Azide-Alkyne Cycloaddition

**DOI:** 10.3390/molecules30234623

**Published:** 2025-12-02

**Authors:** Nadja Suhorepec, Luka Ciber, Uroš Grošelj, Nejc Petek, Bogdan Štefane, Marko Novinec, Jurij Svete

**Affiliations:** Faculty of Chemistry and Chemical Technology, University of Ljubljana, Večna pot 113, 1000 Ljubljana, Slovenia

**Keywords:** protein cross-linking, azide-alkyne cycloaddition, fluorescein dyes, amidation, benzotriazolides

## Abstract

A series of azide- and cyclooctyne-functionalized *N*-hydroxysuccinimidyl esters (NHS esters) and benzotriazolides were prepared and used as N-acylation reagents to obtain azide-(**BSA-1**) and cyclooctyne-functionalized bovine serum albumin proteins (**BSA-2**), fluorescein derivatives **5** and **6**, and homobifunctional linkers **3** and **4**. Strain-promoted azide-alkyne cycloaddition (SPAAC) and copper-catalyzed azide-alkyne cycloaddition (CuAAC) of azide-functionalized fluorescent probe **5** and alkyne-functionalized fluorescent probe **6** with complementary functionalized proteins **BSA-2** and **BSA-1** yielded fluorescent cycloadducts **BSA-2-5** and **BSA-1-6**. These cycloadducts were used to determine the loading of **BSA-1** and **BSA-2** with the respective azido and cyclooctyne groups based on their molar absorbances and fluorescence intensities. Dimerization through covalent cross-linking of **BSA** was then performed by SPAAC between azide-functionalized **BSA-1** and cyclooctyne-functionalized **BSA-2**, and by treating **BSA-1** and **BSA-2** with 0.5 equiv. of complementary bis-cyclooctyne linker **4** and bis-azide linker **3**. Although the formation of covalent dimers **BSA-1-2-BSA**, **BSA-1-6-1-BSA**, and **BSA-2-5-2-BSA** was detected by SDS-PAGE analysis, this was a minor process, and most of the functionalized **BSA** did not form covalent dimers.

## 1. Introduction

Chemical modification of proteins is a key technique in bioconjugation that enables various synthetic molecules to be covalently attached to proteins. The resulting derivatized proteins serve as tools for investigating biological processes. An important modification method is cross-linking proteins by forming strong covalent bonds between specific amino acid residues within or between protein molecules. Cross-linking plays a significant role in stabilizing protein structures, maintaining tissue integrity and strength, altering protein functions, inducing pathological conditions, and facilitating interactions between proteins and between proteins and other molecules. Therefore, understanding and manipulating protein cross-linking processes is essential for elucidating biological mechanisms, developing therapeutic interventions, and engineering biomaterials with tailored properties [1,2,3].

Since its definition in 2001 [4], “click” chemistry has become an important method in modern synthetic organic chemistry encompassing various highly efficient reactions, such as nucleophilic opening of spring-loaded rings, non-aldol carbonyl chemistry, additions to C−C multiple bonds and cycloadditions. A typical “click” reaction should also be bioorthogonal, meaning it should not interact with biological systems. Among the various “click” reactions, the Cu-catalyzed azide-alkyne cycloaddition (CuAAC) [5,6] emerged as the first and is now the best-known example of a “click” reaction [7,8,9,10]. In biological systems, however, CuAAC is often not truly bioorthogonal due to possible interactions of copper ions with biomolecules [1]. This limitation was soon addressed by the introduction of a catalyst-free strain-promoted azide-alkyne cycloaddition (SPAAC) in 2004 [11,12,13,14]. Since then, CuAAC and SPAAC have found widespread application in connecting various types of small molecules and macromolecular entities and are now standard ligation tools in combinatorial synthesis, [15,16,17] bioconjugation [18,19,20,21], and materials science [22,23].

**Figure 1:**
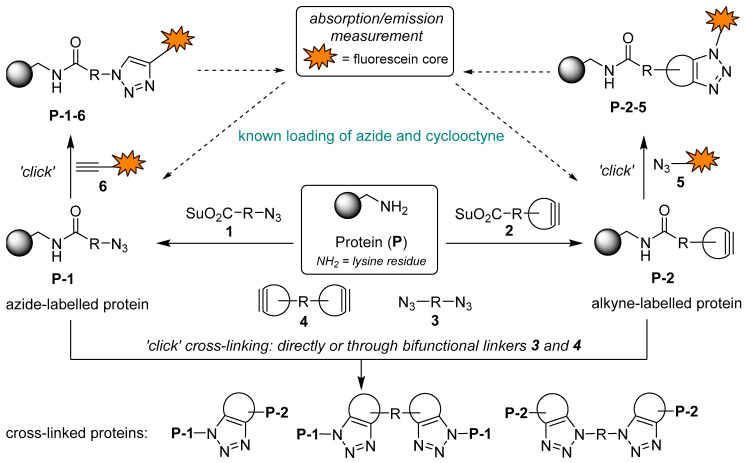
The planned method for studying covalent binding of native proteins.

Recently, we focused on applied studies aimed at labeling and covalently cross-linking native proteins using novel synthetic organic molecules as bioconjugation reagents [24,25]. The concept of linking native proteins is presented in Figure 1. Following known protocols [1,2,3], the lysine residues of a protein would be treated in parallel with excess N-acylation reagents **1** and **2** to obtain the azide-(**P-1**) and cycloalkyne-functionalized proteins (**P-2**). Linking these two would be achieved by SPAAC, either directly or via bifunctional spacers **3** and **4**. The loading of each functional group would be determined spectrophotometrically from absorbances and/or emission intensities of fluorescently labeled proteins **P-1-6** and **P-2-5**, obtained by SPAAC or CuAAC reactions between azide- and alkyne-functionalized proteins **P-1** and **P-2** and their complementary functionalized fluorescein derivatives **5** and **6**. With optimal reaction conditions to achieve the desired functional group loading FG/P ~1, the proteins **P-1** and **P-2** could then be used in “click” cross-linking experiments (Figure 1).

Contrary to our expectations, synthesizing both novel and known reagents proved challenging despite the extensive literature coverage on the subject. Therefore, we report the results of the first part of our ongoing study, the synthesis of a series of azide- and alkyne-functionalized bioconjugation reagents, fluorescent probes, and bifunctional linkers, as well as their application in the covalent cross-linking of bovine serum albumin (**BSA**).

## 2. Results and Discussion

### 2.1. Bioconjugation Reagents and Linkers Used in This Study

**Figure 2:**
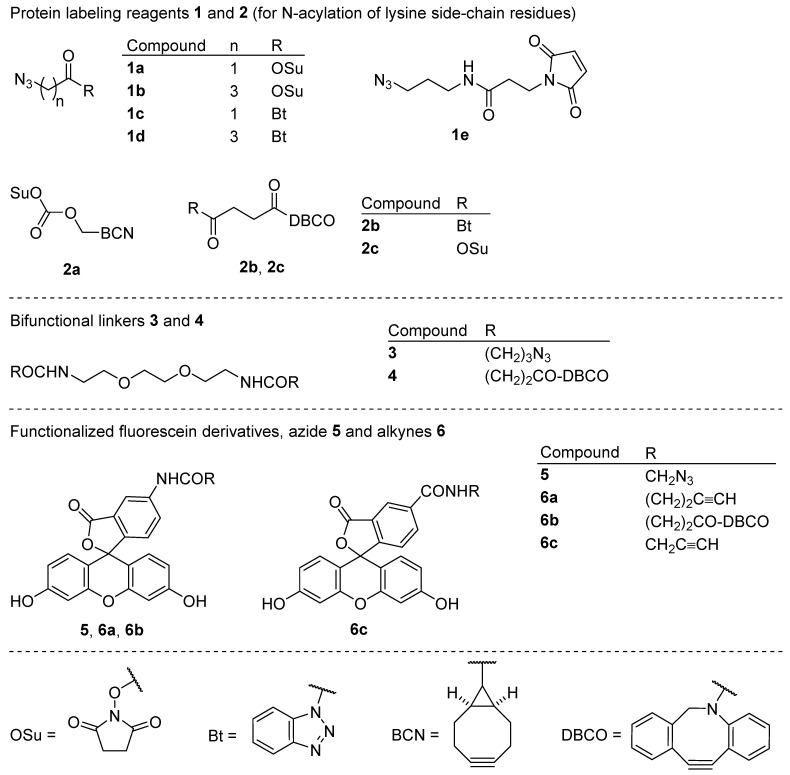
Azide- and alkyne-functionalized bioconjugation reagents **1a**–**e** and **2a**–**c**, homobifunctional linkers **3** and **4**, and fluorescein derivatives **5** and **6a**–**c** selected for use in this study.

To minimize troubleshooting events and optimize time, we kept all aspects of this study as simple as possible. Accordingly, SPAAC and CuAAC were selected as the most suitable “click” reactions to begin with. For the same reason, fluorescein derivatives were chosen as fluorescent probes, due to their availability and well-known optical properties. This approach required the use of various readily available azide-, cyclooctyne-, and alkyne-functionalized reagents to achieve controlled covalent linking of two proteins. To avoid excessive cross-linking and protein oligomerization, each protein molecule should be tagged with only one binding functional group. Bioconjugation reagents for functionalizing proteins with the azido group **1a** [26], **1b** [27], **1c** [28], **1d**, and **1e** [29] and with the cyclooctyne group **2a** [30,31], **2b**, and **2c** [32], homobifunctional “click” linkers **3** and **4**, and “click” fluorescent probes **5** [33,34], **6a** [35], **6b**, and **6c** [36,37], which were selected for use in this study, are shown in Figure 2. NHS esters **1a**, **1b**, **2a**, and **2c** were chosen as reagents for covalent binding to **BSA** by N-acylation of accessible primary amino groups in the side chains of lysine residues. Additionally, the maleimide coupling reagent **1e** was selected, as at pH~7 it can react with both lysine and cysteine residues of **BSA** through different binding modes (condensation and 1,4-addition) [1]. The 6-Aminofluoresceine derivatives **5** and **6a** were chosen as fluorescent probes for determining functional group loading (FG/BSA) in azide-(**BSA-1**) and alkyne-functionalized proteins (**BSA-2**). Compounds **1c**, **1d**, **2b**, **3**, **4**, and **6b** are novel and have not been previously reported in the literature. Bioconjugation reagents **1a**, **1b**, **1e**, **2a**, **2c**, and fluorescent probe **6c** are commercially available. Other reagents and linkers were synthesized from commercial precursors, and their syntheses are described in the following sections of this article.

### 2.2. Synthesis of Azide- and Cyclooctyne-Functionalized Conjugation Reagents ***1*** and ***2*** for N-Acylation of Lysine Side-Chain Residues

**Scheme 1:**
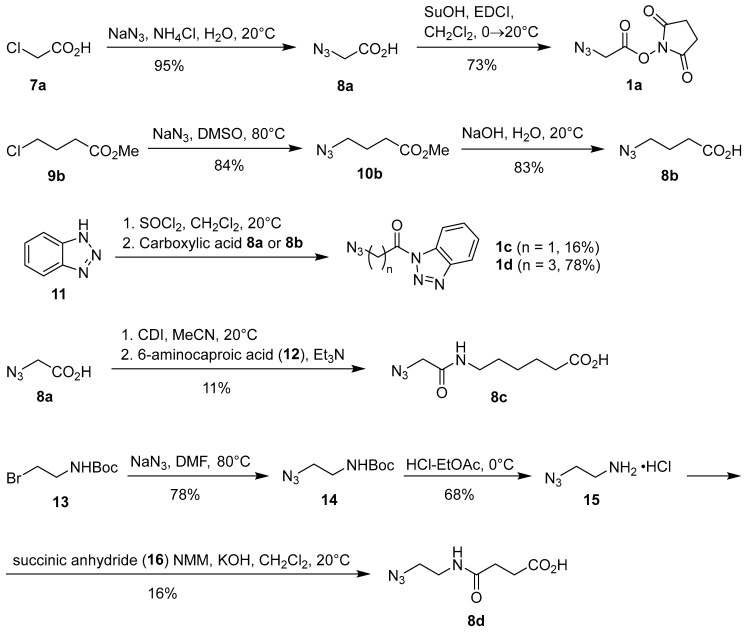
Synthesis of azido acids **8a**–**d** and azide-functionalized acylation reagents **1a**,**c**,**d**.

Due to the high cost (typically >1000 €/g) of commercial reagents for covalent binding to the lysine residues of proteins, we decided to prepare at least some reagents following the literature procedures. The synthesis of azide-functionalized reagents **1**, **8**, **10**, **13**, and **14** is shown in Scheme 1. First, chloroacetic acid (**7a**) was treated with sodium azide in aqueous ammonium chloride at room temperature for three days, followed by acidification and extraction workup to give azidoacetic acid (**8a**) in almost quantitative yield [38]. Treatment of acid **8a** with *N*-hydroxysuccinimide and *N*-ethyl-*N*′-[3-(dimethylamino)propyl]carbodiimide hydrochloride (EDCI) followed by extraction workup, then gave the desired *N*-succinimidyl azidoacetate (**1a**) in 73% yield [26]. Next, we synthesized 4-azidobutanoic acid (**8b**) in two steps from methyl 4-chlorobutanoate (**9b**) by treatment with NaN_3_ in DMSO at 80 °C and subsequent hydrolysis of methyl 4-azidobutanoate (**10b**) in 70% yield over two steps [39]. Since NHS esters are prone to hydrolysis with a typical half-life of 4–5 h at 0 °C and pH 7 [1,40], only freshly prepared stock solutions of NHS esters should be used in bioconjugation to minimize undesired hydrolysis. We also prepared more robust and stable benzotriazolide analogs **1c** and **1d** in 16% and 78% yield, respectively, by acylation of 1*H*-benzo[*d*][1,2,3]triazole (**11**) with azidoacetic acid (**8a**) and 4-azidobutyric acid (**8b**), following the general literature procedure for N-acylation of 1*H*-benzo[*d*][1,2,3]triazole (**11**) [41]. We also prepared the ‘extended’ azido acids **8c** and **8d**. Activation of azidoacetic acid (**8a**) with CDI, followed by treatment with a slight excess of 6-aminocaproic acid (**12**), gave the corresponding carboxamide **8c** in 11% yield. Reaction of 2-(*tert*-butoxycarbonylamino)-1-bromoethane (**13**) with sodium azide in DMF gave the corresponding azide **14** [42,43], which was deprotected with HCl–EtOAc to furnish 2-azidoethylamine hydrochloride (**15**) [44,45]. Subsequent treatment of **15** with succinic anhydride (**16**) in the presence of excess base gave 4-[(2-azidoethyl)amino]-4-oxobutanoic acid (**8d**) in 16% yield (Scheme 1) [46].

**Scheme 2:**
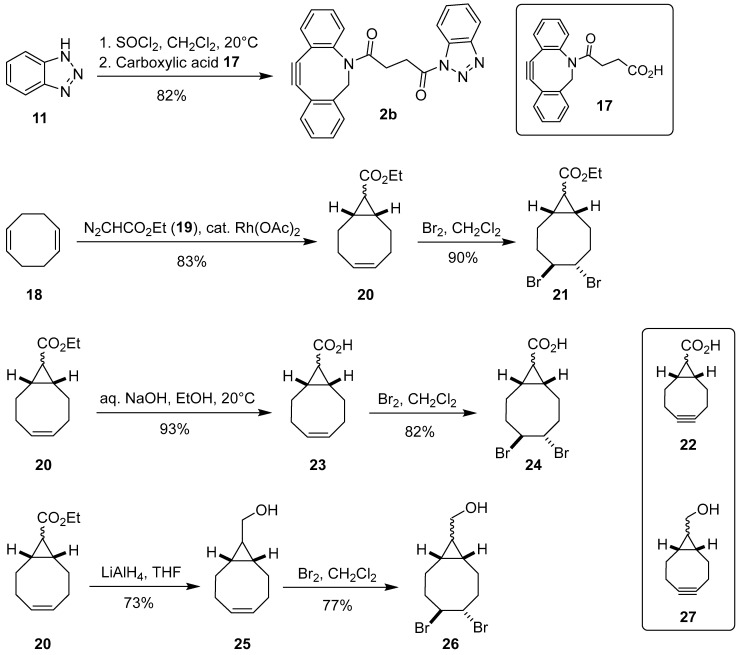
Synthesis of cyclooctyne-functionalized acylation reagent **2b** and synthesis of bicyclo[6.1.0]non-4-yne derivatives **20**, **21**, and **23**–**26**.

The synthesis of cyclooctyne-functionalized acylation reagent **2b**, compounds **20, 21**, **23**–**26**, and the attempted syntheses of (1R*,8S*)-bicyclo [6.1.0]non-4-yne-9-carboxylic acid (**22**) and (1R*,8S*)-bicyclo[6.1.0]non-4-yn-9-yl)methanol (**27**) are shown in Scheme 2. Acylation of benzotriazole **11** with 4-(11,12-didehydrodibenzo[*b*,*f*]azocin-5(6*H*)-yl)-4-oxobutanoic acid (DBCO-acid) (**17**), following the general literature procedure for the synthesis of *N*-acylbenzotriazoles [41] gave *N*-[4-(11,12-didehydrodibenzo[*b*,*f*]azocin-5(6*H*)-yl)-4-oxobutanyl]-1*H*-benzo[*d*][1,2,3]triazole (**2b**) in 82% yield. Next, we turned our attention to the preparation of *exo/endo*-(1R*,8S*)-bicyclo[6.1.0]non-4-yne-9-carboxylic acid (**22**). Rh-catalyzed reaction of ethyl diazoacetate (**19**) with a large excess of 1*Z*,5*Z*-1,5-cyclooctadiene (**18**) gave ethyl (1R*,8S*)-bicyclo[6.1.0]non-4-yne-9-carboxylate (**20**) as an *endo/exo*-mixture of isomers in 83% yield [47]. Bromination of **20** in dichloromethane gave the expected *trans*-adduct **21** [48] in 90% yield; however, attempts to obtain the target cyclooctyne **22** by base-induced elimination of two HBr molecules from **21** failed. We then tried to prepare **22** via hydrolysis of ester **20** [49,50], bromination of carboxylic acid **23** to obtain dibromo-acid **24** [51,52], and subsequent base-induced elimination of HBr from **24**. However, the final step—base-induced elimination of HBr—failed again. The unsuccessful elimination of HBr from dibromo compounds **21** and **24** was surprising, since this step was not reported as problematic in the literature syntheses of bicyclo[6.1.0]non-4-yne derivatives [30,47,48,49,50,51,52,53]. In contrast to a previous report [51], we did not encounter problems with the bromination and ester hydrolysis steps. Finally, we tried to synthesize *exo/endo*-[(1R*,8S*)-bicyclo[6.1.0]non-4-yn-9-yl]methanol (**27**) from ester **20** following the literature procedure [47,53]. In this case as well, the elimination step failed. Treatment of dibromo adduct **26** with *t*-BuOK in THF at 0 °C → 65 °C → 20 °C resulted in incomplete conversion, which did not improve after further treatment with LDA at –63 °C → 20 °C. Consequently, we were not able to isolate pure cyclooctyne **27** (Scheme 2). At this point, all further attempts to synthesize pure compounds **22** and **27** were abandoned, and we decided to use commercial [(1R*,8S*,9s)-bicyclo[6.1.0]non-4-yn-9-yl]methyl (2,5-dioxopyrrolidin-1-yl) carbonate (**2a**, see Figure 2) for cross-linking studies.

### 2.3. Synthesis of Azide- and Alkynyl-Functionalized Fluorecent Probes ***5*** and ***6***

**Scheme 3:**
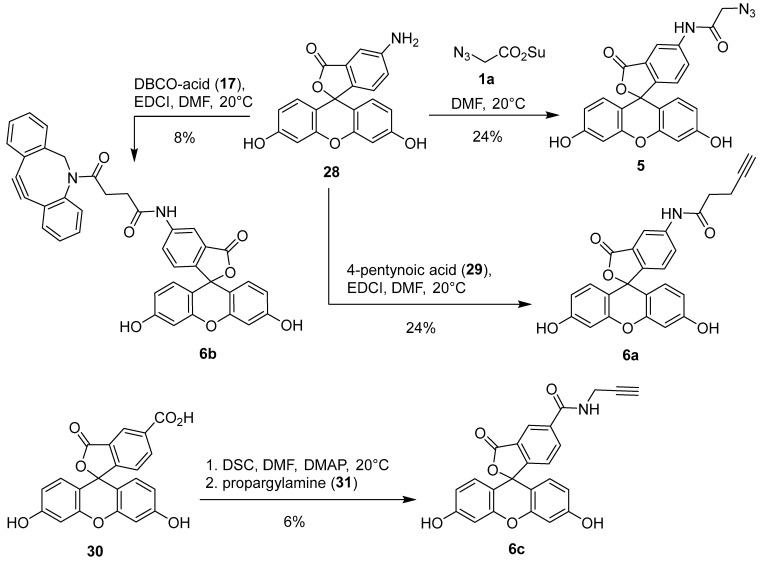
Synthesis of azide- and alkyne-functionalized fluorescein derivatives **5** and **6a**–**c**.

The synthesis of fluorescent probes **5** and **6** is shown in Scheme 3. Treatment of 6-aminofluorescein (**28**) with NHS ester **1a** gave the corresponding 2-azidoacetylfluorescein derivative **5** [33,34]. Similarly, acylation of **28** with 4-pentynoic acid (**29**) and DBCO-acid (**17**) in the presence of EDCI gave the alkynylated derivatives **6a** [35] and **6b**. As expected, due to lower nucleophilicity of the anilino group, the conversions in both *N*-acylation reactions were incomplete and the yields of the corresponding anilides **5** (24%), **6a** (24%), and **6b** (8%) were low. Therefore, fluorescent probe **6c** was also prepared by amidation of 6-carboxyfluorescein (**30**) following the literature procedure for the preparation of closely related fluorescein-6-carboxamides [54]. Compound **30** was first activated with di(succinimid-1-yl)carbonate (DSC) and the intermediate NHS ester was treated with excess propargylamine (**31**) to furnish the corresponding fluoresceine derivative **6c** [36,37], albeit in only 6% yield (Scheme 3).

### 2.4. Synthesis of Bis-Azide- (***3***) and Bis-Alkyne-Linkers ***4***

**Scheme 4:**
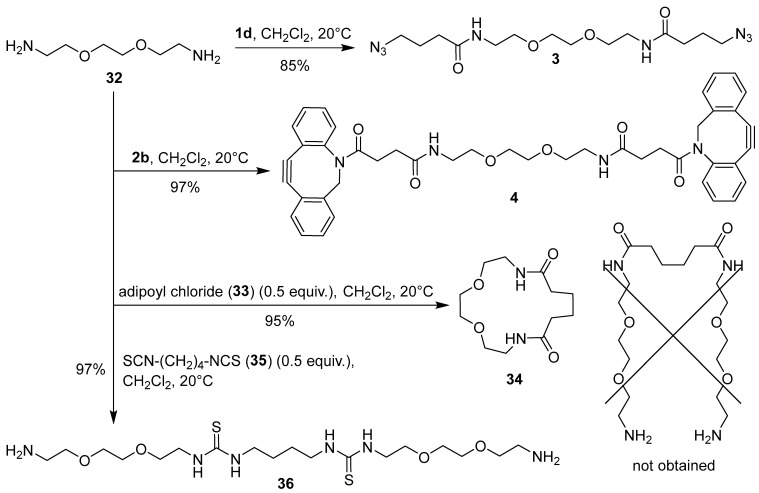
Synthesis of homobifunctional linkers **3**, **4**, **36**, and a macrocycle **34**.

The synthesis of homobifunctional linkers is shown in Scheme 4. Compounds **3** and **4** were prepared by treatment of 2,2′-(ethylenedioxy)bis(ethylamine) (**32**) with 2 equiv. of benzotriazolides **1d** and **2b**, respectively. Next, we attempted to prepare the ‘extended’ diamines by reacting adipoyl chloride (**33**) and butane-1,4-diisothiocyanate (**35**) with two equivalents of diamine **32**. Amidation of adipoyl chloride (**33**) with two equivalents of **32** following the literature procedure [55], gave macrocyclic 1,4-dioxa-7,14-diazacyclohexadecane-8,13-dione (**34**) as the only product in 95% yield. Notably, the reaction did not give the ‘extended’ diamine as reported in the literature [55]. At first glance, formation of macrocyclic compound **34** instead of the acyclic extended diamine was surprising. However, a further search of the literature revealed that **34** is a known compound, which had been obtained previously by reacting **32** with dimethyl adipate [56,57]. On the other hand, addition of two equivalents of diamine **32** to diisothiocyanate **35** gave the expected product **36** in quantitative yield (Scheme 4).

### 2.5. Covalent Binding of Compounds ***1*** and ***2*** to BSA Protein

Finally, compounds **1**–**6** were tested as bioconjugation reagents. Bovine serum albumin (**BSA**) was selected as the model protein. N-Acylation reagents **1** and **2** were used for attachment of azido and alkyne functionality to **BSA**, fluorescent probes **5** and **6** worked as “click” analytical reagents for determination of the amount of functional groups attached to azido and cycloalkyne modified **BSA**, and “click” bifunctional linkers **3** and **4** functioned as linkers for connecting two modified BSA.

**Scheme 5:**
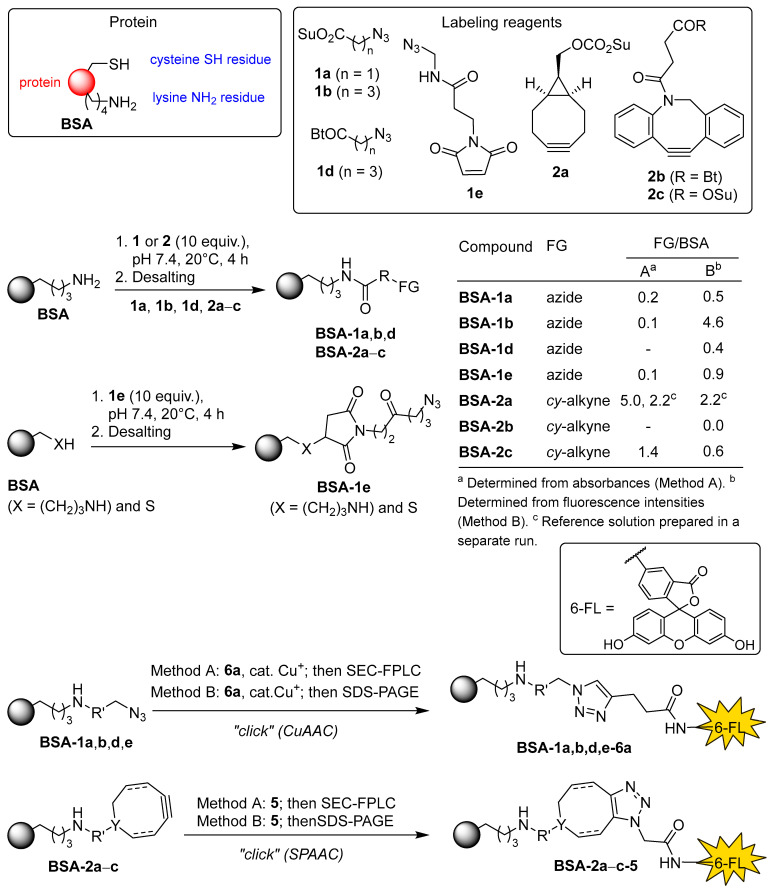
Functionalizing of **BSA** with azide and cyclooctyne functional groups and determination of molar loading of functional grops (FG/BSA) by Methods A and B.

The covalent binding of compounds **1** and **2** to **BSA** and the determination of the amount of functional group attached to **BSA** (i.e., functional group loading, FG/BSA) are presented in Scheme 5. First, azide-functionalized proteins **BSA-1a**, **BSA-1b**, **BSA-1e**, and cyclooctyne-functionalized proteins **BSA-2a** and **BSA-2c** were prepared by treating **BSA** with 10 equiv. of reagents **1a**, **1b**, **1e**, **2a**, and **2c** in PBS buffer at pH 7.4 and 20 °C for 4 h. To minimize competitive hydrolysis of NHS esters **1** and **2** in aqueous solution [1,40], **BSA** solution was mixed with solid reagents **1** and **2**, rather than with DMSO solutions of **1** and **2**. Another reason for using solid functionalization reagents **1** and **2** was to target a functional group loading of FG/BSA = 1. Since at least 30 of the 59 lysine residues in BSA were accessible, treatment with excess N-acylation reagent in a homogeneous phase might have resulted in excessive loading, FG/BSA > 1. To minimize this risk, solid reagents **1** and **2**, which can react only at the solid–liquid interface, were used. Next, aliquots of the crude functionalized proteins **BSA-1** and **BSA-2** were taken, and excess reagents and small molecular byproducts were removed by desalting. The purified **BSA-1** and **BSA-2** were then analyzed for functional group loading (FG/BSA). SPAAC reactions of cyclooctyne-conjugates **BSA-2a** and **BSA-2c** with azide-functionalized fluorescent probe **5** and CuAAC reactions of azide-conjugates **BSA-1a**, **BSA-1b**, and **BSA-1e** with alkyne-functionalized fluorescent probe **6a** were performed first, followed by purification by SEC-FPLC to yield fluorescently labeled proteins **BSA-1a-6a**, **BSA-1b-6a**, **BSA-1e-6a**, **BSA-2a-5**, and **BSA-2c-5**. Fractions containing the labeled proteins were collected, their absorbances were measured at 280 nm and 498 nm, and the corresponding FG/BSA values were then determined for each fraction based on the known molar absorbances of **BSA** at 280 nm and fluorescein derivatives **5** and **6** at 498 nm (Scheme 5, Method A). Molar loadings of azide-conjugates **BSA-1a**, **BSA-1b**, and **BSA-1e** were surprisingly low (~0.15), while molar loadings of cycloalkyne-functionalized conjugates **BSA-2a** and **BSA-2c** were 5.0 and 1.4, respectively (Scheme 5, Method A).

**Figure 3:**
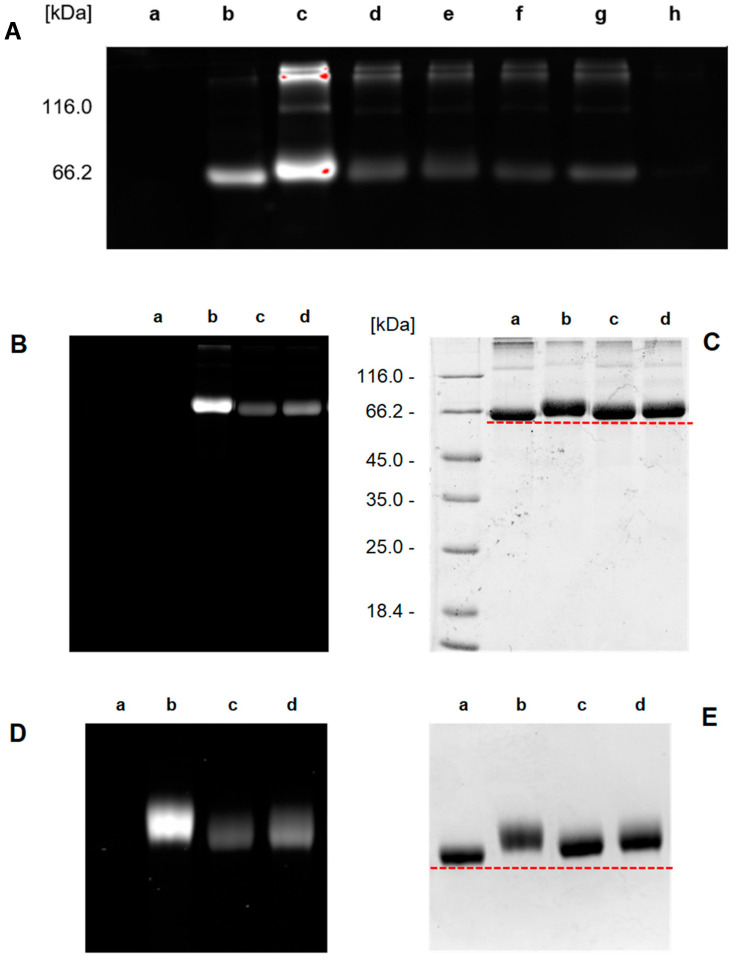
(**A**): SDS-PAGE of (a) **BSA** control, (b) reference standard **BSA-2a-6a** (FG/BSA = 2.2), (c) **BSA-1b-5**, (d) **BSA-1e-5**, (e) **BSA-1a-5**, (f) **BSA-1d-5**, (g) **BSA-2c-6a**, and (h) **BSA-2b-6a**. (**B**,**C**): SDS-PAGE (12% cross-linked gel) of (a) BSA control, (b) **BSA-1b-5**, (c) **BSA-1e-5**, and (d) **BSA-1a-5**. (**D**,**E**): Zoomed SDS-PAGE (7% cross-linked gel) of (a) unlabeled BSA control, (b) **BSA-1b-5**, (c) **BSA-1e-5**, and (d) **BSA-1a-5**. (**B**,**D**): Detection using setting for ProQ Emerald 300 detection. (**C**,**E**): Detection after staining with Coomassie Brilliant Blue using the corresponding detection settings. Red dotted line marks the lower edge of Mw of BSA control.

Since determination of FG/BSA following the above protocol was very time-consuming and laborious, it was not optimal for rapid FG/BSA analysis. Therefore, we decided to use a simplified procedure. First, a reference solution of fluorescently labeled conjugate **BSA-2a-6a** was prepared, FG/BSA = 2.2 was determined by Metod A and the conjugate was stored at 4 °C as a reference standard. Next, fluorescent conjugates **BSA-1a-5**, **BSA-1b-5**, **BSA-1d-5**, **BSA-1e-5**, **BSA-2b-6a**, and **BSA-2c-6a** were prepared and, together with the reference standard **BSA-2a-6a**, analyzed by SDS-PAGE. FG/BSA for conjugates **BSA-1a-5** (0.5), **BSA-1b-5** (4.6), **BSA-1d-5** (0.4), **BSA-1e-5** (0.9), **BSA-2b-6a** (0.0), and **BSA-2c-6a** (0.6) was determined based on their fluorescence intensities relative to the reference standard **BSA-2a-6a** (FG/BSA = 2.2) (Scheme 5, Method B). The obtained molar loading values were 0.4–0.9 in most cases. FG/BSA of **BSA-1b-5** was significantly higher (4.6), while coupling with benzotriazolide **2b** failed (FG/BSA = 0) (Scheme 5, Method B, Figure 3A). Low molar loadings (FG/BSA < 1) obtained after treating **BSA** with a large excess (10 equiv.) of coupling reagents **1** and **2** can be explained by incomplete conversion of solid reagents **1** and **2** due to their insolubility in aqueous media and concomitant partial hydrolysis, which competes with the N-acylation reaction. This explanation is supported by the observation that reagents **1** and **2** were not completely dissolved after stirring with **BSA** solution for 4 h. According to the FG/BSA values obtained, NHS esters **1a**, **1b**, **2a**, and **2c** were clearly superior to *N*-acylbenzotriazoles **1d** and **2b** as N-acylating reagents. Surprisingly, benzotriazolide **2b** was completely ineffective (FG/BSA ~ 0) under the coupling conditions employed (Scheme 5, Figure 3A, entry h). Successful functionalization of **BSA** should result in a slight (~1%) increase in the molecular weight of BSA (66.5 kDa) by 0.5–0.7 kDa due to the attached linker (coupling reagent) and fluorescent probe. As indicated with red dashed lines in Figure 3C,E, this increase in molecular weight was detectable on the SDS-PAGE gel, where spots of labeled **BSA** are shifted toward higher molecular weights. In addition, spots (b), (c), and (d) of labeled **BSA** are smeared or show dragging. This indicates that not all **BSA** molecules were functionalized with the same number of markers, resulting in a distribution of different molecular weights of products within a single spot (Figure 3D,E).

### 2.6. Attempted Covalent Cross-Linking of Azide- and Cyclooctyne-Functionalized Proteins BSA-1 and BSA-2

**Scheme 6:**
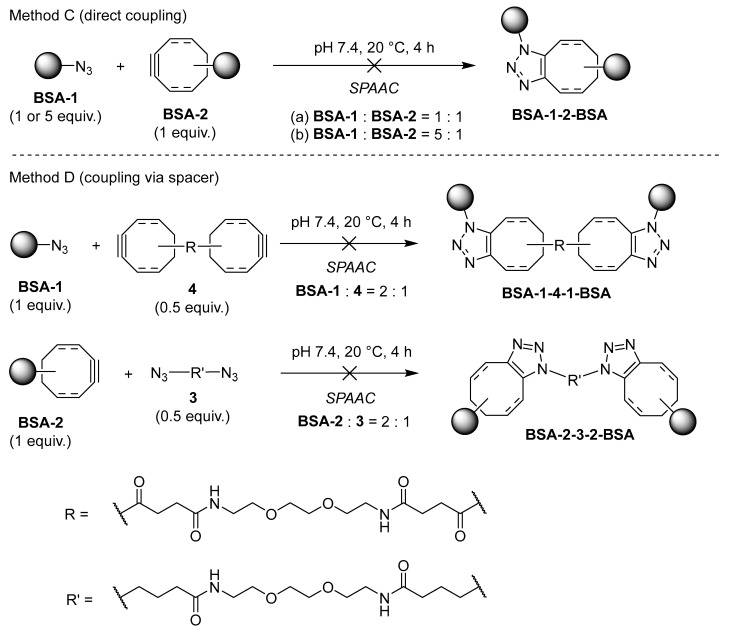
Methods C and D used in attempts to achieve covalent cross-linking of functionalized proteins **BSA-1** and **BSA-2**.

**Figure 4:**
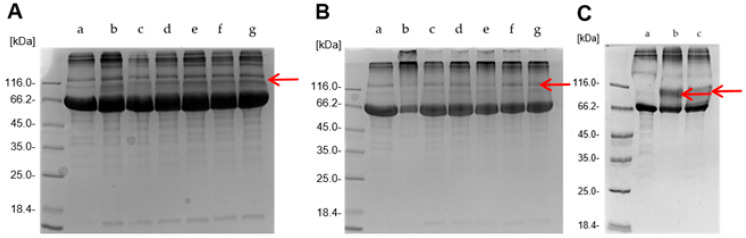
(**A**): SDS-PAGE of (a) **BSA** control, (b) **BSA-2a** + **BSA-1b** (1:1), (c) **BSA-2a** + **BSA-1e** (1:1), (d) **BSA-2a** + **BSA-1a** (1:1), (e) **BSA-2a** + **BSA-1b** (1:5), (f) **BSA-2a** + **BSA-1e** (1:5), and (g) **BSA-2a** + **BSA-1a** (1:5). (**B**): SDS-PAGE of (a) BSA control, (b) **BSA-2c** + **BSA-1b** (1:1), (c) **BSA-2c** + **BSA-1e** (1:1), (d) **BSA-2c** + **BSA-1a** (1:1), (e) **BSA-2c** + **BSA-1b** (1:5), (f) **BSA-2c** + **BSA-1e** (1:5), and (g) **BSA-2c** + **BSA-1a** (1:5). (**C**): SDS-PAGE of (a) **BSA** control, (b) **BSA-2c** + **3** (2:1), and (c) **BSA-1b** + **4** (2:1). (**A**–**C**): Detection after staining with Coomassie Brilliant Blue using the corresponding detection settings. Red arrows point to smeared spots at ~120 kDa corresponding to dimers of **BSA**.

We were interested only in controlled cross-linking of **BSA** that would preferentially yield the “covalent dimers” of **BSA**. We did not want any kind of uncontrolled oligomerization or cross-linking of **BSA** to be the major process. Accordingly, covalent cross-linking of azide- and cyclooctyne-functionalized molecules **BSA-1** and **BSA-2** was performed in two ways: by direct coupling of **BSA-1** and **BSA-2** to obtain **BSA-1-2-BSA** (Scheme 6, Method C), and by coupling azide-functionalized **BSA-1** with 0.5 equiv. of bis-cyclooctyne linker **4** and cyclooctyne-functionalized **BSA-2** with 0.5 equiv. of bis-azide linker **3** to obtain **BSA-1-4-1-BSA** and **BSA-2-3-2-BSA**, respectively (Scheme 6, Method D). First, Method C was tested. Cyclooctyne-functionalized proteins **BSA-2a** and **BSA-2c** (FG/BSA = 5.0 and 1.4, respectively) were treated with azide-functionalized proteins **BSA-1a**, **BSA-1b**, and **BSA-1e** (FG/BSA = 0.2, 0.1, and 0.1, respectively) in a molar ratio **BSA-2**: **BSA-1** = 1:1 and 1:5 at 20 °C for 4 h. In addition to the optimal molar ratio **BSA-2**: **BSA-1** = 1:1, a molar ratio of **BSA-2**: **BSA-1** = 1:5 was also tested to assess the potential effect of excess reagent on covalent dimers formation. The products were then analyzed by SDS-PAGE. In all cases, weak spots with Mw~120 kDa, corresponding to BSA dimers were observed. However, since the same spot with Mw~120 kDa was also observed with the **BSA** control, we concluded that covalent dimerization of **BSA-1** and **BSA-2** via the SPAAC reaction did not take place or occurred only to a small extent. Since similar results were obtained with both, 1:1 and 1:5 molar ratio of reactants, we concluded that the molar ratio did not influence the extent of dimerization (Figure 4A,B). We reasoned that unsuccessful direct dimerization might be due to the size of the protein molecule, which prevents the functional groups from getting close enough to react (Scheme 6, Method C). Therefore, we tested Method D, hoping that this difficulty could be overcome by using longer bifunctional linkers **3** and **4**, where each bifunctional reagent would react with two functionalized **BSA** molecules. Cyclooctyne-functionalized **BSA-2a** was treated with 0.5 equiv. of diazide **3** and azide-functionalized **BSA-1b** was treated with bis-cyclooctyne **4**. The reactions were carried out at room temperature for 24 h and then analyzed by SDS-PAGE. After comparing the spots of the non-functionalized **BSA** control (a) with those from the dimerization experiments (b and c), we concluded that most **BSA** molecules did not form dimers. Nevertheless, smeared spots with a molecular weight of approximately 120 kDa indicate that some dimerized molecules were probably formed (Figure 4C).

The failed covalent cross-linking can be explained as follows. Formation of covalent dimers is diffusion-controlled; therefore, very low concentrations of reactants significantly slow the reaction rate. The reacting functional groups are attached to the surface of **BSA** in a typical molar ratio of 1:1, while the protein is orders of magnitude larger than the attached functional group. Consequently, it is difficult for the azide and cyclooctyne groups of functionalized proteins **BSA-1** and **BSA-2** to come into close enough proximity with favorable geometry for cycloaddition to occur. As a result, the likelihood of a collision leading to covalent bond formation is further reduced.

## 3. Experimental

### 3.1. General Methods

Melting points were determined on a Kofler micro hot stage and on a Mettler Toledo MP30 automated melting point system (Mettler Toledo, Columbus, OH, USA). The NMR spectra were recorded in CDCl_3_ and DMSO-d_6_ using Me_4_Si as the internal standard on a Bruker Avance III Ultrashield 500 and Bruker Avance Neo 600 instruments (Bruker, Billerica, MA, USA) at 500 and 600 MHz for ^1^H and at 125 and 150 MHz for ^13^C nucleus, respectively. Chemical shifts (δ) are given in ppm relative to Me_4_Si as internal standard (δ = 0 ppm) and vicinal coupling constants (*J*) are given in hertz (Hz). HRMS spectra were recorded on an Agilent 6224 time-of-flight (TOF) mass spectrometer equipped with a double orthogonal electrospray source under atmospheric pressure ionization (ESI) coupled to an Agilent 1260 high-performance liquid chromatograph (HPLC) (Agilent Technologies, Santa Clara, CA, USA). UV-vis spectra were recorded in MeOH using a Varian Cary Bio50 UV-Visible Spectrophotometer (Agilent Technologies, Santa Clara, CA, USA). Emission spectra were recorded on a PerkinElmer LS 50 B Luminescence spectrophotometer (PerkinElmer, Waltham, MA, USA). Fourier-transform infrared (FT-IR) spectra were obtained on a Bruker FTIR Alpha Platinum spectrophotometer (Bruker, Billerica, MA, USA) using the attenuated total reflection (ATR) sampling technique. Microanalyses for C, H, and N were obtained on a Perkin-Elmer CHNS/O Analyzer 2400 Series II (PerkinElmer, Waltham, MA, USA). Column chromatography (CC) was performed on silica gel (Silica gel 60, particle size: 0.035–0.070 mm (Sigma-Aldrich, St. Louis, MO, USA). Acquisition and analysis of gels obtained after SDS-PAGE analysis and subsequent staining were performed on a Bio-Rad ChemiDoc MO Imaging System using BioRad Image Lab 6.1 Software for Windows (Bio-Rad, Hercules, CA, USA).

Unless otherwise stated, solutions in PBS buffer (10 mM Na_2_HPO_4_, 10 mM KH_2_PO_4_, 137 mM NaCl, 2.7 mM KCl, pH 7.4) were used. Prior to use, the commercial **BSA** was purified by loading the sample onto a HiLoad Superdex 200 pg preparative SEC column (Cytiva) connected to an ÄKTA FPLC system. The column was equilibrated in PBS buffer, pH 7.4, and a flow rate of 1 mL·min^−1^ was used to separate proteins. Proteins eluting at volumes corresponding to monomeric **BSA** were collected and stored at −80 °C until further use.

Ascorbic acid, bis(2,5-dioxopyrrolidin-1-yl) carbonate (DSC), bromine, *t*-BuOK, *t*-BuONa, 1,1′-carbonyldiimidazole (CDI), copper(II) sulfate, *N*-(3-dimethylaminopropyl)-*N*′-ethylcarbodiimide hydrochloride (EDCI), 4-(dimethylamino)pyridine (DMAP), *N*-hydroxysuccinimide, LiAlH_4_, lithium diisopropylamide (LDA), *N*-methylmorpholine (NMM), sodium azide, Rh(OAc)_2_, thionyl chloride, tris[(1-benzyl-4-triazolyl)methyl]amine (TBTA), tris(2-carboxyethyl)phosphine hydrochloride (TCEP), bovine serum albumin (**BSA**), 2,5-dioxopyrrolidin-1-yl 2-azidoacetate (**1a**), 2,5-dioxopyrrolidin-1-yl 4-azidobutanoate (**1b**), *N*-(3-azidopropyl)-3-(2,5-dioxo-2,5-dihydro-1*H*-pyrrol-1-yl)propanamide (**1e**), [(bicyclo[6.1.0]non-4-yn-9-yl)methyl] (2,5-dioxopyrrolidin-1-yl) carbonate (**2a**), 2,5-dioxopyrrolidin-1-yl 4-(11,12-didehydrodibenzo[*b,f*]azocin-5(6*H*)-yl)-4-oxobutanoate (**2c**), chloroacetic acid (**7a**), azidoacetic acid (**8a**), methyl 4-chlorobutyrate (**9b**), 1*H*-benzo[*d*][1,2,3]triazole (**11**), 6-aminocaproic acid (**12**), *tert*-butyl (2-bromoethyl)carbamate (**13**), succinic anhydride (**16**), 4-(11,12-didehydrodibenzo[*b,f*]azocin-5(6*H*)-yl)-4-oxobutanoic acid (**17**), *Z*,*Z*-1,5-cyclooctadiene (**18**), ethyl diazoacetate (**19**), 6-aminofluorescein (**28**), 4-pentynoic acid (**29**), 6-carboxyfluorescein (**30**), propargylamine (**31**), 2,2′-[ethane-1,2-diylbis(oxy)]bis(ethan-1-amine) (**32**), adipoyl chloride (**33**), and 1,4-diisothiocyanatobutane (**35**) are commercially available.

2,5-Dioxopyrrolidin-1-yl 2-azidoacetate (**1a**) [26], 3′,6′-dihydroxy-3-oxo-*N*-(prop-2-yn-1-yl)-3*H*-spiro[isobenzofuran-1,9′-xanthene]-6-carboxamide (**6c**) [36,37], azidoacetic acid (**8a**) [38], 4-azidobutanoic acid (**8b**), methyl 4-azidobutanoate (**10b**) [39], and *tert*-butyl (2-azidoethyl)carbamate (**14**) [42,43] were prepared following the literature procedures (see experimental procedures for the references).

Unless otherwise stated, solutions in PBS buffer (10 mM Na_2_HPO_4_, 10 mM KH_2_PO_4_, 137 mM NaCl, 2.7 mM KCl, pH 7.4) were used.

### 3.2. 2-Azidoacetic Acid (***8a***) [38]

This compound was prepared following a slightly modified general literature procedure for the synthesis of alkyl azides [38]. NaN_3_ (672 mg, 10.5 mmol) and ammonium chloride (1.06 g, 20 mmol) were added to a solution of chloroacetic acid (**7a**) (945 mg, 10 mmol) in water (7 mL) and the mixture was stirred at room temperature for 72 h. The reaction mixture was acidified with aq. HCl to pH 2 and the product was extracted with Et_2_O (3 × 20 mL). The combined organic phases were dried over anh. MgSO_4_, filtered, and the filtrate was evaporated in vacuo to give **8a**. Yield: 957 g (95%) of colorless oil. ^1^H NMR (500 MHz, CDCl_3_) δ 9.09 (br s, 1H), 4.14 (s, 2H). Spectral data are in agreement with the literature data [26,38].

### 3.3. 2,5-Dioxopyrrolidin-1-yl 2-Azidoacetate (***1a***) [26]

This compound was prepared following the literature procedure [26]. The reaction was carried out under argon. A mixture of azidoacetic acid (**8**) (508 g, 5 mmol), *N*-hydroxysuccinimide (862 g, 7.5 mmol), and anh. CH_2_Cl_2_ (15 mL) was stirred at 0 °C for 10 min. Then, *N*-[3-(dimethylamino)propyl]-*N*′-ethylcarbodiimide hydrochloride (EDCI) (1.438 g, 7.5 mmol) was added and stirring continued for another 10 min at 0 °C and then at room temperature for 24 h. The organic phase was washed with water (2 × 5 mL) and brine (5 mL), then dried over anh. Na_2_SO_4_, filtered, and the filtrate was evaporated in vacuo to give **1a**. Yield: 729 mg (73%) of white solid. ^1^H NMR (500 MHz, CDCl_3_) δ 4.24 (s, 2H), 2.87 (s, 4H). Spectral data are in agreement with the literature data [26].

### 3.4. Synthesis of Methyl 4-Azidobutanoate (***10b***) [39]

This compound was prepared following a slightly modified literature procedure [39]. Methyl 4-chlorobutanoate (**9b**) (609 μL, 682 mg, 5 mmol) was dissolved in DMSO (5 mL), NaN_3_ (975 mg, 15 mmol) was added, and the mixture was stirred at 80 °C for 12 h. The reaction mixture was cooled to room temperature, diluted with Et_2_O (20 mL), and washed with water (3 × 10 mL). The organic phase was dried over anh. MgSO_4_, filtered, and the filtrate was evaporated in vacuo to give **10b**. Yield: 603 mg (84%) of yellowish oil. ^1^H NMR (500 MHz, CDCl_3_) δ 3.69 (s, 3H), 3.35 (t, *J*= 6.7 Hz, 2H), 2.42 (t, *J*= 7.3 Hz, 2H), 1.91 (pentet, *J*= 7.0 Hz, 2H). ν_max_ (ATR) 2093 (N_3_), 1732 (C=O), 1437, 1164, 1080, 897 cm^−1^. Spectral data are in agreement with the literature data [39].

### 3.5. Synthesis of 4-Azidobutanoic Acid (***8b***) [39]

This compound was prepared following a modified literature procedure [39]. Methyl 4-azidobutanoate (**10b**) (603 mg, 4.2 mmol) was dissolved in MeOH (3 mL), 2 M aq. NaOH (4.2 mL, 8.4 mmol) was added and the mixture was stirred at 20 °C for 1 h. The reaction mixture was acidified with 1 M aq. HCl to pH 1, and the product was extracted with Et_2_O (3 × 20 mL). The combined organic phase was dried over anh. MgSO_4_, filtered, and the filtrate was evaporated in vacuo to give **8b**. Yield: 460 mg (83%) of yellowish oil. ^1^H NMR (500 MHz, CDCl_3_) δ 11.13 (br s, 1H), 3.38 (t, *J*= 6.7 Hz, 2H), 2.48 (t, *J*= 7.3 Hz, 2H), 1.92 (pentet, *J*= 7.0 Hz, 2H). Spectral data are in agreement with the literature data [39].

### 3.6. General Procedure for the Synthesis of N-Acylbenzotriazoles ***1c***, ***1d***, and ***2b***

Compounds **1c**, **1d**, and **2b** were prepared following the general literature procedure for the preparation of *N*-acylbenzotriazoles [41]. Under Ar, SOCl_2_ (150 µL, 2 mmol) was slowly added via syringe to a stirred solution of 1*H*-benzotriazole (1.00 g, 8 mmol) in anh. CH_2_Cl_2_ (50 mL) at r.t. and the mixture was stirred at r.t. for 30 min. Then, carboxylic acid **8a**, **8b**, or **17** (2 mmol) was added and a white precipitate that was formed within a few seconds was collected by filtration and washed with CH_2_Cl_2_ (2 × 10 mL). The combined filtrate was washed with 2 M aq. NaOH (30 mL), dried over anh. Na_2_SO_4_, filtered, and the filtrate was evaporated in vacuo to give **1c**, **1d**, and **2c**.

#### 3.6.1. 2-Azido-1-(1*H*-benzo[*d*][1,2,3]triazol-1-yl)ethan-1-one (**1c**) [28]

From 1*H*-benzo[*d*][1,2,3]triazole (**11**) (4.617 g, 38.8 mmol), SOCl_2_ (688 μL, 9.7 mmol), azidoacetic acid (**8a**) (980 mg, 9.7 mmol), the precipitate was washed with CH_2_Cl_2_ (2 × 50 mL), and the combined filtrate with 2 M aq. NaOH (3 × 60 mL). Yield: 322 mg (16%) of yellow solid, m.p. 56–64 °C. ^1^H NMR (500 MHz, CDCl_3_): δ 8.30 (ddt, *J* = 8.4, 4.2, 1.0 Hz, 1H), 8.17 (dt, *J* = 8.4, 0.9 Hz, 1H), 7.73 (ddt, *J* = 8.3, 7.0, 1.3 Hz, 1H), 7.57 (ddt, *J* = 8.1, 7.4, 0.9 Hz, 1H), and 5.20 and 5.02 (2s, 1:2, 2H). ^13^C NMR (126 MHz, CDCl_3_): δ 172.3, 138.5, 126.7, 115.0, 50.4. *m*/*z* (HRMS) Found: 120.0509 [MH–N_3_CH_2_CO]^+^. C_6_H_6_N_3_ requires *m*/*z* = 120.0556. ν_max_ (ATR) 2953, 2108, 1723 (C=O), 1413, 1278, 1202, 1012, 778, 737, 688 cm^−1^.

#### 3.6.2. 4-Azido-1-(1*H*-benzo[*d*][1,2,3]triazol-1-yl)butan-1-one (**1d**)

From 1*H*-benzo[*d*][1,2,3]triazole (**11**) (1.67 g, 14 mmol), SOCl_2_ (247 μL, 3.5 mmol), 4-azidobutanoic acid (**8b**) (452 mg, 3.5 mmol), the precipitate was washed with CH_2_Cl_2_ (2 × 15 mL), and the combined filtrate with 2M aq. NaOH (3 × 20 mL). Yield: 625 mg (78%) of yellow solid, m.p. 48–50 °C. ^1^H NMR (500 MHz, CDCl_3_): δ 8.28 (br d, *J* = 8.3 Hz, 1H), 8.13 (br d, *J* = 8.3 Hz, 1H), 7.67 (ddd, *J* = 8.1, 7.1, 0.9 Hz, 1H), 7.52 (ddd, *J* = 8.1, 7.0, 0.9 Hz, 1H), 3.55 (t, *J* = 7.2 Hz, 2H), 3.53 (t, *J* = 6.6 Hz, 2H), and 2.20 (pentet, *J* = 6.9 Hz, 2H). ^13^C NMR (126 MHz, CDCl_3_): δ 171.6, 146.3, 131.1, 130.6, 126.4, 120.3, 114.4, 50.6, 32.7, 23.7. *m*/*z* (HRMS) Found: 253.0813 [M + Na]^+^. C_10_H_10_N_6_NaO requires *m*/*z* = 253.0808. Anal. Calcd. for C_10_H_10_N_6_O: C, 52.17; H, 4.38; N, 36.50%. Found: C, 52.20; H, 4.12; N, 36.26%. ν_max_ (ATR) 2099, 1742 (C=O), 1485, 1445, 1365, 1287, 1260, 1165, 1065, 1003, 966, 854, 771, 755, 630 cm^−1^.

#### 3.6.3. *N*-[4-(11,12-Didehydro-5,6-dihydrodibenzo[*b,f*]azocin-5-yl)-4-oxobutanoyl]-1*H*-benzo[*d*][1,2,3]triazole (**2b**)

From 1*H*-benzo[*d*][1,2,3]triazole (**11**) (714 mg, 6 mmol), SOCl_2_ (106 μL, 1.5 mmol)), DBCO-acid (**17**) (457 mg, 1.5 mmol), the precipitate was washed with CH_2_Cl_2_ (2 × 5 mL), and the combined filtrate with 2M aq. NaOH (3 × 6 mL). Yield: 500 mg (82%) of pink solid, m.p. 160–163 °C. ^1^H NMR (500 MHz, CDCl_3_) δ 8.20 (d, *J* = 8.2 Hz, 1H), 8.08 (dd, *J* = 8.3, 0.9 Hz, 1H), 7.72–7.57 (m, 3H), 7.51–7.40 (m, 4H), 7.37–7.24 (m, 3H), 5.20 (d, *J* = 13.9 Hz, 1H), 3.80 (ddd, *J* = 18.4, 9.0, 5.1 Hz, 1H), 3.73 (d, *J* = 13.9 Hz, 1H), 3.38 (ddd, *J* = 18.4, 6.0, 5.0 Hz, 1H), 3.06 (ddd, *J* = 16.9, 9.0, 5.1 Hz, 1H), and 2.20 (dt, *J* = 17.0, 5.6 Hz, 1H). ^13^C NMR (126 MHz, CDCl_3_) δ 171.6, 171.2, 151.4, 147.9, 146.0, 132.1, 131.0, 130.2, 129.3, 128.6, 128.3, 127.8, 127.2, 126.0, 125.5, 123.1, 122.8, 120.1, 115.1, 114.3, 107.6, 55.6, 53.5, 30.9, 28.6. *m*/*z* (HRMS) Found: 407.1491 [M + H]^+^. C_25_H_19_N_4_O_2_ requires *m*/*z* = 407.1491. ν_max_ (ATR) 1727, 1652, 1479, 1448, 1387, 1350, 1307, 1226, 1165, 1063, 1005, 960, 802, 780, 765, 751, 649, 634 cm^−1^.

### 3.7. Synthesis of 6-(2-Azidoacetamido)hexanoic Acid (***8c***)

Under argon, CDI (2.00 g, 12.3 mmol) was added to a stirred solution of azidoacetic acid (**8a**) (1.22 g, 12.1 mmol) in anh. MeCN (30 mL) and the mixture was stirred at room temperature for 1 h. Then, 6-aminocaproic acid (**12**) (1.75 g, 13.3 mmol) was added and stirring under argon was continued for 2 h at room temperature and then for 24 h at 40 °C. Volatile components were evaporated in vacuo, the residue was dissolved in water (15 mL) and acidified with 1 M aq. HCl to pH 1, and the product was extracted with EtOAc (3 × 70 mL). The combined organic phase was dried over anh. Na_2_SO_4_, filtered, and the filtrate was evaporated in vacuo to give **8c**. Yield: 290 mg (11%) of yellow oil. ^1^H NMR (500 MHz, DMSO-*d*_6_) δ 12.23 (s, 1H), 8.19, 8.08, and 7.72 (3br t, 3:1:1, *J* = 5.5 Hz, 1H), 4.27, 4.02, 3.99, and 3.78 (4s, 3:6:1:2, 2H), 3.06 and 3.00 (2q, 4:1, *J* = 6.8 Hz, 2H), 2.19 and 2.02 (2t, 5:1, *J* = 7.4 Hz, 2H), 1.52–1.45 (m, 2H), 1.44–1.34 (m, 2H), 1.29–1.17 (m, 2H). ^13^C NMR (126 MHz, CDCl_3_) δ 174.9, 172.5, 172.3, 170.6, 169.1, 167.5, 166.2, 51.2, 49.9, 43.1, 42.0, 35.8, 34.0, 29.4, 29.1, 29.0, 26.4, 26.3, 25.5, 24.6, 21.5. Multiple signals for all nuclei are due to the presence of isomers (rotamers). *m*/*z* (HRMS) Found: 213.1003 [M − H]^−^. C_8_H_15_N_4_O_3_ requires *m*/*z* = 213.0993. ν_max_ (ATR) 2937, 2109 (N_3_), 1708 (C=O), 1631 (C=O), 1545, 1411, 1194, 1102, 927, 789, 639 cm^−1^.

### 3.8. Synthesis of tert-Butyl (2-Azidoethyl)carbamate (***14***) [42,43]

Compound **14** was prepared following a slightly modified literature procedure [42,43]. A mixture of *tert*-butyl (2-bromoethyl)carbamate (**13**) (1.12 g, 5 mmol), sodium azide (357 mg, 5.5 mmol), and anh. DMF (10 mL) was stirred under argon at 80 °C for 12 h. The mixture was cooled to room temperature, Et_2_O (50 mL) was added, and the solution was washed with brine (5 × 10 mL). The organic phase was evaporated in vacuo to give **14**. Yield: 723 mg (78%) of colorless oil. ^1^H NMR (500 MHz, CDCl_3_) δ 4.89 (br s, 1H), 3.39 (t, *J* = 5.6 Hz, 2H), 3.28 (q, *J* = 5.9 Hz, 2H), 1.43 (s, 9H). ν_max_ (ATR) 3349, 2978, 2933, 2095 (N_3_), 1687 (C=O), 1511, 1451, 1391, 1366, 1247, 1162, 1099, 1039, 992, 861, 781, 758, 637. cm^−1^. Spectral data are in agreement with the literature data [42,43].

### 3.9. 2-Azidoethan-1-aminium Chloride (***15***) [44]

Compound **15** was prepared following a modified literature procedure [44]. Compound **14** (723 mg, 3.88 mmol) was dissolved in EtOAc (6 mL) and cooled to 0 °C (ice-bath). While stirring at 0 °C, 2 M HCl (6 mL, 12 mmol) was added and stirring at 0 °C was continued for 2.5 h. The precipitate was collected by filtration, washed with EtOAc (2× 5 mL), and dried over NaOH pellets in vacuo at room temperature for 24 h to give **14**. Yield: 250 mg (68%) of white solid. ^1^H NMR (500 MHz, DMSO-*d*_6_) δ 8.26 (br s, 3H), 3.75–3.55 (m, 2H), 2.95 (dd, *J* = 6.6, 5.0 Hz, 2H). Spectral data are in agreement with the literature data [44,45].

### 3.10. Synthesis of 4-[(2-Azidoethyl)amino]-4-oxobutanoic Acid (***8d***) [46]

This compound was prepared following a modified literature procedure [46]. Succinic anhydride (**16**) (50 mg, 0.5 mmol) and *N*-methylmorpholine (NMM) (110 μL, 1 mmol) were added to a stirred suspension of compound **15** (60 mg, 0.5 mmol) in a mixture of anh. CH_2_Cl_2_ (1 mL) and anh. THF (1 mL) for 1 h at room temperature. KOH (28 mg, 0.5 mmol) was added and stirring at room teperature was continued for 24 h. Then, CH_2_Cl_2_ (10 mL) was added and the mixture was washed with 1 M aq. HCl (5 mL) and brine (2 × 5 mL). The organic phase was dried over anh. MgSO_4_, filtered, and the filtrate was evaporated in vacuo to give **8d**. Yield: 15 mg (16%) of yellow resin. ^1^H NMR (500 MHz, CDCl_3_) δ 6.01 (br s, 1H), 3.49–3.41 (m, 4H), 2.73 (dd, *J* = 7.3, 5.7 Hz, 2H), 2.53 (dd, *J* = 7.3, 5.7 Hz, 2H). Spectral data are in agreement with the literature data [46].

### 3.11. Synthesis of Ethyl (1R*,8S*,4Z)-Bicyclo[6.1.0]non-4-ene-9-carboxylate (***20***) [47]

This compound was prepared following the literature procedure [47]. A solution of ethyl diazoacetate (2.1 mL, 20 mmol) in CH_2_Cl_2_ (10 mL) was added slowly (dropwise over ~1 h) to a stirred mixture of *Z*,*Z*-1,5-cyclooctadiene (19.6 mL, 160 mmol), Rh(OAc)_4_ (380 mg, 0.86 mmol), and CH_2_Cl_2_ (10 mL) and the mixture was stirred at r.t. for 40 h. Insoluble material was removed by filtration through a glass frit and the filtrate was evaporated in vacuo. The residue was purified by CC (silica gel, petroleum ether). Fractions containing the product were combined and evaporated in vacuo to afford **20** as a C(9)-*endo*/*exo*-mixture of epimers. Yield: 3.22 g (83%) of colorless oil. ^1^H NMR (500 MHz, CDCl_3_) δ 5.63 and 5.60 (dq and td, 3:2, *J* = 4.1, 2.1 and 4.0, 2.3 Hz, 2H), 4.11 and 4.09 (2q, 2:3, *J* = 7.1 Hz, 2H), 2.50 and 2.30 (2 ddt, 2:3, *J* = 15.8, 8.0, 3.7 and 15.1, 7.6, 3.5 Hz, 1H), 2.24–2.15 (m, 2H), 2.12–2.01 (m, 2H), 1.82 (dddd, *J* = 14.4, 9.8, 7.1, 5.1 Hz, 1H), 1.71 and 1.18 (2t, 3:3, *J* = 8.8 and 4.6 Hz, 1H), 1.59–1.53 (m, 1H), 1.52–1.43 (m, 1H), 1.42–1.36 (m, 1H), 1.28–1.23 (m, 4H). Spectral data are in agreement with the literature data [47,53].

### 3.12. Synthesis of Ethyl (1R*,4R*,5R*,8S*)-4,5-Dibromobicyclo[6.1.0]non-4-ene-9-carboxylate (***21***) [48]

A solution of Br_2_ (320 mg, 2 mmol) in CH_2_Cl_2_ (2 mL) was added to a stirred solution of **20** (388 mg, 2 mmol) in CH_2_Cl_2_ (20 mL) and the solution was stirred at room temperature for 15 min. Then, the reaction was quenched by the addition of 10% aq. Na_2_S_2_O_3_ (5 mL). The phases were separated and the aqueous phase was extracted with CH_2_Cl_2_ (2 × 10 mL). The combined organic phase was dried over anh. MgSO_4_, filtered, and the filtrate was evaporated in vacuo to give **21** as a mixture of isomers. Yield: 642 mg (90%) of yellow resin. ^1^H NMR (500 MHz, CDCl_3_) δ 4.87–4.81 (m, 1H), 4.80–4.75 (m, 1H), 4.12 and 4.11 (2 q, 1:1, *J* = 7.1 Hz, 2H), 2.77–2.68 and 2.68–2.60 (2 m, 3:2, 2H), 2.37–2.24 (m, 2H), 2.23–2.06 (m, 2H), 1.88–1.60 (m, 3H), 1.53–1.37 (m, 1H), 1.29–1.18 (m, 4H). Spectral data are in agreement with the literature data [48].

### 3.13. Synthesis of (1R*,8S*,4Z)-Bicyclo[6.1.0]non-4-ene-9-carboxylic Acid (***23***) [49,50]

A 2 M aq. NaOH (2 mL, 4 mmol) was added to a solution of **20** (136 mg, 0.7 mmol) in EtOH (3 mL), the mixture was stirred at room temperature for 3 h, and acidified with 1 M aq. HCl to pH 2. The product was extracted with EtOAc (3 × 20 mL), the organic phases were combined, dried over anh. Na_2_SO_4_, filtered, and the filtrate was evaporated in vacuo to give **23** as a C(9)-*endo*/*exo*-mixture of epimers. Yield: 108 mg (93%) of colorless oil. ^1^H NMR (500 MHz, CDCl_3_) δ 11.77 (s, 1H), 5.60–5.54 (m, 2H), 2.42–2.34 (m, 1H), 2.29–2.21 (m, 1H), 2.14–2.06 (m, 2H), 2.06–1.95 (m, 2H), 1.79–1.70 (m, 1H), 1.59 and 1.14 (2 t, 1:2, *J* = 8.7 and 4.6 Hz, 1H), 1.52–1.42 (m, 1H), 1.41–1.29 (m, 2H). Spectral data are in agreement with the literature data [49,50].

### 3.14. Synthesis of (1R*,4R*,5R*,8S*)-4,5-Dibromobicyclo[6.1.0]non-4-ene-9-carboxylic Acid (***24***) [51,52]

This compound was prepared following a modified literature procedure [52]. A solution of Br_2_ (431 mg, 2.7 mmol) in CH_2_Cl_2_ (2 mL) was added to a stirred solution of **23** (450 mg, 2.7 mmol) in CH_2_Cl_2_ (20 mL) and the solution was stirred at room temperature for 15 min. Then, the reaction was quenched by the addition of 10% aq. Na_2_S_2_O_3_ (5 mL). The phases were separated and the aqueous phase was extracted with CH_2_Cl_2_ (2 × 10 mL). The combined organic phase was dried over anh. MgSO_4_, filtered, and the filtrate was evaporated in vacuo to give **24** as a mixture of isomers. Yield: 720 mg (82%) of colorless resin. ^1^H NMR (500 MHz, CDCl_3_) δ 11.92 (br s, 1H), 5.04–4.92 (m, 2H), 2.64–2.51 (m, 2H), 2.24–2.09 (m, 2H), 2.08–1.99 (m, 2H), 1.78–1.61 (m, 1H), 1.69 and 1.17 (2 t, 2:3, *J* = 8.7 and 4.1 Hz, 1H), 1.52–1.30 (m, 3H). Spectral data are in agreement with the literature data [51,52].

### 3.15. Synthesis of [(1R*,8S*,9RS,4Z)-Bicyclo[6.1.0]non-4-en-9-yl]methanol (***25***) [47]

This compound was prepared following the literature procedure [47]. Reaction was carried out under argon in a flame-dried flask. A solution of LiAlH_4_ in anh. THF (2.4 M, 500 μL, 1.2 mmol) was added via syringe to a stirred cold (0 °C, ice-bath) solution of **20** (194 mg, 1 mmol) in anh. Et_2_O (2 mL) and the mixture was stirred at 0 °C for 15 min and then at 45 °C for 1 h. The mixture was cooled to 0 °C and the reaction was quenched by slow (dropwise) addition of water (3 mL) to the stirred mixture. The obtained solution was diluted with THF (20 mL), dried over anh. Na_2_SO_4_, filtered, and the filtrate was evaporated in vacuo to give **25**. Yield: 111 mg (73%) of pale yellow resin. ^1^H NMR (500 MHz, CDCl_3_) δ 5.63 (td, *J* = 4.2, 2.1 Hz, 2H), 3.71 (d, *J* = 7.6 Hz, 1H), 3.47 (d, *J* = 6.9 Hz, 1H), 2.45–2.24 (m, 2H), 2.22–1.93 (m, 4H), 1.91–1.70 (m, 1H), 1.57 (m, 1H), 1.47–1.32 (m, 1H), 1.29–1.06 (m, 1H), 1.05–0.97 (m, 1H), 0.80–0.63 (m, 1H). Spectral data are in agreement with the literature data [47].

### 3.16. Synthesis of [(1R*,4R*,5R*,8S*,9RS)-4,5-Dibromobicyclo[6.1.0]non-4-en-9-yl]methanol (***26***) [47,53]

This compound was prepared following the literature procedure [47]. A solution of Br_2_ (37 μL, 115 mg, 0.73 mmol) in CH_2_Cl_2_ (2 mL) was added to a stirred solution of **25** (111 mg, 0.7 mmol) in CH_2_Cl_2_ (5 mL) and the solution was stirred at room temperature for 15 min. Then, the reaction was quenched by the addition of 10% aq. Na_2_S_2_O_3_ (5 mL). The phases were separated and the aqueous phase was extracted with CH_2_Cl_2_ (2 × 10 mL). The combined organic phase was dried over anh. MgSO_4_, filtered, and the filtrate was evaporated in vacuo to give **25**. Yield: 167 mg (77%) of yellowish resin. ^1^H NMR (500 MHz, CDCl_3_) δ 4.89–4.69 (m, 2H), 3.76 (dd, *J* = 7.5, 1.3 Hz, 1H), 3.52 (dd, *J* = 7.1, 1.9 Hz, 1H), 2.79–2.56 (m, 2H), 2.36–1.84 (m, 4H), 1.70–1.52 (m, 2H), 1.48–1.31 (m, 1H), 1.30–1.03 (m, 1H), 1.00–0.81 (m, 1H), 0.68 (m, 1H). Spectral data are in agreement with the literature data [47,53].

### 3.17. 2-Azido-N-(3′,6′-dihydroxy-3-oxo-3H-spiro[isobenzofuran-1,9′-xanthen]-6-yl)acetamide (***5***) [33,34]

NHS azidoacetate **1a** (99 mg, 0.5 mmol) was added to a stirred solution of **28** (174 mg, 0.5 mmol) in MeCN (3 mL) and the mixture was stirred at room temperature for 24 h. EtOAc (15 mL) was added, and the solution was washed with 1 M aq. NaHSO_4_ (5 mL) and brine (5 mL). The organic phase was dried over anh. MgSO_4_, filtered, and evaporated in vacuo. The residue (crude compound **5**, 165 mg) was suspended in CH_2_Cl_2_ (100 mL) and stirred at room temperature for 2 h. Then, stirring was stopped and the suspension was left to settle down. The supernatant was decanted and the solid residue was dried in vacuo at room temperature to give **5**. Yield: 52 mg (24%)(G.P.B) of brown solid. ^1^H NMR (500 MHz, DMSO-d_6_) δ 10.64 (s, 1H), 10.17 (br s, 2H), 8.30 (d, *J* = 2.0 Hz, 1H), 7.84 (dd, *J* = 8.3, 2.0 Hz, 1H), 7.25 (d, *J* = 8.3 Hz, 1H), 6.68 (d, *J* = 2.4 Hz, 2H), 6.60 (d, *J* = 8.6 Hz, 2H), 6.55 (dd, *J* = 8.6, 2.4 Hz, 2H), 4.14 (s, 2H). ^13^C NMR (126 MHz, CD_3_OD): δ ^13^C NMR (126 MHz, CD_3_OD) δ 165.4, 161.7, 159.5, 151.9, 144.7, 131.7, 120.7, 119.6, 118.8, 116.4, 106.9, 104.1, 101.9, 94.0, 43.8, 16.8. *m*/*z* (HRMS) Found: 431.0981 (MH^+^). C_22_H_15_N_4_O_6_ requires *m*/*z* = 431.0986. ν_max_ (ATR) 2208, 2104, 1695, 1584, 1531, 1453, 1384, 1237, 1204, 1169, 1109, 992, 909, 843, 759, 662 cm^−1^. Physical and spectral data are in agreement with the literature data [33,34].

### 3.18. N-(3′,6′-Dihydroxy-3-oxo-3H-spiro[isobenzofuran-1,9′-xanthen]-6-yl)pent-4-ynamide (***6a***) [35]

This compound was prepared following a modified literature procedure [35]. The reaction was carried out under argon in a flame-dried flask. A mixture of **28** (174 mg, 0.5 mmol), 4-pentynoic acid (**29**) (60 mg, 0.5 mmol), and anh. DMF (4 mL) was stirred at 0 °C (ice-bath) for 5 min. Then, EDCI (115 mg, 0.6 mmol) was added and stirring was continued for 10 min at 0 °C and then at room temperature for 24 h. EtOAc (20 mL) was added, and the solution was washed with 1 M aq. NaHSO_4_ (10 mL) and brine (10 mL). The organic phase was dried over anh. MgSO_4_, filtered, and the filtrate was evaporated in vacuo. The residue was purified by CC (hexanes–EtOAc, first 2:1 toe elute non-polar impurities, then 1:3 to elute the product). Fractions containing the product were combined and evaporated in vacuo to give **6a**. Yield: 52 mg (24%) of orange-brown solid. ^1^H NMR ^1^H NMR (500 MHz, MeOD) δ 7.15 (d, *J* = 2.0 Hz, 1H), 7.06 (dd, *J* = 8.3, 2.2 Hz, 1H), 6.88 (d, *J* = 8.2 Hz, 1H), 6.69–6.59 (m, 4H), 6.53 (dd, *J* = 8.7, 2.4 Hz, 2H), 2.82 and 2.66 (2 br t, 2:3, 1H), 2.62–2.57 (m, 1H), 2.51–2.47 (m, 1H), 2.46–2.41 (m, 1H), 2.36, 2.32, and 2.24 (3 t, 2:3:5, *J* = 2.6 Hz, 1H). Physical and spectral data are in agreement with the literature data [35].

### 3.19. 4-(11,12-Didehydro-5,6-dihydrodibenzo[b,f]azocin-5-yl)-N-(3′,6′-dihydroxy-3-oxo-3H-spiro[isobenzofuran-1,9′-xanthen]-6-yl)-4-oxobutanamide (***6b***)

The reaction was carried out under argon in a flame-dried flask. A mixture of DBCO-acid **17** (153 mg, 0.5 mmol) and anh. DMF was stirred at 0 °C (ice-bath) for 5 min. Then, EDCI (115 mg, 0.6 mmol) was added and stirring at 0 °C continued for 10 min. Next, 6-aminofluoresceine (**28**) (174 mg, 0.5 mmol) was added and the mixture was stirred at room temperature for 24 h. Most of DMF was evaporated in vacuo until approximately 1 mL of DMF left. EtOAc (20 mL) was added, and the solution was washed with 1 M aq. NaHSO_4_ (2 × 10 mL), brine (10 mL), and sat. aq. NaHCO_3_ (20 mL). The aqueous NaHCO_3_ phase was acidified with 2 M aq. HCl until pH 1 and the precipitate was collected by filtration to give **6b**. Yield: 25 mg (8%) of brown solid. ^1^H NMR (500 MHz, DMSO-d_6_) δ 10.35, 10.32, 8.28 and 8.23 (4 s, 1:3:1:3, 1H), 10.13 (s, 1H), 7.77–7.61 (m, 3H), 7.58–7.43 (m, 3H), 7.42–7.25 (m, 3H), 7.25–7.15 (m, 2H), 7.08–6.85 (m, 2H), 6.83–6.47 (m, 5H), 5.06 (dd, *J* = 14.1, 5.3 Hz, 1H), 3.65 (d, *J* = 14.1 Hz, 1H), 2.81–2.56 (m, 3H), 2.41–2.24 (m, 1H). ^13^C NMR (126 MHZ, CD_3_OD) δ 164.6, 163.8, 151.8, 151.4, 147.9, 147.7, 143.2, 143.1, 143.0, 139.9, 137.1, 125.1, 124.0, 123.9, 121.2, 121.1, 120.5, 120.5, 120.2, 120.2, 119.6, 119.4, 118.7, 117.0, 117.0, 114.4, 106.1, 104.3, 97.0, 94.0, 47.2, 23.3, 21.3. *m*/*z* (HRMS) Found: 635.1808 (MH^+^). C_39_H_27_N_2_O_7_ requires *m*/*z* = 635.1813. ν_max_ (ATR) 2151, 2043, 1754 (C=O), 1603, 1481, 1425, 1253, 1204, 1160, 1110, 1072, 993, 848, 752 cm^−1^.

### 3.20. 3′,6′-Dihydroxy-3-oxo-N-(prop-2-yn-1-yl)-3H-spiro[isobenzofuran-1,9′-xanthene]-6-carboxamide (***6c***) [36,37]

This compound was prepared according to a slightly modified literature procedure for the preparation of closely related fluorescein-6-carboxamides [54]. Et_3_N (418 μL, 303 mg, 3 mmol) and DMAP (6 mg, 50 μmol) were added to a stirred solution of carboxyfluorescein **30** (188 mg, 0.53 mmol) and DSC (282 mg, 1.1 mmol) in anh. DMF (8 mL) and the mixture was stirred at room temperature in dark for 1 h. Then, propargylamine (**31**) (86 μL, 69 mg, 1.25 mmol) was added and stirring in the dark at room temperature continued for 2 h. Volatile components were evaporated in vacuo, the residue was dissolved in EtOAc (20 mL), and the solution was washed with 1 M aq. NaHSO_4_ (2 × 10 mL) and brine (1 × 10 mL). The organic phase was dried over anh. Na_2_SO_4_, filtered, the filtrate was evaporated in vacuo, and the residue was purified by CC (silica gel, CH_2_Cl_2_–MeOH, 8:1). Fractions containing the product were combined and evaporated in vacuo. The crude product **6c** (25 mg, 12%) was dissolved in EtOAc (30 mL), the solution was transferred to a separatory funnel and shaken with sat. aq. NaHCO_3_ (3 × 15 mL). The combined aqueous phase was transferred to a separatory funnel, acidified with 2 M aq. HCl to pH 1, EtOAc (50 mL) was added, and the biphasic system was shaken and left to settle. The precipitate, which was formed between the organic and the aqueous phase, was collected by filtration to give **6c**. Yield: 12 mg (6%) of red solid. ^1^H NMR (500 MHz, DMSO-d_6_) δ 10.27 (br s, 2H), 9.35 (br t, *J* = 5.5 Hz, 1H), 8.47 (s, 1H), 8.26 (br d, *J* = 8.1 Hz, 1H), 7.38 (br d, *J* = 8.1 Hz, 1H), 6.72 (br s, 2H), 6.56 (br q, *J* = 8.7 Hz, 4H), 4.11 (br t, *J* = 3.8 Hz, 2H), 3.16 and 2.89 (2 br s, 1:1, 1H). ^13^C NMR (126 MHz, DMSO-d_6_) δ 169.0, 165.8, 159.9, 155.2, 152.3, 135.9, 135.2, 129.6, 126.9, 124.8, 124.1, 113.3, 109.4, 102.8, 90.4, 81.1, 73.3, 29.3. *m*/*z* (HRMS) Found: 414.0969 (MH^+^). C_24_H_16_NO_6_ requires *m*/*z* = 414.0972. Spectral data are in agreement with the literature data [36,37].

### 3.21. N,N′-{[Ethane-1,2-diylbis(oxy)]bis(ethane-2,1-diyl)}bis(4-azidobutanamide) (***3***)

Et_3_N (333 μL, 242 mg, 2.4 mmol) was added to a stirred solution of 2,2′-[ethane-1,2-diylbis(oxy)]bis(ethan-1-amine) (**32**) (147 μL, 148 mg, 1 mmol) and *N*-(4-azidobutanoyl)benzotriazole (**1d**) (470 mg, 2 mmol) in MeCN (10 mL) and the mixture was stirred at room temperature for 24 h. Volatile components were evaporated in vacuo and the residue was purified by CC. First benzotriazole was eluted with EtOAc–hexanes 1:1, followed by elution of the product with MeOH. Fractions containing the product were combined and evaporated in vacuo to give **3**. Yield: 316 mg (85%) of a yellowish solid, m.p. 40–55 °C. ^1^H NMR (500 MHz, CDCl_3_) δ 6.12 (br s, 1H), 3.60 (br s, 2H), 3.55 (br t, *J* = 5.2 Hz, 2H), 3.45 (br q, *J* = 5.4 Hz, 2H), 3.35 (br t, *J* = 6.6 Hz, 2H), 2.28 (t, *J* = 7.2 Hz, 2H), 1.92 (p, *J* = 7.0 Hz, 2H). ^13^C NMR (126 MHz, CDCl_3_) δ 171.9, 70.3, 69.9, 50.9, 39.3, 33.2, 24.9. *m*/*z* (HRMS) Found: 371.2153 (MH^+^). C_14_H_27_N_8_O_4_ requires *m*/*z* = 371.2150. ν_max_ (ATR) 3252, 2866, 2094, 1625 (C=O), 1564, 1457, 1422, 1341, 1253, 1122, 1031, 893, 853, 743 cm^−1^.

### 3.22. N,N′-{[Ethane-1,2-diylbis(oxy)]bis(ethane-2,1-diyl)}bis[4-oxo-4-(11,12-didehydro-5,6-dihydrodibenzo[b,f]azocin-5-yl)butanamide] (***4***)

Et_3_N (384 μL, 278 mg, 2.76 mmol) was added to a stirred solution of 2,2′-[ethane-1,2-diylbis(oxy)]bis(ethan-1-amine) (**32**) (169 μL, 170 mg, 1.15 mmol) and *N*-[4-oxo-4-(5-aza-3,4:7,8-dibenzocyclooctyn-5-yl)butananoyl]benzotriazole (**2b**) (934 mg, 2.3 mmol) in DMF (10 mL) and the mixture was stirred at room temperature for 24 h. Volatile components were evaporated in vacuo and the residue was purified by CC. First benzotriazole was eluted with EtOAc–EtOH 10:1, followed by elution of the product with MeOH. Fractions containing the product were combined and evaporated in vacuo to give **4**. Yield: 805 mg (97%) of yellow-brown resin. ^1^H NMR (500 MHz, CDCl_3_) δ 7.70–7.56 (m, 2H), 7.51–7.46 (m, 2H), 7.39–7.32 (m, 6H), 7.30–7.15 (m, 6H), 6.36 and 6.31 (2t, 1:1, *J* = 5.5 Hz, 2H), 5.11 and 5.08 (2s, 1:1, 2H), 3.61 (dd, *J* = 13.8, 2.6 Hz, 2H), 3.57–3.48 (m, 4H), 3.47–3.36 (m, 4H), 3.35–3.23 (m, 4H), 2.81–2.70 (m, 2H), 2.41–2.31 (m, 2H), 2.12 and 2.03 (2dt, 1:1, *J* = 15.1, 6.1 Hz, 2H), 1.91 and 1.84 (2dt, 1:1, *J* = 16.8, 6.0 Hz, 2H). ^13^C NMR (126 MHz, CDCl_3_) δ 172.4, 172.4, 172.3, 172.3, 162.7, 151.5, 148.2, 148.2, 132.3, 132.3, 129.5, 129.5, 128.8, 128.7, 128.2, 128.2, 128.2, 127.8, 127.8, 127.1, 127.1, 125.6, 125.5, 123.3, 123.2, 122.6, 114.8, 114.8, 108.0, 70.3, 69.9, 69.8, 55.6, 50.8, 39.3, 39.3, 36.6, 31.5, 31.3, 31.1, 30.3, 30.2. Multiple signals for carbon nuclei are due to the presence of isomers (rotamers). *m*/*z* (HRMS) Found: 723.3162 (MH^+^). C_44_H_43_N_4_O_6_ requires *m*/*z* = 723.3177.

### 3.23. Synthesis of 1,4-Dioxa-7,14-diazacyclohexadecane-8,13-dione (***34***) [56,57]

The reaction was carried out under argon using a flame-dried flask and a rubber septum. Adipoyl chloride (**33**) (145 μL, 181 mg, 1 mmol) was dissolved in anh. CH_2_Cl_2_ (10 mL) and the stirred solution was cooled to 0 °C (ice-bath). Diamine **32** (292 μL, 296 mg, 2 mmol) was then added slowly via syringe at 0 °C. Ice-bath was removed and the obtained suspension was stirred at room temperature for 16 h. The precipitate was collected by filtration and dried in vacuo over NaOH pellets at room temperature for 24 h to give **34**. Yield: 245 mg (95%) of a yellowish solid, m.p. 111–131 °C, lit. Reference [56] m.p. 153–155 °C. ^1^H NMR (500 MHz, CDCl_3_) δ 6.07 (br s, 2H), 3.62 (br s, 4H), 3.59–3.54 (m, 4H), 3.49–3.43 (m, 4H), 2.27–2.22 (m, 4H), 1.72–1.65 (m, 4H). *m*/*z* (HRMS) Found: 259.1652 (MH^+^). C_12_H_23_N_2_O_4_ requires *m*/*z* = 259.1650. ν_max_ (ATR) 3291, 2868, 1635, 1552, 1460, 1350, 1296, 1177, 1139, 1097, 1033, 982, 937, 864, 823, 696 cm^−1^. Spectral data are in agreement with the literature data [56,57].

### 3.24. Synthesis of 1,1′-(Butane-1,4-diyl)bis(3-{2-[2-(2-aminoethoxy)ethoxy]ethyl}thiourea) (***36***)

Diamine **32** (292 μL, 296 mg, 2 mmol) was added to a solution of 1,4-diisothiocyanatobutane (**35**) (172 mg, 1 mmol) in Et_2_O (2 mL), the mixture was stirred at room temperature for 16 h, and volatile components were evaporated in vacuo to give **36**. Yield: 456 mg (97%) of a yellow resin. ^1^H NMR (500 MHz, CDCl_3_) δ 7.68 and 7.09 (2s, 1:2, 2H), 3.89–3.36 (m, 24H), 3.01–2.90 (m, 2H), 2.87 (t, *J* = 5.2 Hz, 2H), 2.47 br (s, 6H), 1.72–1.58 (br m, 4H). ^13^C NMR (126 MHz, CDCl_3_) δ ^13^C NMR (126 MHz, CDCl_3_) δ 183.6, 72.6, 70.3, 70.2, 70.1, 70.0, 68.1, 44.8, 44.2, 41.5, 40.6, 31.0, 26.4, 26.2, 25.7. Multiple signals for carbon nuclei are due to the presence of isomers (rotamers). *m*/*z* (HRMS) Found: 468.2555 (M^+^). C_18_H_40_N_6_O_4_S_2_ requires *m*/*z* = 468.2552. ν_max_ (ATR) 3260, 3067, 2863, 1544, 1344, 1283, 1096, 698 cm^−1^.

### 3.25. Preparation of Stock Solutions of Azide- and Cyclooctyne-Functionalized Proteins ***BSA-1*** and ***BSA-2*** and Determination of Molar Loading (FG/BSA) from Molar Absorbances (Method A)

First, stock solutions of **BSA-1a**, **BSA-1b**, **BSA-1e**, **BSA-2a**, and **BSA-2c** were prepared. Five 1.5 mL PP vials were charged with reagents **1a**, **1b**, **1e**, **2a**, and **2c** (1 mg, 5.0, 4.4, 4.0, 3.4, and 2.5 μmol, 10 equiv.) and a solution of **BSA** (33.2, 29.2, 26.4, 22.6, and 16.6 mg, 0.50, 0.44, 0.40, 0.34, and 0.25 μmol, 1 equiv.) in PBS buffer (1 mL) was added to each PP vial. The mixtures were stirred (400 min.^−1^) at 20 °C for 24 h. Reagent **1e** dissolved completely, while reagents **1a**, **1b**, **2a**, and **2c** remained partially undissolved. The obtained stock solutions of **BSA-1a**, **BSA-1b**, **BSA-1e**, **BSA-2a**, and **BSA-2c** were stored at 4 °C. Next, aliquots (130 μL) of the crude **BSA-1** and **BSA-2** solutions were taken and excess small molecular reagents and byproducts were removed by desalting. To purified cyclooctyne-conjugates **BSA-2a** (44.2 nmol) and **BSA-2c** (32.5 nmol) excess fluorescent probe **5** was added (1.5 mM, 295 and 220 μL, 443 and 330 nmol, respectively, 10 equiv.). To the purified azide-conjugates **BSA-1a** (65 nmol), **BSA-1b** (57.2 nmol), and **BSA-1e** (52 nmol) excess alkyne-fluorescent probe **6a** (1.5 mM, 433, 370, and 345 μL, 650, 572, and 520 nm, respectively, 10 equiv.) and 1.25 μL of an aqueous solution of CuSO_4_ (10 mM, 12.5 nmol) and ascorbic acid (50 mM, 62.5 nmol) were added. The reaction mixtures were stirred (400 min^−1^) at 20 °C for 3 days, aliquots (350 μL) were taken and purified by SEC-FPLC. A Superdex 200 10/300 GL size exclusion column (formerly GE Healthcare Life Sciences, now Cytiva), equilibrated in PBS buffer (pH 7.4) and a flow rate of 0.5 mL·min^−1^, was used to purify the samples. Fractions containing the highest concentration of protein were collected to afford the fluorescently labeled proteins **BSA-1a-6a**, **BSA-1b-6a**, **BSA-1e-6a**, **BSA-2a-5**, and **BSA-2c-5**. Next, molar loading FG/BSA was determined for each labeled protein on the basis of absorbances measured at 280 nm and 498 nm (for FG/BSA values see Scheme 5, Method A). In a separate run, a solution of **BSA-2a-6a** with FG/BSA = 2.2 was prepared again to be used as a standard reference solution for rapid determination of FG/BSA from fluorescence intensities (see Method B, see Section 3.26).

### 3.26. Procedure for Rapid Determination of Molar Loading (FG/BSA) of Functionalized Proteins ***BSA-1*** and ***BSA-2*** from Fluorescence Intensities (Method B)

Stock solutions of the crude functionalized proteins **BSA-1a**,**b**,**d**,**e** and **BSA-2b**,**c** were prepared as described previously (see Section 3.25) by treatment of **BSA** with 10-fold excess reagents **1** and **2**. Also this time, only reagent **1e** dissolved completely, while reagents **1a**, **1b**, **2a**, **2b**, and **2c** remained partially undissolved. Aliquots (100 μL) of the crude **BSA-1** and **BSA-2** solutions were taken, excess small molecular reagents and byproducts were removed by desalting, and the purified **BSA-1** and **BSA-2** solutions were diluted with water to *c* ~ 2 mg mL^−1^ (~30 μM),and aliquoted (6 × 5 μL, 6 × ~0.15 nmol). To each aliquot PBS buffer containing 1% SDS (39 μL) was added, the mixture was heated at 90 °C for 10 min and cooled to room temperature. To each aliquot of cyclooctyne-conjugates **BSA-2b,c** large excess of azide-fluorescent probe **5** (2.5 μL, 50 mM, 125 nmol) was added. To each aliquot of azide-conjugates **BSA-1a,b,d,e** large excess of alkyne-probe **6a** (2.5 μL, 50 mM, 125 nmol) and 3.5 μL of an aqueous solution of CuSO_4_ (50 mM, 165 nmol), TCEP (100 mM, 330 nmol), and TBTA (3.4 mM, 11.9 nmol) was added. The reaction mixtures were stirred (400 min^−1^) in the dark for 1 h, followed by the addition of 4V (200 μL) of cold (0 °C) acetone and centrifugation (13,000× *g*, 4 °C, 2 min). The supernatants were decanted, and the proteins were analyzed by SDS-PAGE. To each fluorescently labeled protein 5 μL 4× SDS sample buffer, supplemented with 10% *v*/*v* β-mercaptoethanol (4× SDS+R), was added, the mixtures were stirred (400 min^−1^) at 37 °C for 30 min. Labeled proteins **BSA-1-5** and **BSA-2-6** and the reference standard solution **BSA-2a-6a** with known molar loading (FG/BSA = 2.2, see Section 3.25) were analyzed by SDS-PAGE. Afterwards, gels were briefly washed with deionized H_2_O and scanned with a ChemiDoc MP Imaging System (Bio-Rad) using settings for ProQ Emerald 300 detection. The same gels were subsequently stained with Coomassie Brilliant Blue, destained, and photographed using the same imager. The molar loading FG/BSA was then determined by analyzing the digital image data obtained (Emerald 300 and Coomasie Brilliant Blue) using Image Lab Software (Bio-Rad).

### 3.27. Procedure for Direct Covalent Cross-Linking of Functionalized Proteins ***BSA-1*** and ***BSA-2*** (Method C)

Solutions of the purified functionalized proteins **BSA-1a**, **BSA-1b**, **BSA-1e**, **BSA-2a**, and **BSA-2c** were prepared as described above (see Section 3.25) and their FG/BSA (0.2, 0.1, 0.1, 5.0, and 1.4, respectively) was determined by Method A (see Section 3.25 and Scheme 5). Twelve 1.5 mL PP vials were charged with purified cyclooctyne-conjugates **BSA-2a** and **BSA-2c** (6 × 100 μL each) and mixed with azide-conjugates **BSA-1a**, **BSA-1b**, and **BSA-1e** (2 × 100 μL each and 2 × 500 μL each). The mixtures were stirred at 20 °C for 4 h and then analyzed by SDS-PAGE. Afterwards, gels were briefly washed with deionized H_2_O, stained with Coomassie Brilliant Blue, destained and photographed with a ChemiDoc MP Imaging System (Bio-Rad).

### 3.28. Procedure for Covalent Cross-Linking of Functionalized Proteins ***BSA-1b*** and ***BSA-2a*** Using Bifunctional Linkers ***3*** and ***4*** (Method D)

Solutions of the purified functionalized proteins **BSA-1b** and **BSA-2a** were prepared (see Section 3.25) and their FG/BSA (4.6 and 2.2, respectively) was determined by Method B (see Section 3.26 and Scheme 5). A 1.5 mL PP vial was charged with the purified azide-conjugate **BSA-1b** (200 μL, 439 μM, 87.8 nmol; FG/BSA = 4.6, n_(azide)_ = 404 nmol, 1 equiv.) and a solution of bis-cyclooctyne **4** in DMSO (72.9 μL, 2.77 mM in DMSO, 202 nmol, 0.5 equiv.). In the same manner, cyclooctyne conjugate **BSA-2a** (200 μL, 341 μM, 68.3 nmol; FG/BSA = 2.2, n_(cyclooctyne)_ = 150.3 nmol, 1 equiv.) was mixed with bis-azide **3** in PBS buffer (13.9 μL, 5.4 mM, 75.1 nmol, 0.5 equiv.). Both reaction mixtures were shaken at room temperature for 24 h and SDS-PAGE analysis was performed. Afterwards, gels were briefly washed with deionized H_2_O, stained with Coomassie Brilliant Blue, destained, and photographed with a ChemiDoc MP Imaging System (Bio-Rad).

## 4. Conclusions

NHS esters and benzotriazolides of azide- and cyclooctyne-functionalized carboxylic acids **1** and **2** were prepared and used as reagents for functionalization of primary amino groups of lysine’s’ side chains of **BSA** protein, 6-aminofluorescein (**28**), and 2,2′-[ethane-1,2-diylbis(oxy)]bis(ethan-1-amine) (**32**). It turned out in the course of this study that performing the syntheses of cyclooctyne and fluorescein derivatives to obtain bioconjugation reagents and fluorescent probes may sometimes be challenging. Acquiring specific skills is necessary for successful reproduction of known synthetic procedures. On the other hand, the biochemistry part of this study was relatively predictable. Treatment of **BSA** with excess reagents **1** and **2** gave the desired azido and cyclooctyne groups in functionalized proteins **BSA-1** and **BSA-2**. The amount of azide and cyclooctyne groups attached was determined from the absorbances and fluorescence intensities of cycloadducts **BSA-1-6** and **BSA-2-6** obtained by SPAAC and CuAAC reactions with complementary functionalized fluorescent probes **5** and **6**. Covalent binding of functionalized **BSA** was performed by direct SPAAC between **BSA-1** and **BSA-2** and by binding **BSA-1** or **BSA-2** through SPAAC with 0.5 equiv. of complementary bis-azide and bis-cyclooctyne linkers **4** and **3**. SDS-PAGE analysis showed weak spots with M_w_~120 kDa corresponding to dimers of **BSA** in all dimerization attempts. However, further optimization is required to obtain covalent dimers as the major products.

## Data Availability

The data presented in this study are available in the main manuscript and in the Supplementary Materials of this manuscript. Further inquiries can be directed to the corresponding authors.

## Supplementary Materials

The following supporting information can be downloaded at: https://www.mdpi.com/article/10.3390/molecules30234623/s1, File S1: Copies of ^1^H and ^13^C NMR spectra.
