# Supplementary material for: Synthesis of Bioconjugation Reagents for Use in Covalent Cross-Linking of Proteins by Azide-Alkyne Cycloaddition
Source: Molecules. 2025 Dec 2;30(23):4623. doi: 10.3390/molecules30234623 (PMC12693261; doi:10.3390/molecules30234623)
Supplement: Supplementary file 1 [file molecules-30-04623-s001.zip › molecules-4010416-Supplementary File S1.pdf]

molecules

Article

Synthesis of Bioconjugation Reagents for Use in Covalent Cross-Linking of Proteins by Azide–Alkyne Cycloaddition

Nadja Suhorepec, Luka Ciber, Uroš Grošelj, Nejc Petek, Bogdan Štefane, Marko Novinec*, and Jurij Svete*

Faculty of Chemistry and Chemical Technology, University of Ljubljana, Večna pot 113, 1000 Ljubljana, Slovenia
* Correspondence: marko.novinec@fkkt.uni-lj.si (M.N.) and jurij.svete@fkkt.uni-lj.si (J.S.); Tel.: +386-1-479-8562 (J.S.)

Table of Contents.

1. Copies of NMR spectra . S2

 https://doi.org/10.3390/molecules30234623

1. Copies of NMR spectra.

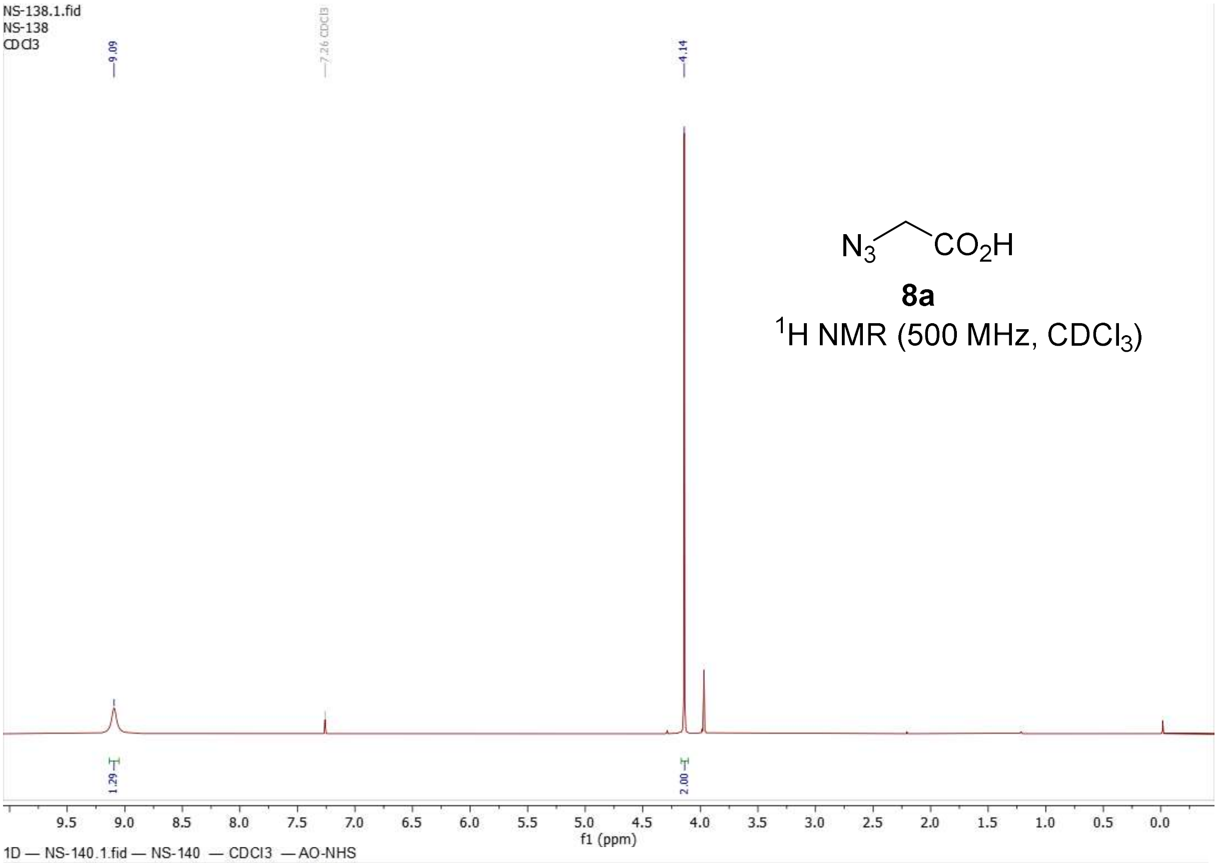

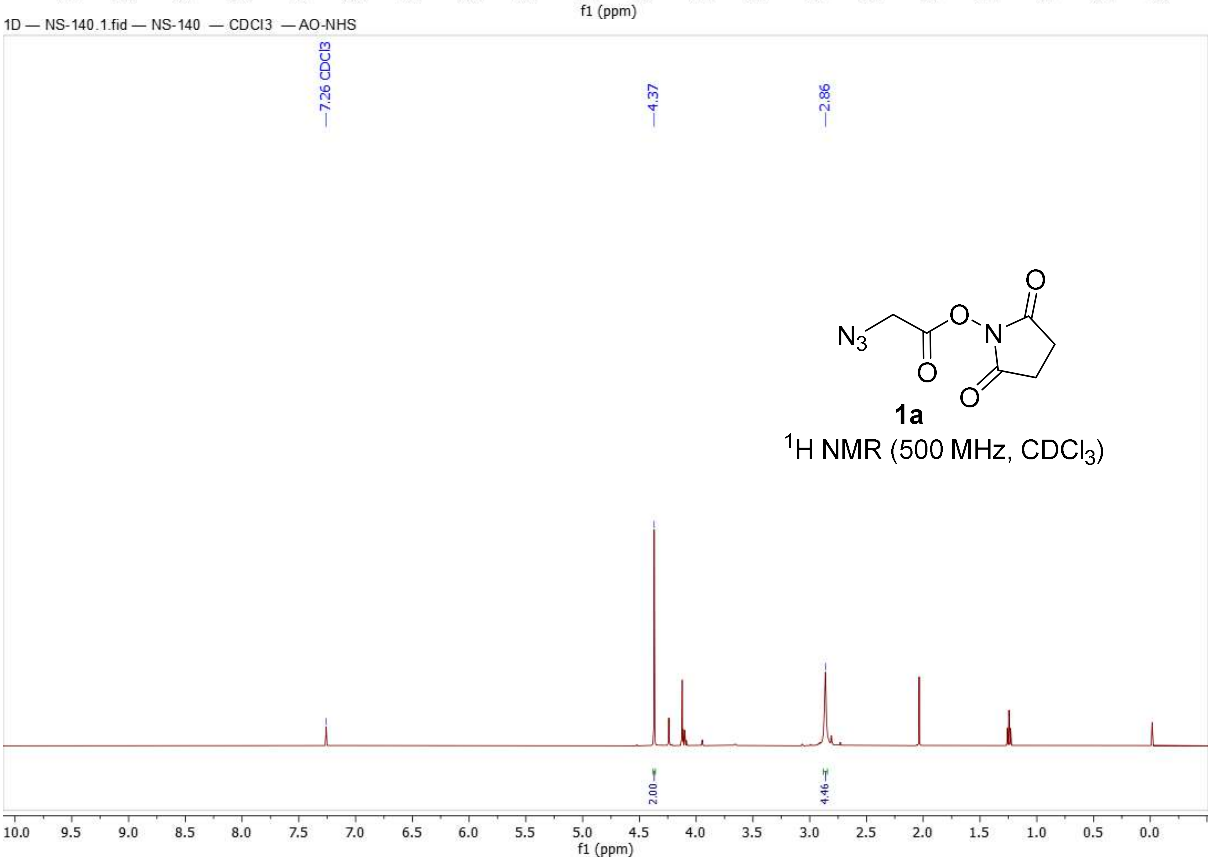

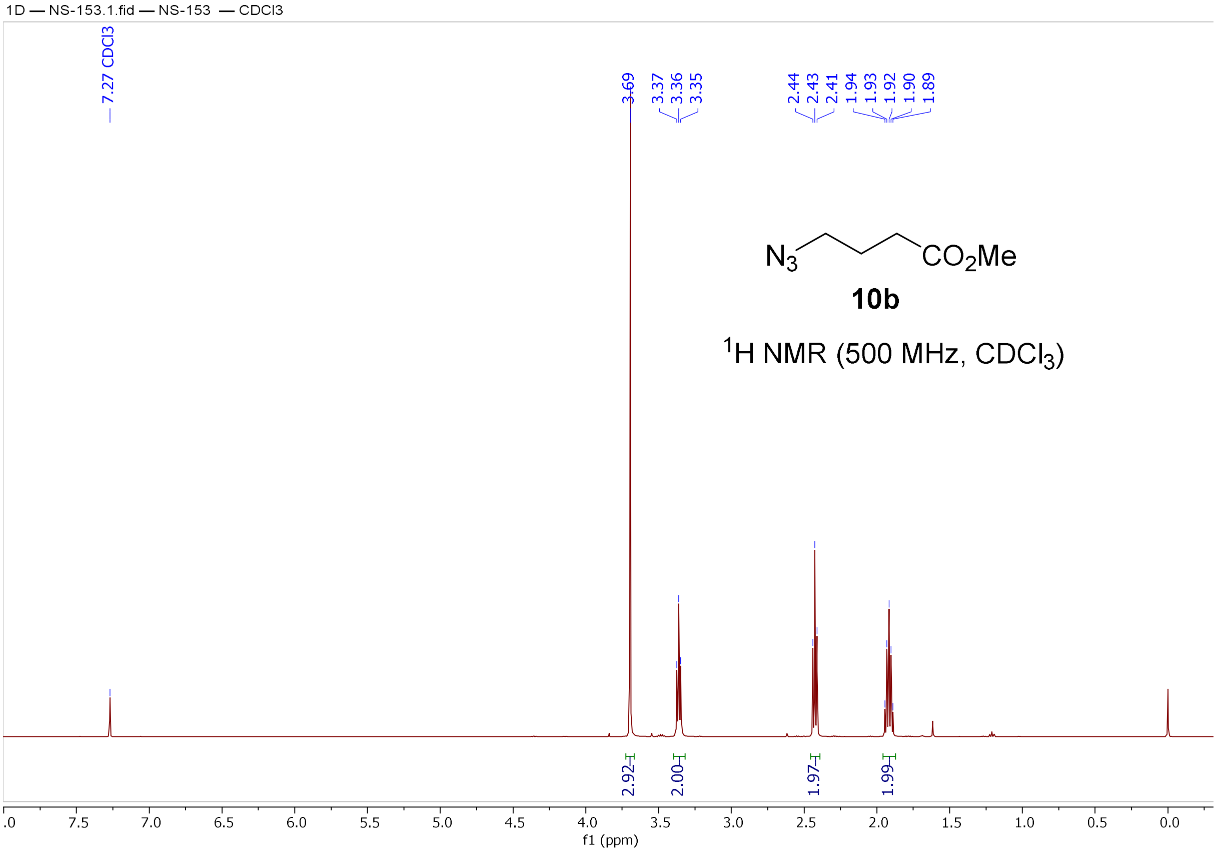
1D — NS-153.1.fid — NS-153 — CDCl3
7.27 CDCl3
3.69
3.37
3.36
3.35
2.44
2.43
2.41
1.94
1.93
1.92
1.90
1.89
N3
CO2Me
10b
1H NMR (500 MHz, CDCl3)
2.92
2.00
1.97
1.99
f1 (ppm)

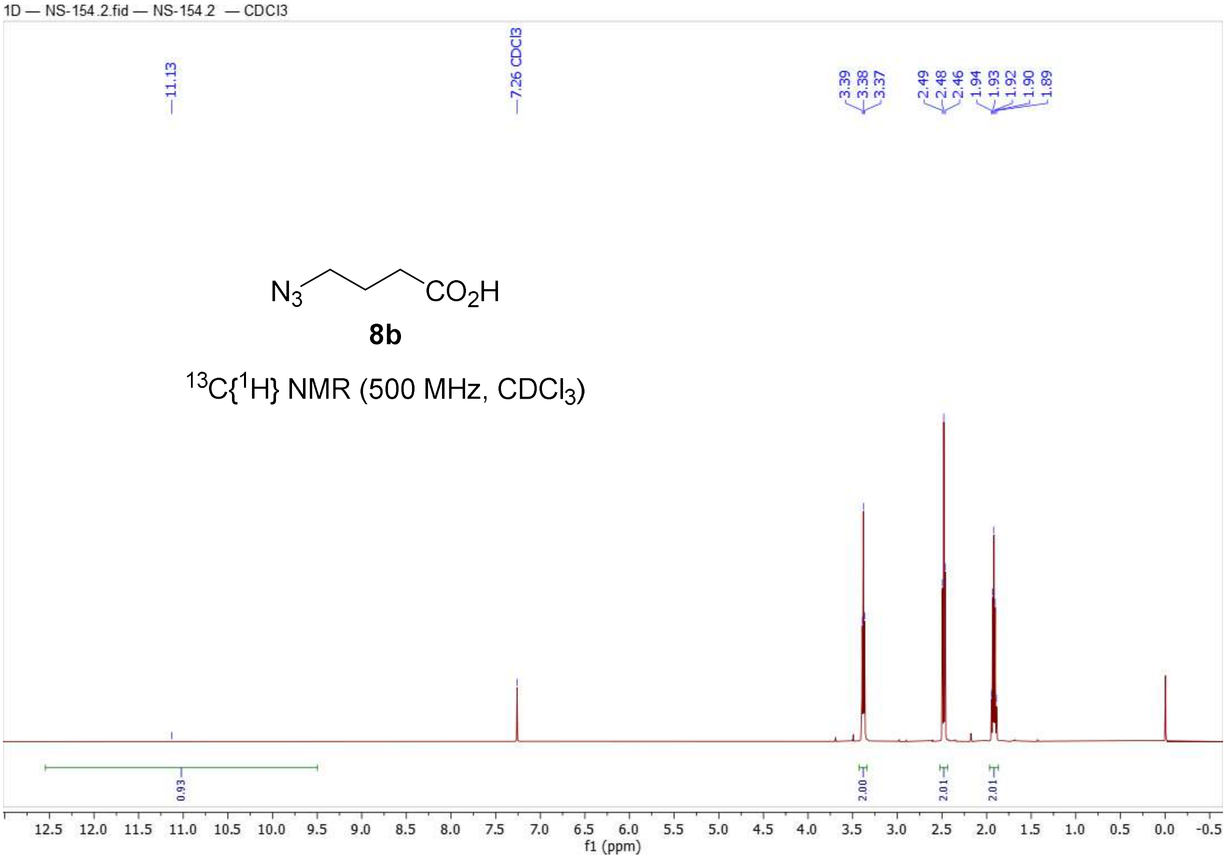
1D — NS-154.2.fid — NS-154.2 — CDCl3
11.13
7.26 CDCl3
3.39
3.38
3.37
2.49
2.48
2.46
1.94
1.93
1.92
1.90
1.89
N3
CO2H
8b
13C{1H} NMR (500 MHz, CDCl3)
0.93
2.00
2.01
2.01
f1 (ppm)

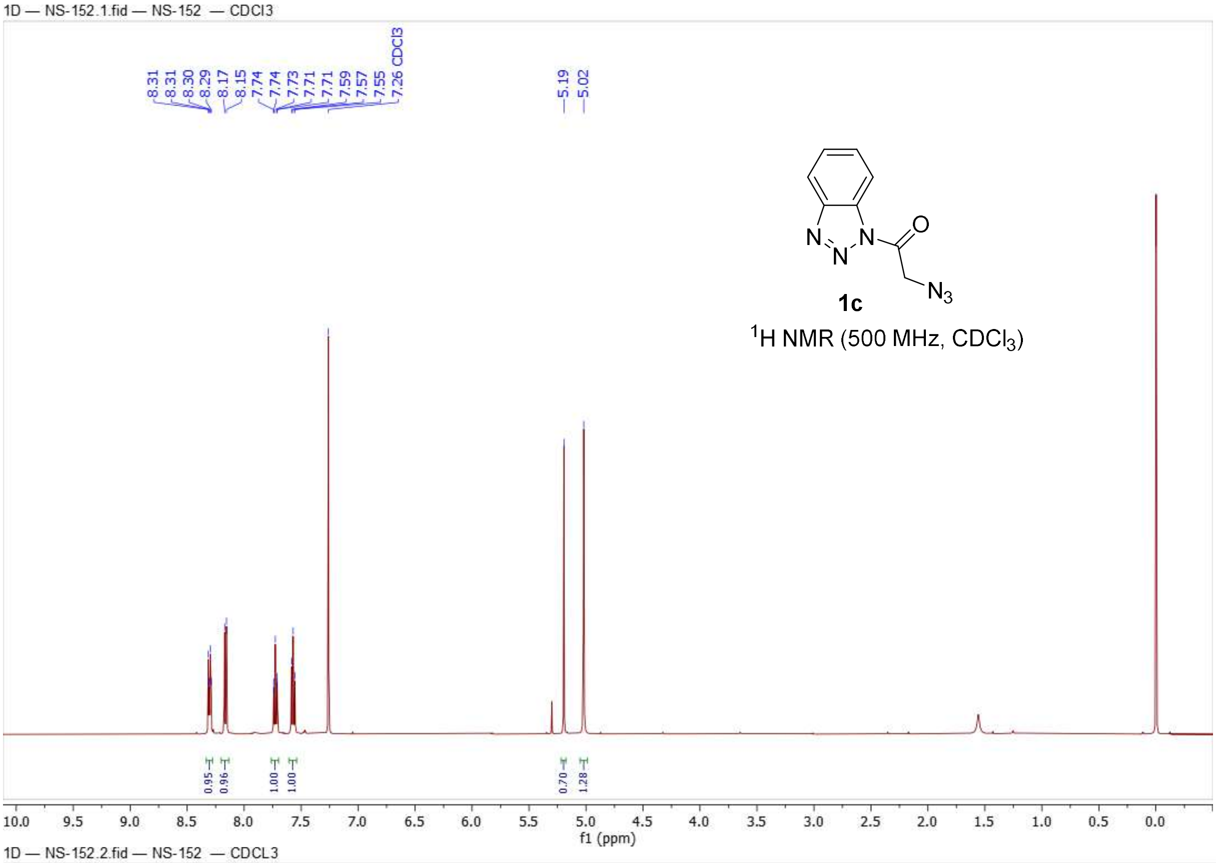
1D — NS-152.1.fid — NS-152 — CDCl3
8.31
8.31
8.30
8.29
8.17
8.15
7.74
7.74
7.73
7.71
7.71
7.59
7.57
7.55
7.26 CDCl3
5.19
5.02
N
N
N
N
O
N3
1c
1H NMR (500 MHz, CDCl3)
0.95
0.96
1.00
1.00
0.70
1.28
10.0 9.5 9.0 8.5 8.0 7.5 7.0 6.5 6.0 5.5 5.0 4.5 4.0 3.5 3.0 2.5 2.0 1.5 1.0 0.5 0.0
f1 (ppm)

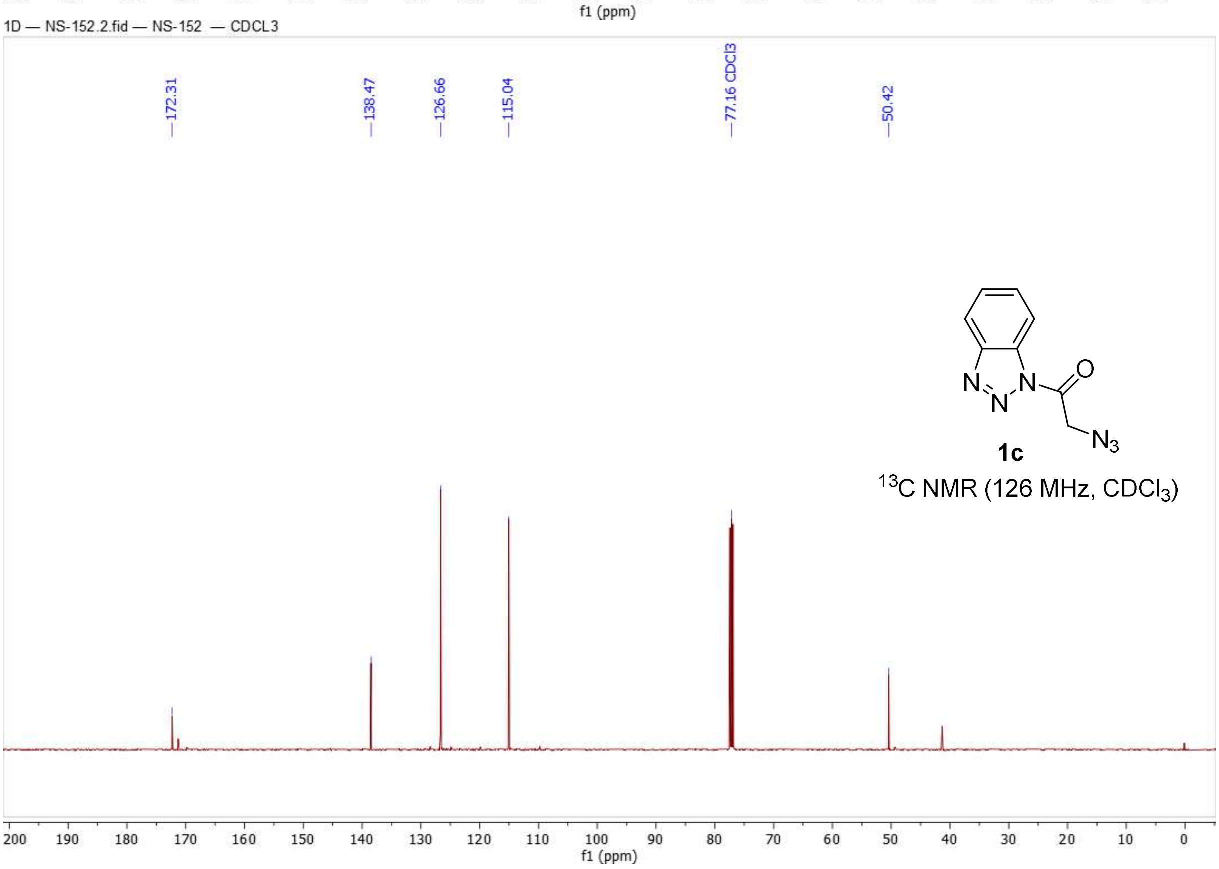
1D — NS-152.2.fid — NS-152 — CDCL3
172.31
138.47
126.66
115.04
77.16 CDCl3
50.42
N
N
N
N
O
N3
1c
13C NMR (126 MHz, CDCl3)
200 190 180 170 160 150 140 130 120 110 100 90 80 70 60 50 40 30 20 10 0
f1 (ppm)

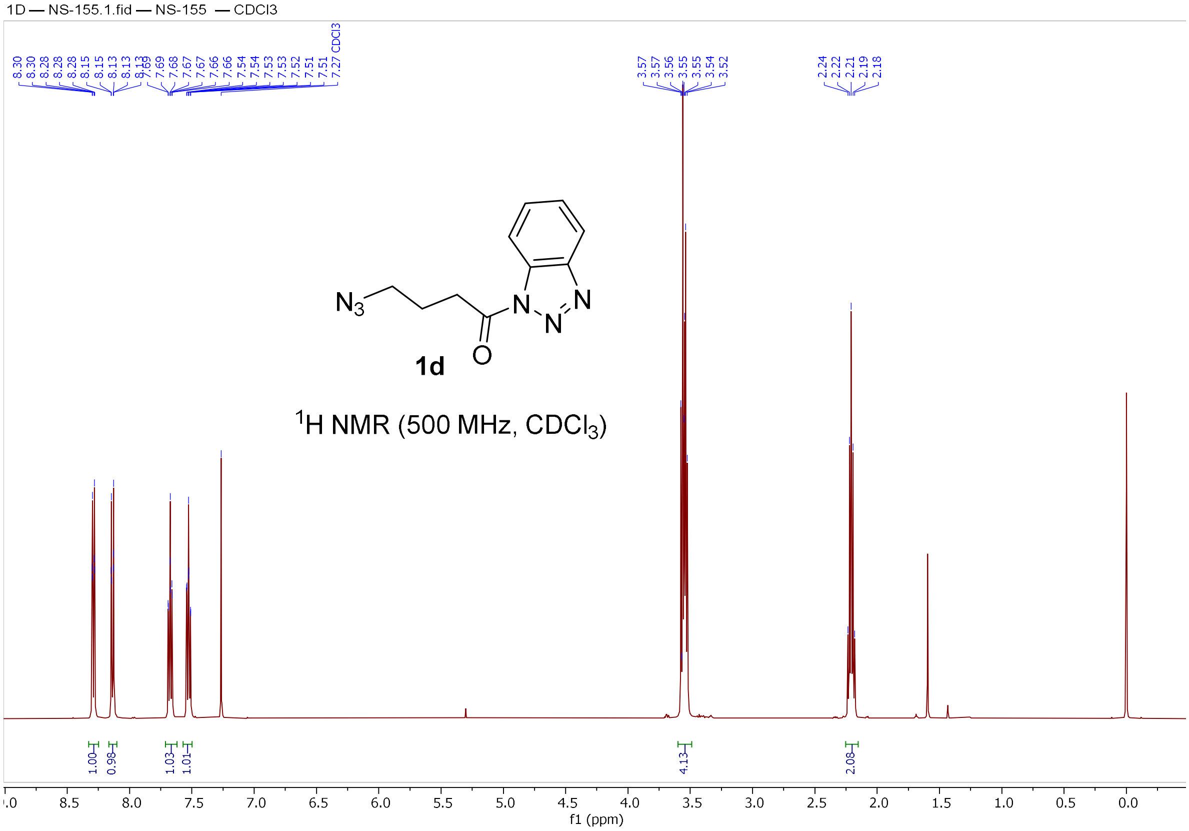
1D — NS-155.1.fid — NS-155 — CDCl3
8.30 8.30 8.28 8.28 8.28 8.15 8.15 8.13 8.13 8.13 7.69 7.69 7.68 7.67 7.67 7.66 7.66 7.54 7.54 7.53 7.53 7.52 7.51 7.51 7.27 CDCl3
3.57 3.57 3.56 3.55 3.55 3.54 3.54 3.52
2.24 2.22 2.21 2.19 2.18
N3
1d
1H NMR (500 MHz, CDCl3)
1.00 0.98 1.03 1.01 4.13 2.08
f1 (ppm)

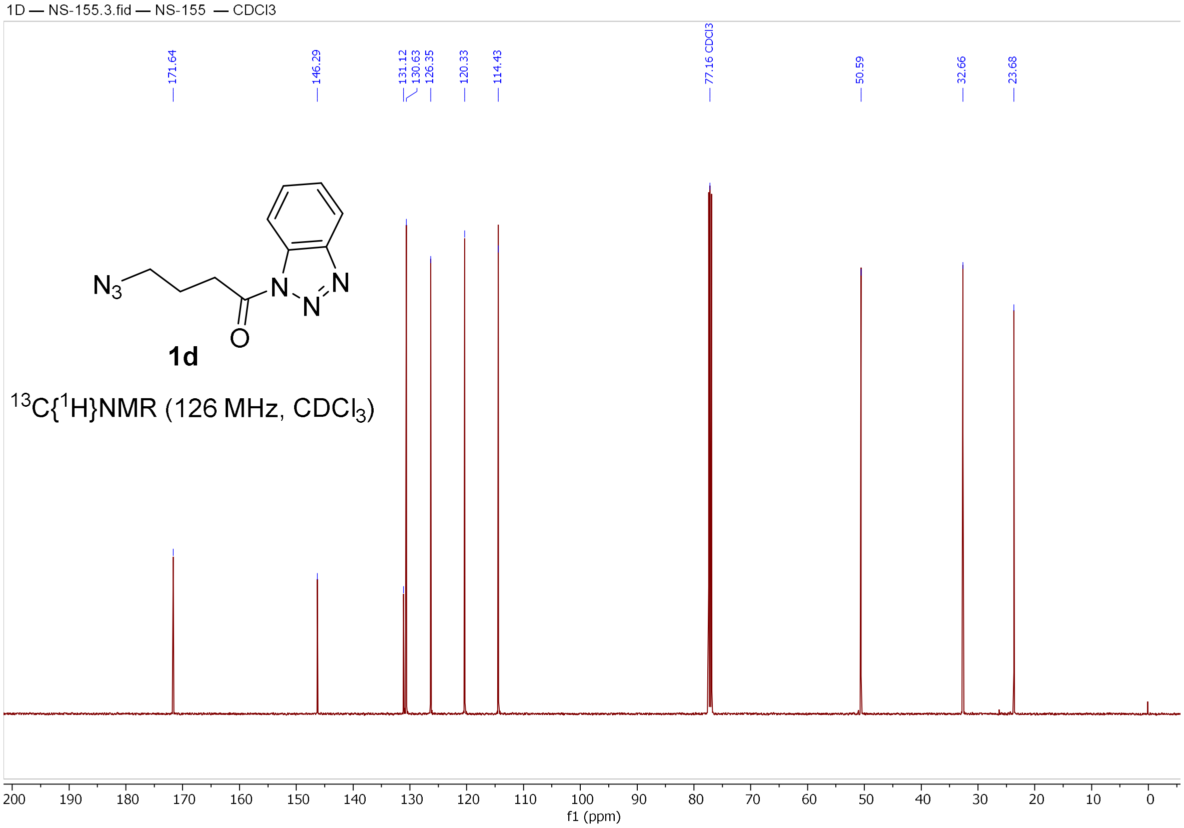
1D — NS-155.3.fid — NS-155 — CDCl3
171.64
146.29
131.12 130.63 126.35
120.33
114.43
77.16 CDCl3
50.59
32.66
23.68
N3
1d
13C{1H}NMR (126 MHz, CDCl3)
f1 (ppm)

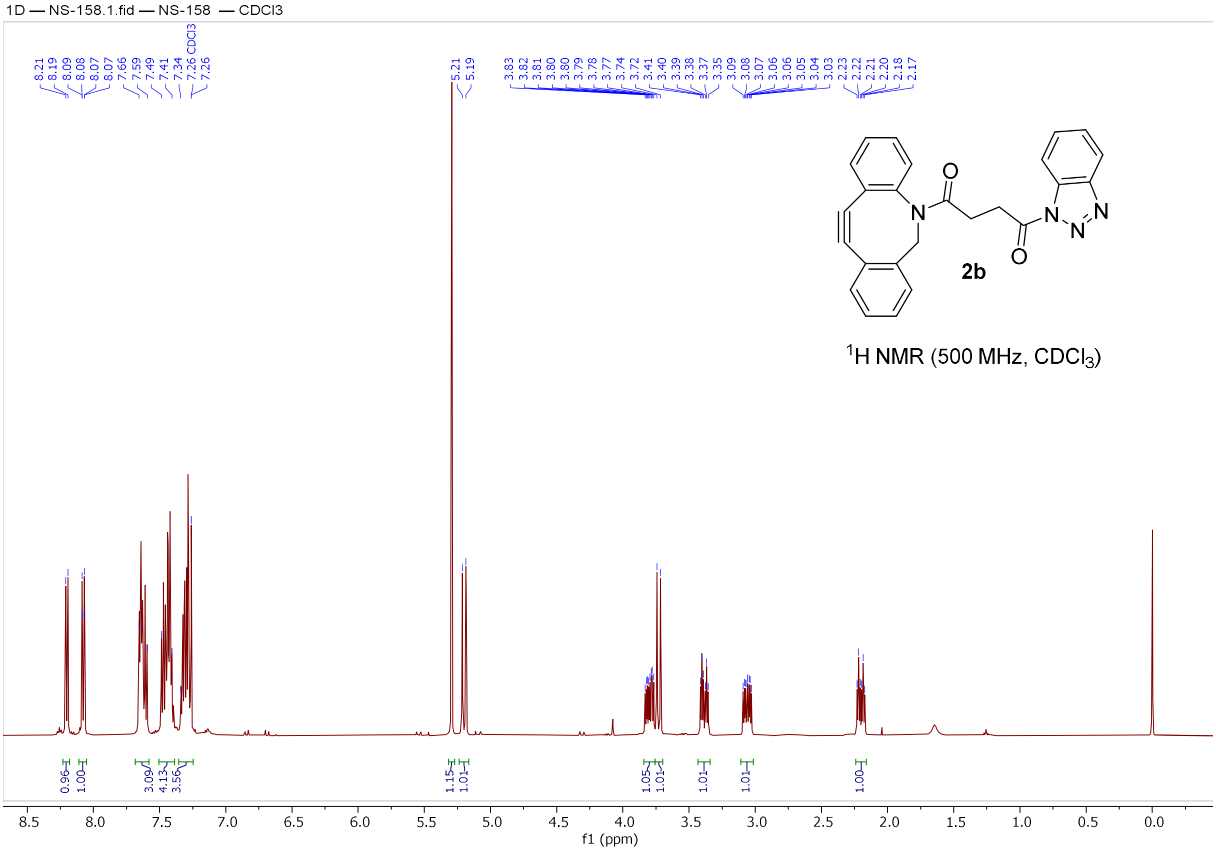
1D — NS-158.1.fid — NS-158 — CDCl3
8.21 8.19 8.09 8.08 8.07 7.66 7.59 7.49 7.41 7.34 7.26 CDCl3 7.26
5.21 5.19
3.83 3.82 3.81 3.80 3.80 3.79 3.78 3.77 3.74 3.72 3.41 3.40 3.39 3.38 3.37 3.35 3.09 3.08 3.07 3.06 3.05 3.04 3.03 2.23 2.22 2.21 2.20 2.18 2.17
2b
^{1}H NMR (500 MHz, $CDCl_3$)
0.96 1.00 3.09 4.13 3.56 1.15 1.01 1.05 1.01 1.01 1.01 1.00
f1 (ppm)

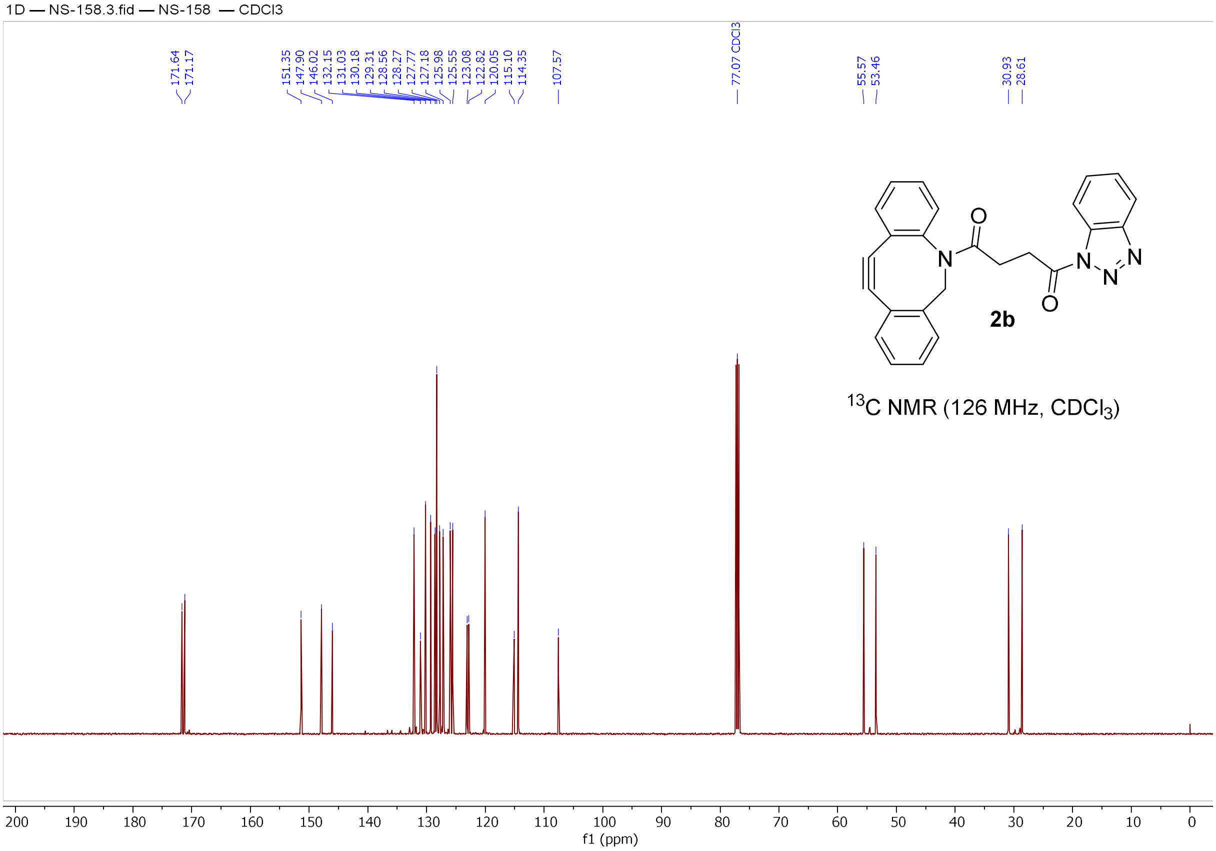
1D — NS-158.3.fid — NS-158 — CDCl3
171.64 171.17
151.35 147.90 146.02 132.15 131.03 130.18 129.31 128.56 128.27 127.77 127.18 125.98 125.55 123.08 122.82 120.05 115.10 114.35 107.57
77.07 CDCl3
55.57 53.46
30.93 28.61
2b
^{13}C NMR (126 MHz, $CDCl_3$)
f1 (ppm)

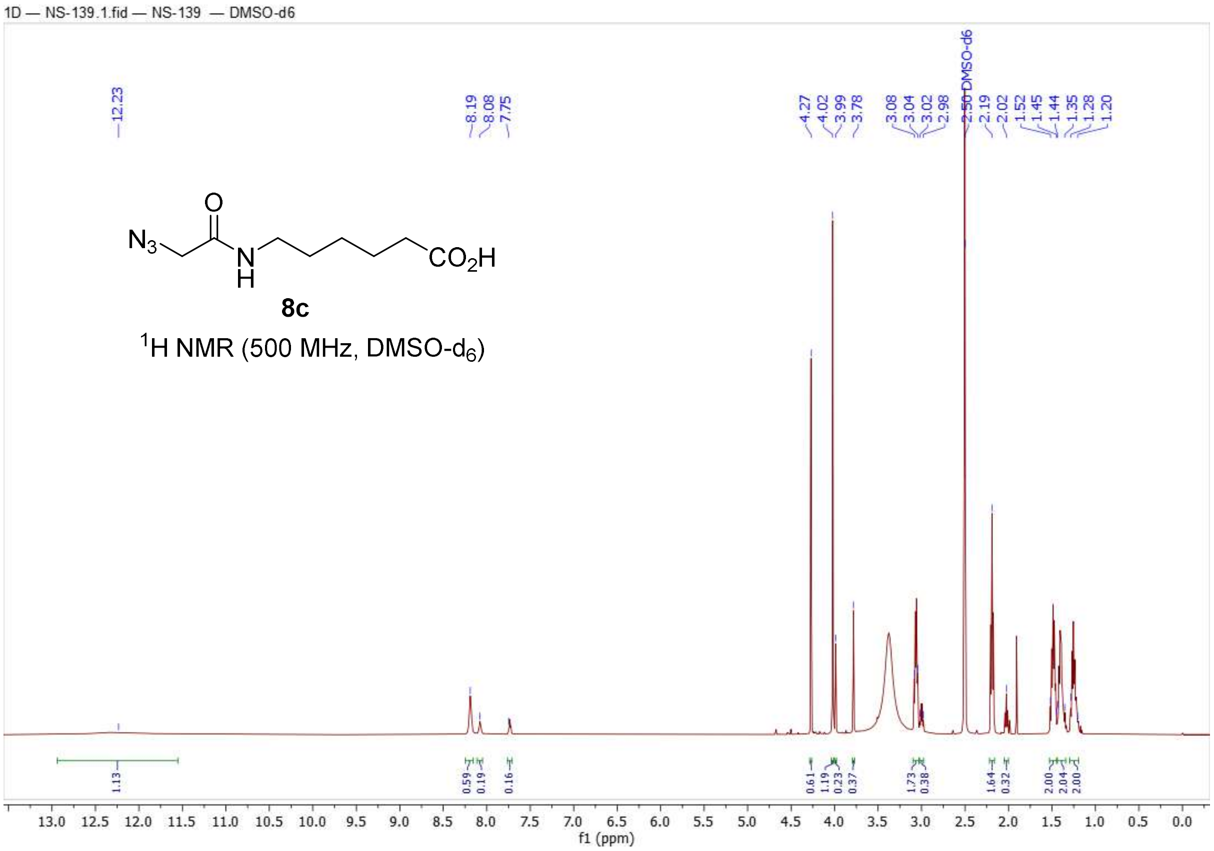
1D — NS-139.1.fid — NS-139 — DMSO-d6
12.23
8.19
8.08
7.75
4.27
4.02
3.99
3.78
3.08
3.04
3.02
2.98
2.50 DMSO-d6
2.19
2.02
1.52
1.45
1.44
1.35
1.28
1.20
N3
O
N
H
CO2H
8c
1H NMR (500 MHz, DMSO-d6)
1.13
0.59
0.19
0.16
0.61
1.19
0.23
0.37
1.73
0.38
1.64
0.32
2.00
2.04
2.00
f1 (ppm)

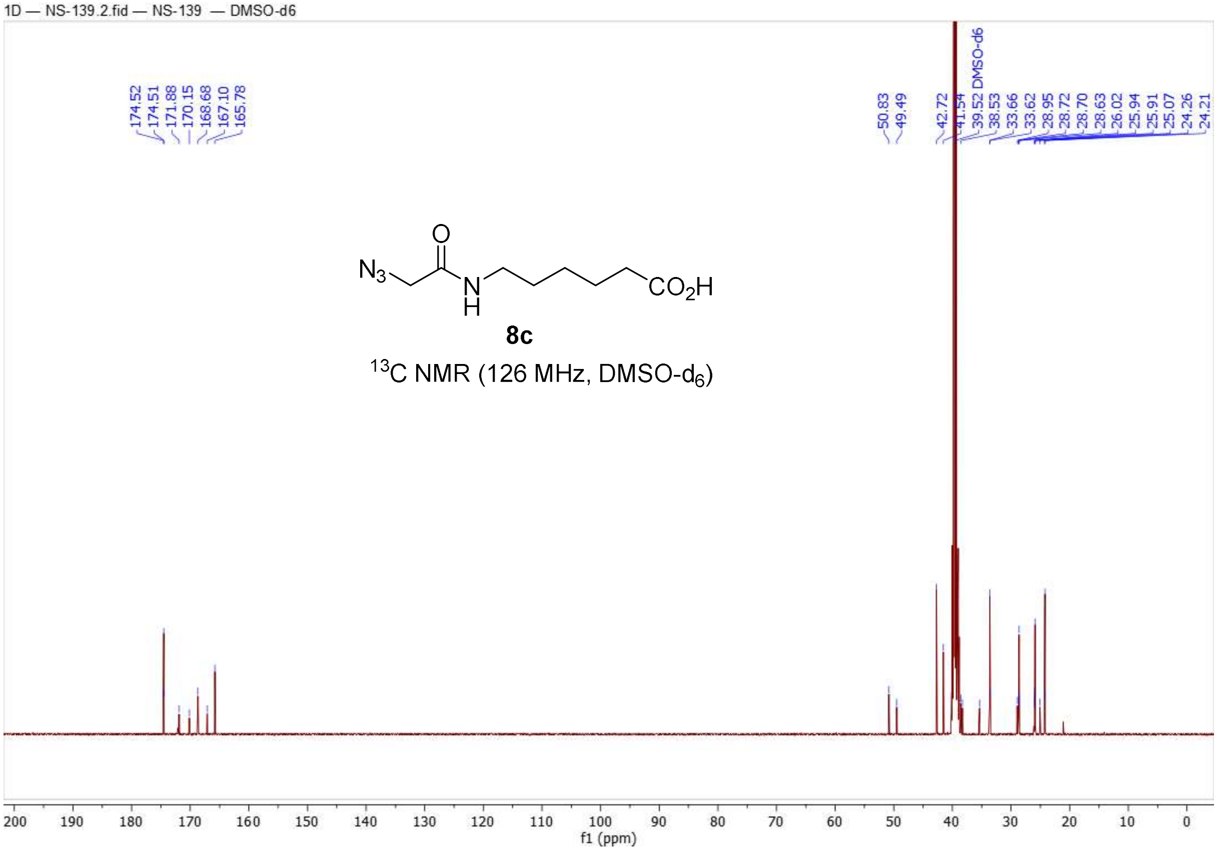
1D — NS-139.2.fid — NS-139 — DMSO-d6
174.52
174.51
171.88
170.15
168.63
167.10
165.78
50.83
49.49
42.72
41.54
39.52 DMSO-d6
38.53
33.66
33.62
28.95
28.72
28.70
28.63
26.02
25.94
25.91
25.07
24.26
24.21
N3
O
N
H
CO2H
8c
13C NMR (126 MHz, DMSO-d6)
f1 (ppm)

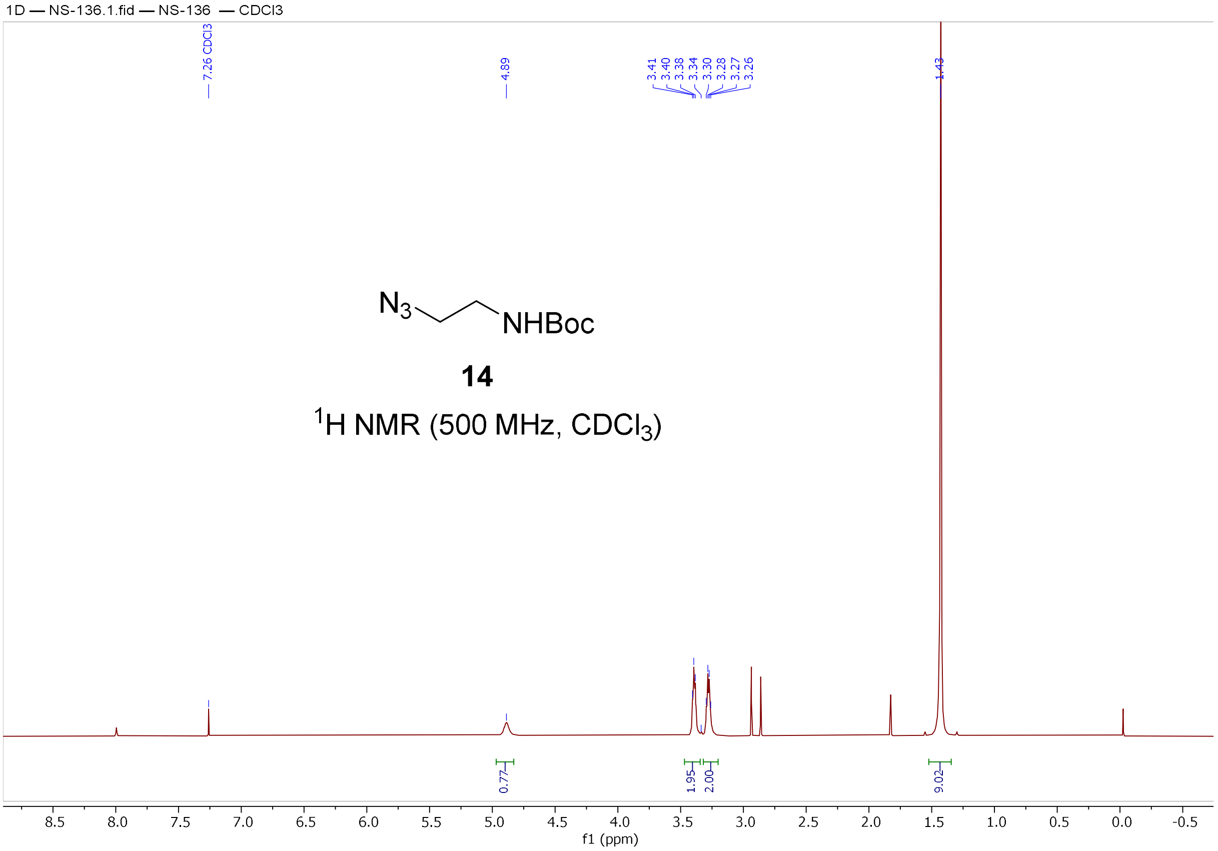
1D — NS-136.1.fid — NS-136 — CDCl3
7.26 CDCl3
4.89
3.41
3.40
3.38
3.34
3.30
3.28
3.27
3.26
1.43
N3
NHBoc
14
1H NMR (500 MHz, CDCl3)
0.77
1.95
2.00
9.02
f1 (ppm)

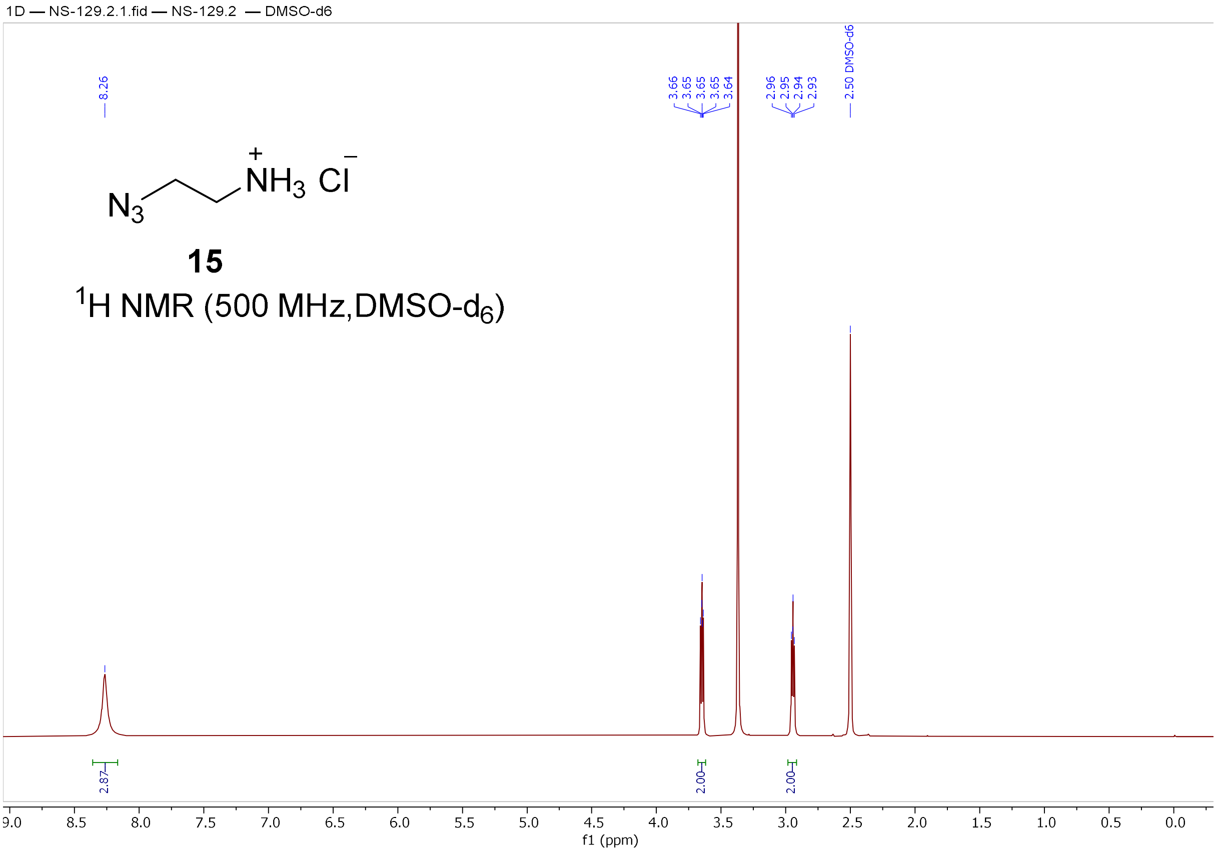
1D — NS-129.2.1.fid — NS-129.2 — DMSO-d6
8.26
3.66
3.65
3.65
3.65
3.64
2.96
2.95
2.94
2.93
2.50 DMSO-d6
N3
NH3 Cl
15
1H NMR (500 MHz,DMSO-d6)
2.87
2.00
2.00
f1 (ppm)

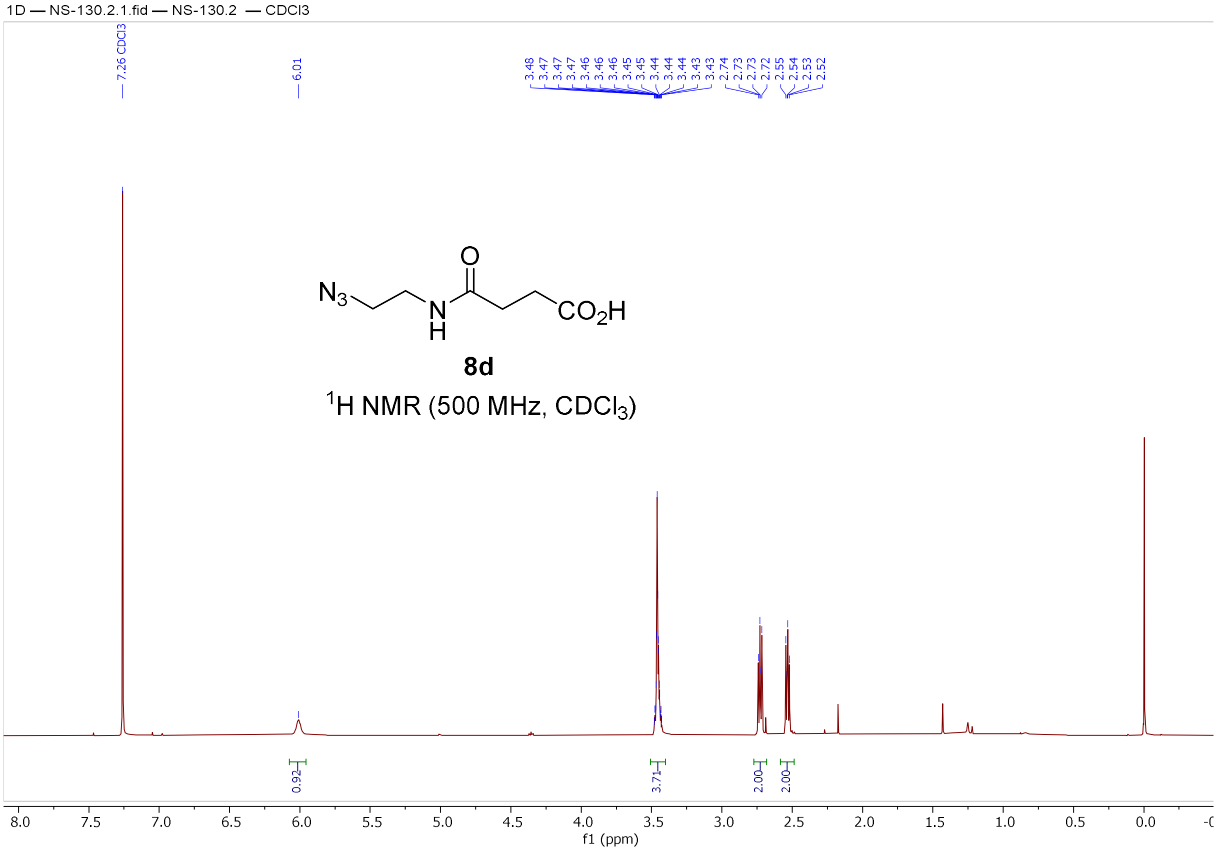
1D — NS-130.2.1.fid — NS-130.2 — CDCl3
7.26 CDCl3
6.01
3.48 3.47 3.47 3.47 3.46 3.46 3.46 3.45 3.45 3.44 3.44 3.44 3.43 3.43 2.74 2.73 2.73 2.72 2.55 2.54 2.53 2.52
8d
1H NMR (500 MHz, CDCl3)
0.92 3.71 2.00 2.00
f1 (ppm)

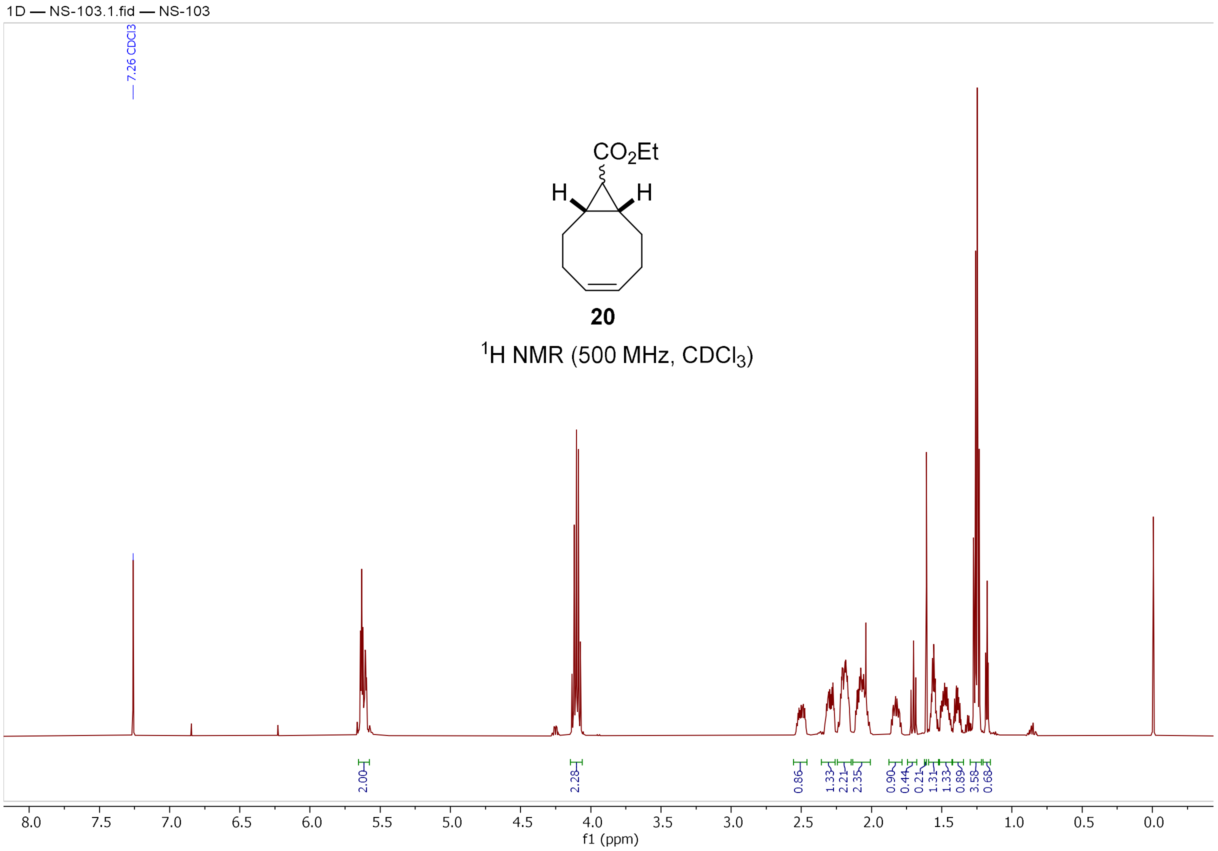
1D — NS-103.1.fid — NS-103
7.26 CDCl3
CO2Et
H
H
20
1H NMR (500 MHz, CDCl3)
2.00 2.28 0.86 1.33 2.21 2.35 0.90 0.44 0.21 1.31 1.33 0.89 3.58 0.68
f1 (ppm)

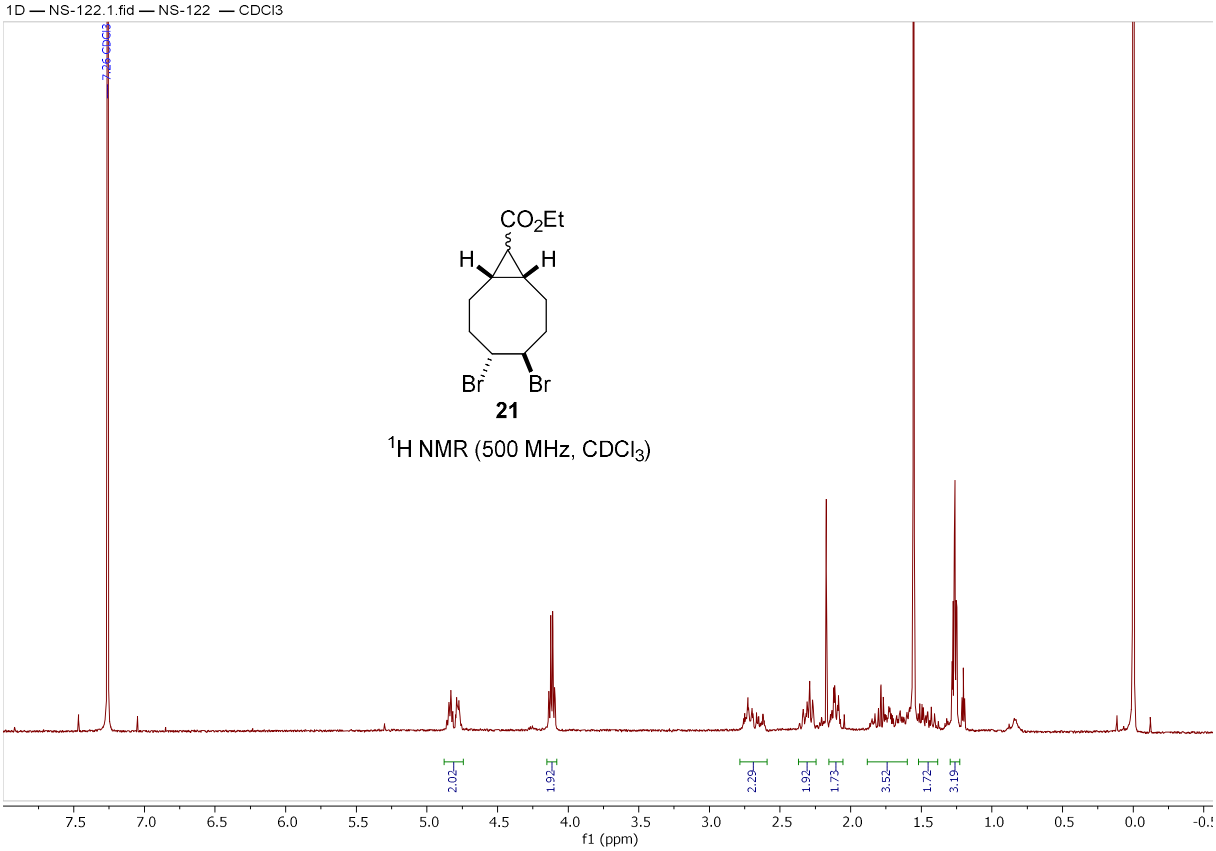
1D — NS-122.1.fid — NS-122 — CDCl3
7.26 CDCl3
CO2Et
H
H
Br
Br
21
1H NMR (500 MHz, CDCl3)
2.02
1.92
2.29
1.92
1.73
3.52
1.72
3.19
7.5 7.0 6.5 6.0 5.5 5.0 4.5 4.0 3.5 3.0 2.5 2.0 1.5 1.0 0.5 0.0
f1 (ppm)

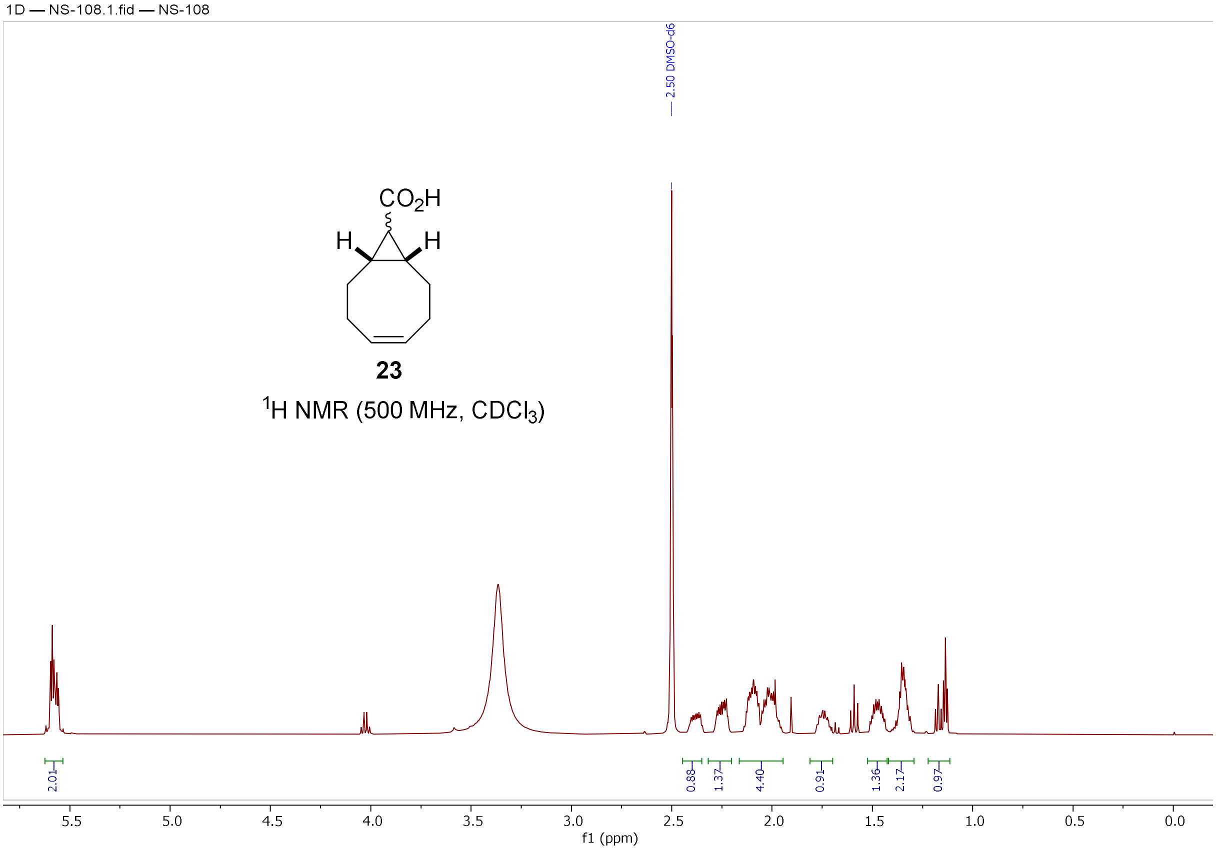
1D — NS-108.1.fid — NS-108
2.50 DMSO-d6
CO2H
H
H
23
1H NMR (500 MHz, CDCl3)
2.01
0.88
1.37
4.40
0.91
1.36
2.17
0.97
5.5 5.0 4.5 4.0 3.5 3.0 2.5 2.0 1.5 1.0 0.5 0.0
f1 (ppm)

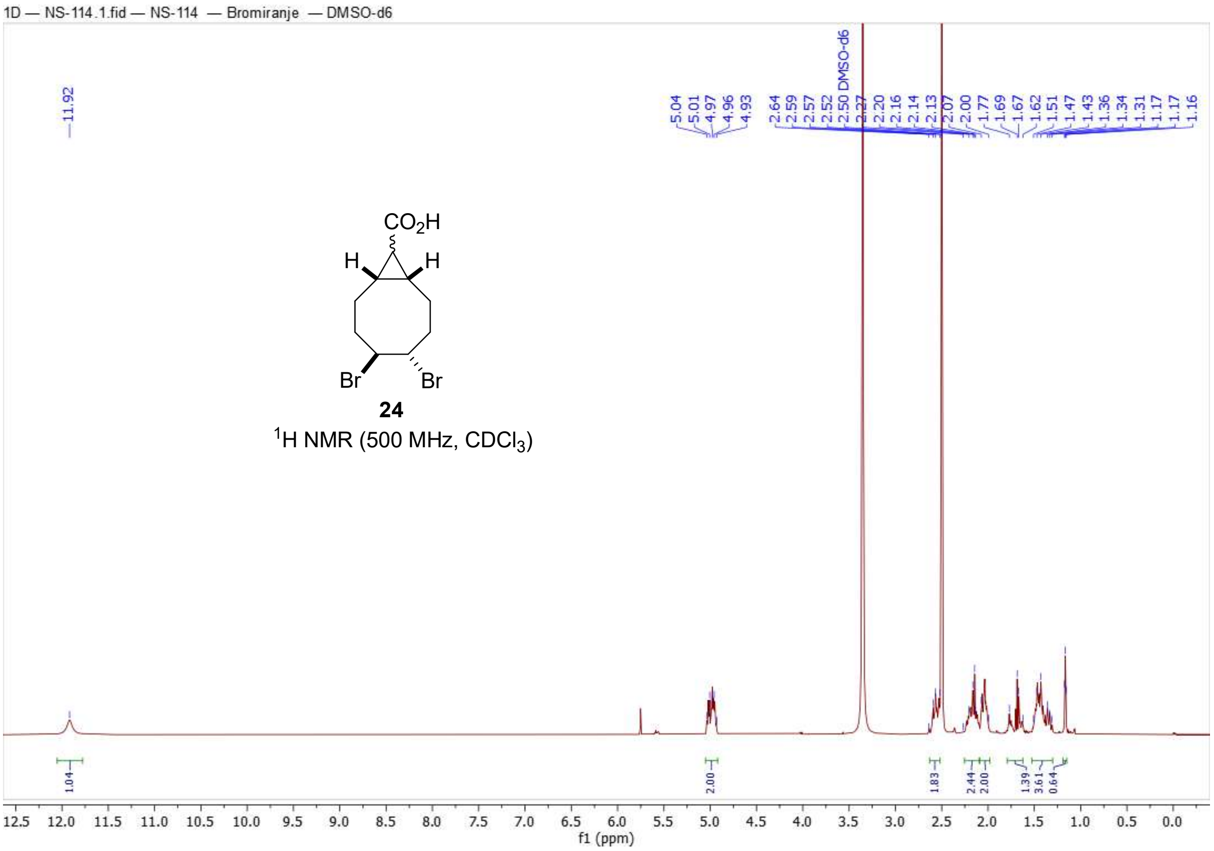
1D — NS-114.1.fid — NS-114 — Bromiranje — DMSO-d6
CO2H
H
H
Br
Br
24
1H NMR (500 MHz, CDCl3)
f1 (ppm)

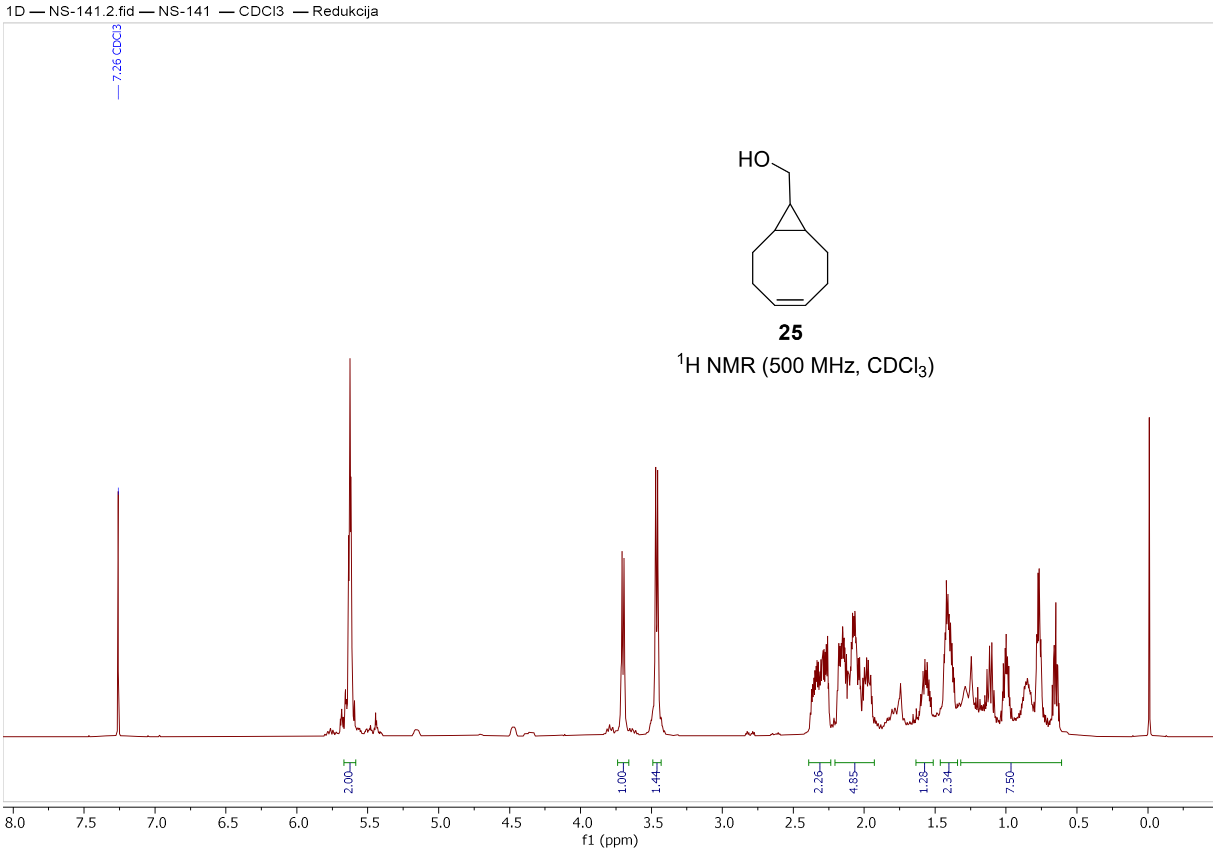
1D — NS-141.2.fid — NS-141 — CDCl3 — Redukcija
HO
25
1H NMR (500 MHz, CDCl3)
f1 (ppm)

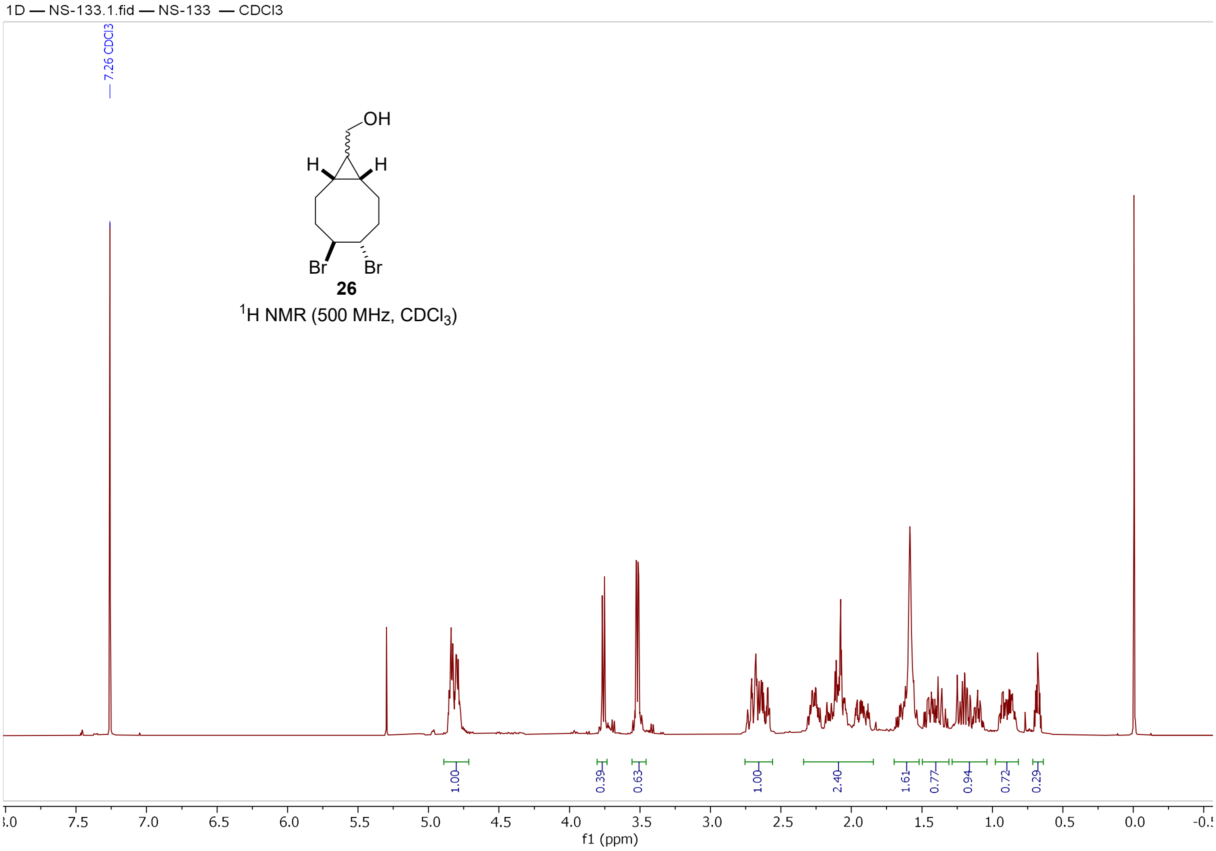
1D — NS-133.1.fid — NS-133 — CDCl3
7.26 CDCl3
OH
H
H
Br
Br
26
1H NMR (500 MHz, CDCl3)
1.00
0.39
0.63
1.00
2.40
1.61
0.77
0.94
0.72
0.29
f1 (ppm)

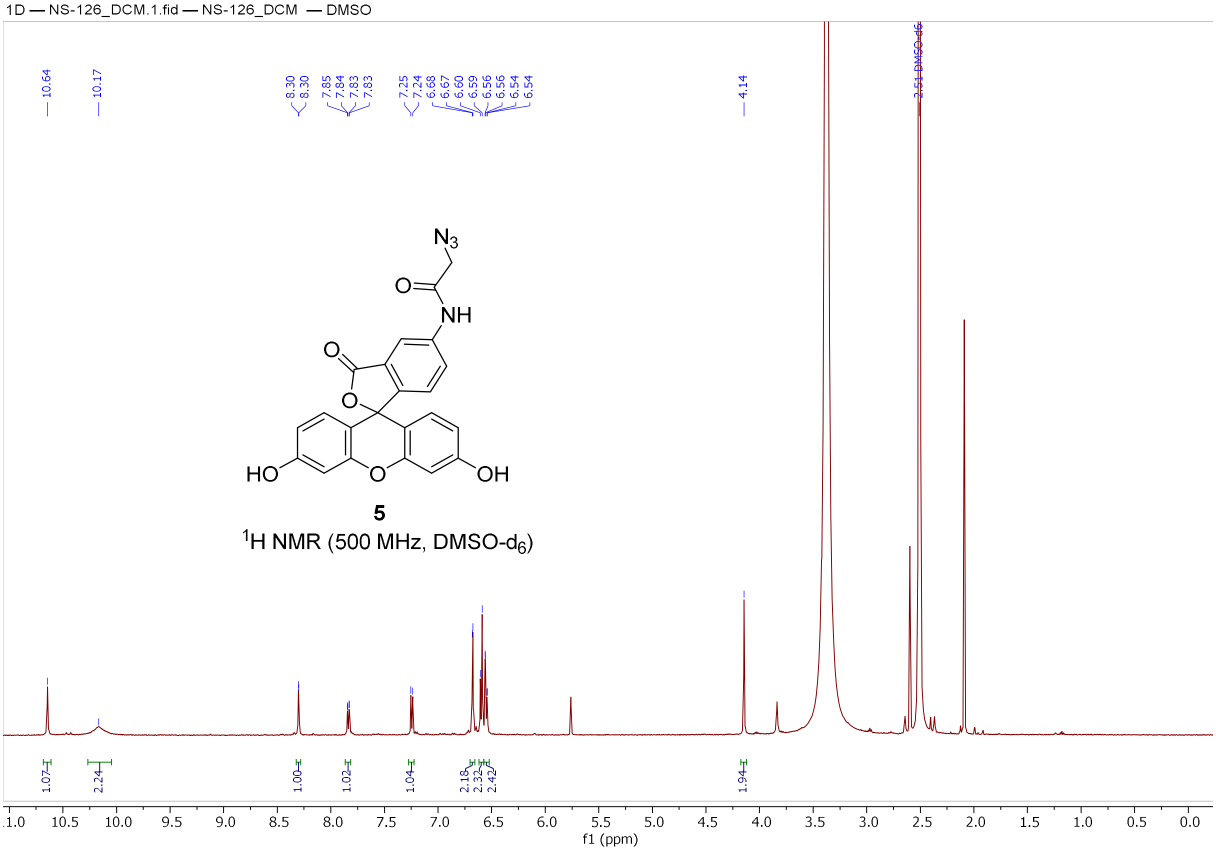
1D — NS-126_DCM.1.fid — NS-126_DCM — DMSO
10.64
10.17
8.30
8.30
7.85
7.84
7.83
7.83
7.25
7.24
6.68
6.67
6.60
6.59
6.56
6.56
6.54
6.54
4.14
2.51 DMSO-d6
N3
O
NH
O
O
HO
O
OH
5
1H NMR (500 MHz, DMSO-d6)
1.07
2.24
1.00
1.02
1.04
2.18
2.32
2.42
1.94
f1 (ppm)

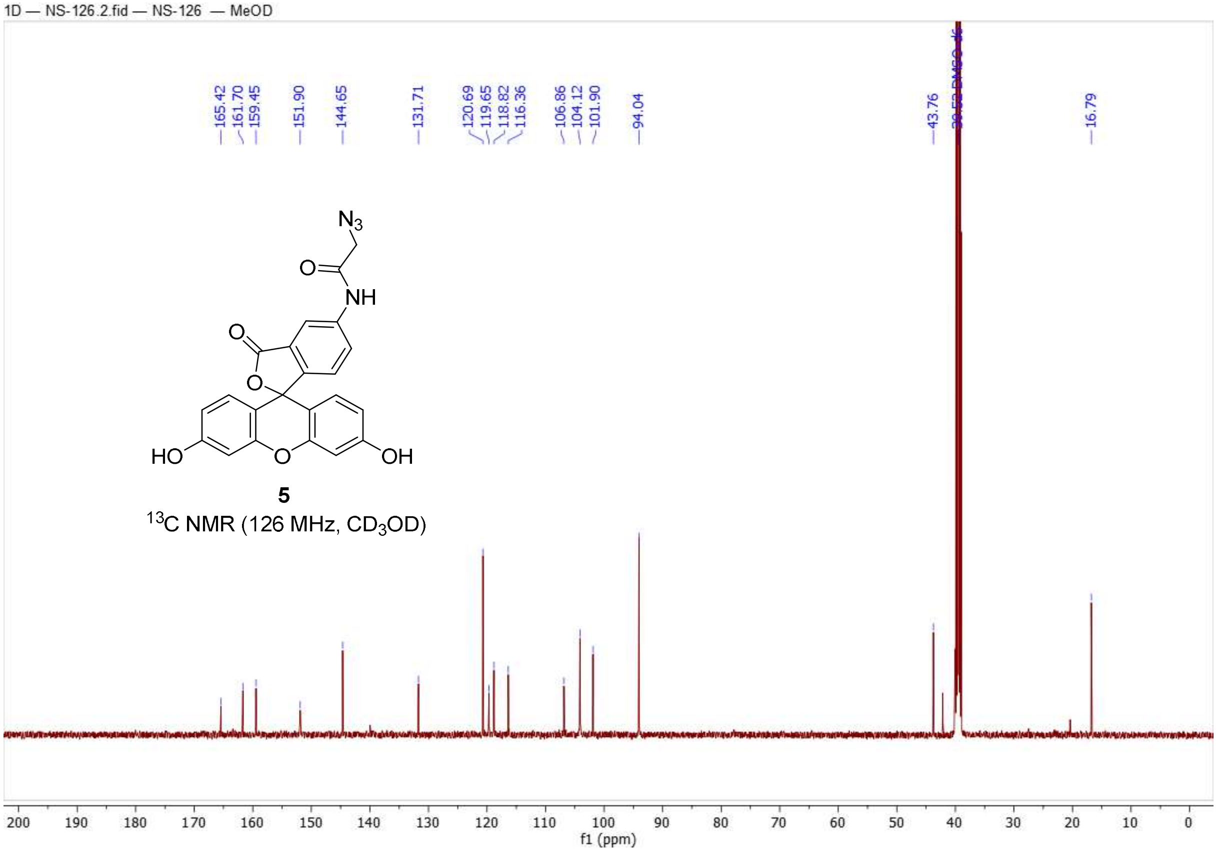
1D — NS-126.2.fid — NS-126 — MeOD
165.42
161.70
159.45
151.90
144.65
131.71
120.69
119.65
118.82
116.36
106.86
104.12
101.90
94.04
43.76
16.79
N3
O
NH
O
O
HO
O
OH
5
13C NMR (126 MHz, CD3OD)
f1 (ppm)

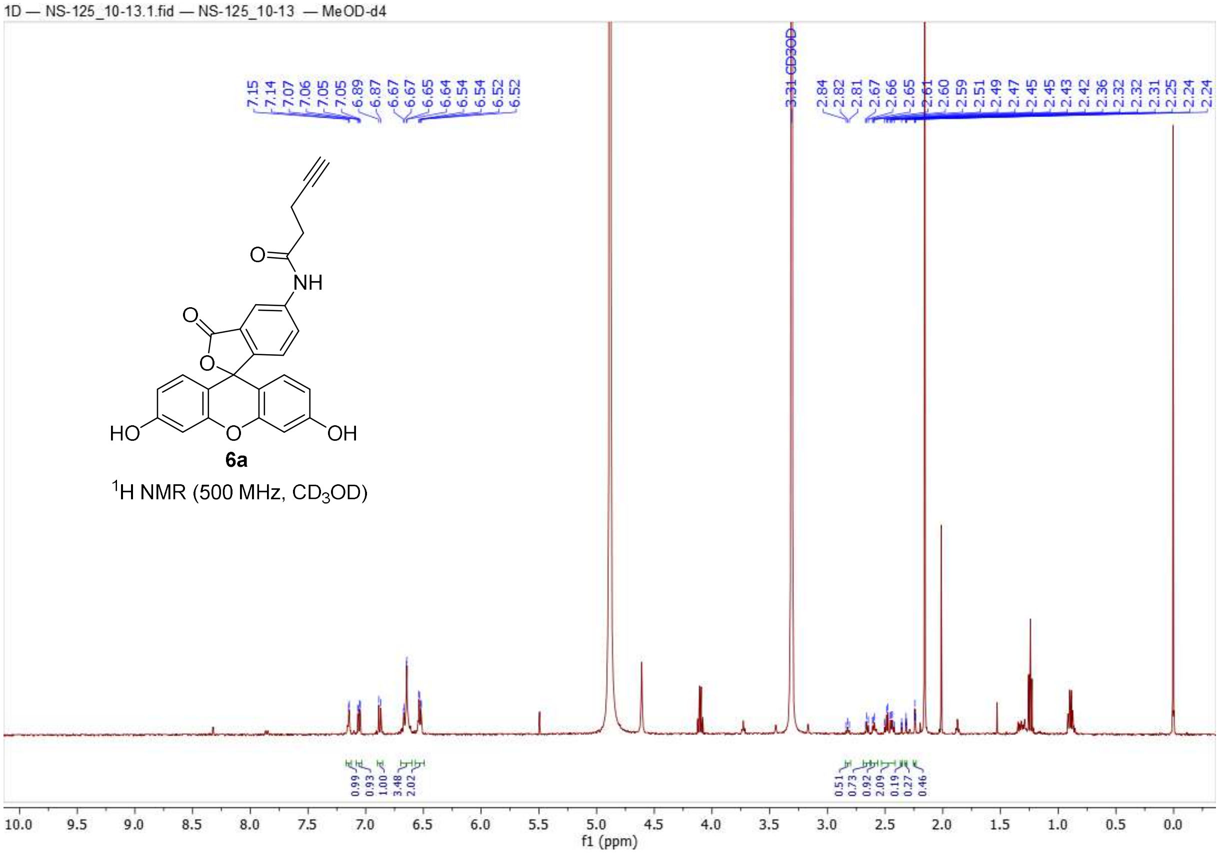
1D — NS-125_10-13.1.fid — NS-125_10-13 — MeOD-d4
7.15
7.14
7.07
7.06
7.05
7.05
6.89
6.87
6.67
6.67
6.65
6.64
6.54
6.52
6.52
3.31 CD3OD
2.84
2.82
2.81
2.67
2.66
2.65
2.61
2.60
2.59
2.51
2.49
2.47
2.45
2.45
2.43
2.42
2.36
2.32
2.32
2.31
2.25
2.24
2.24
O
NH
O
O
HO
O
OH
6a
1H NMR (500 MHz, CD3OD)
f1 (ppm)

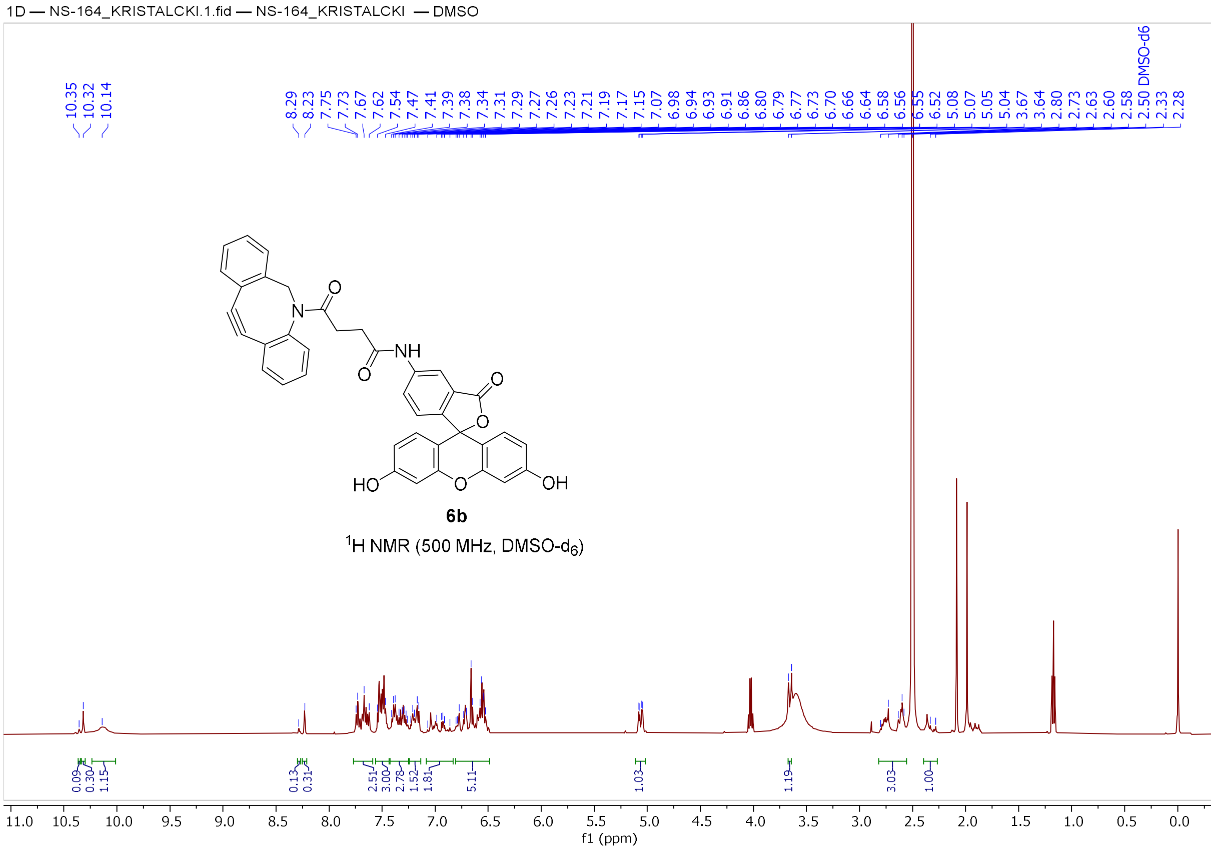
1D — NS-164_KRISTALCKI.1.fid — NS-164_KRISTALCKI — DMSO
6b
1H NMR (500 MHz, DMSO-d6)
f1 (ppm)

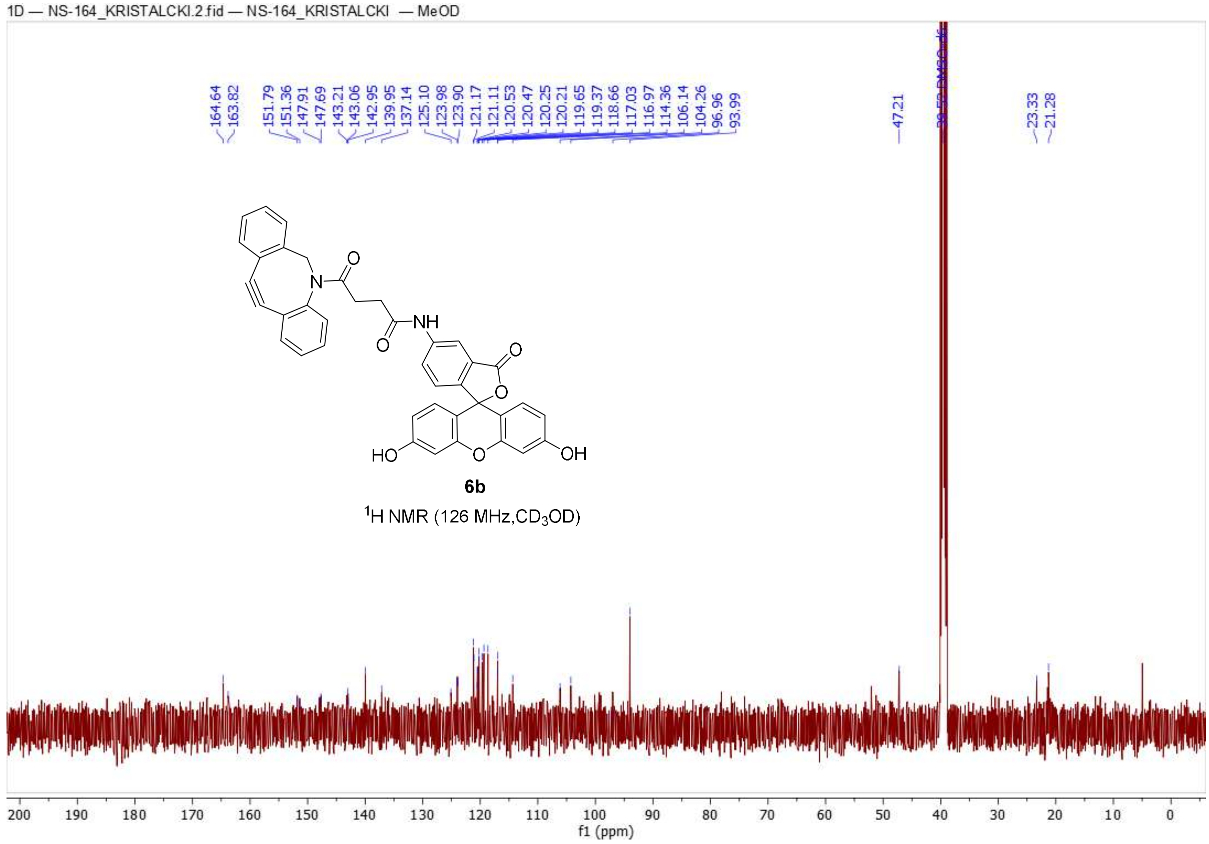
1D — NS-164_KRISTALCKI.2.fid — NS-164_KRISTALCKI — MeOD
6b
1H NMR (126 MHz,CD3OD)
f1 (ppm)

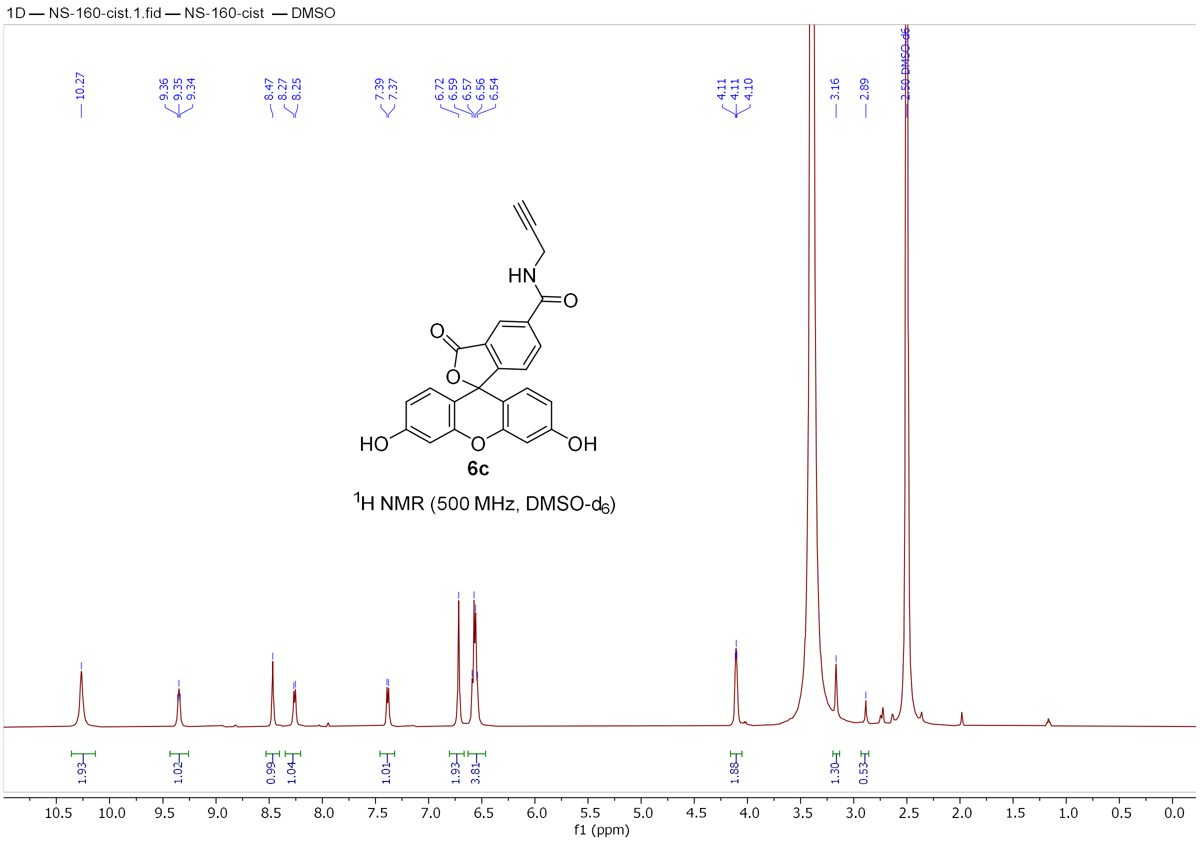
1D — NS-160-cist.1.fid — NS-160-cist — DMSO
10.27
9.36
9.35
9.34
8.47
8.27
8.25
7.39
7.37
6.72
6.59
6.57
6.56
6.54
4.11
4.11
4.10
3.16
2.89
2.50 DMSO-d6
HN
O
O
O
HO
O
OH
6c
1H NMR (500 MHz, DMSO-d6)
1.93
1.02
0.99
1.04
1.01
1.93
3.81
1.88
1.30
0.53
10.5 10.0 9.5 9.0 8.5 8.0 7.5 7.0 6.5 6.0 5.5 5.0 4.5 4.0 3.5 3.0 2.5 2.0 1.5 1.0 0.5 0.0
f1 (ppm)

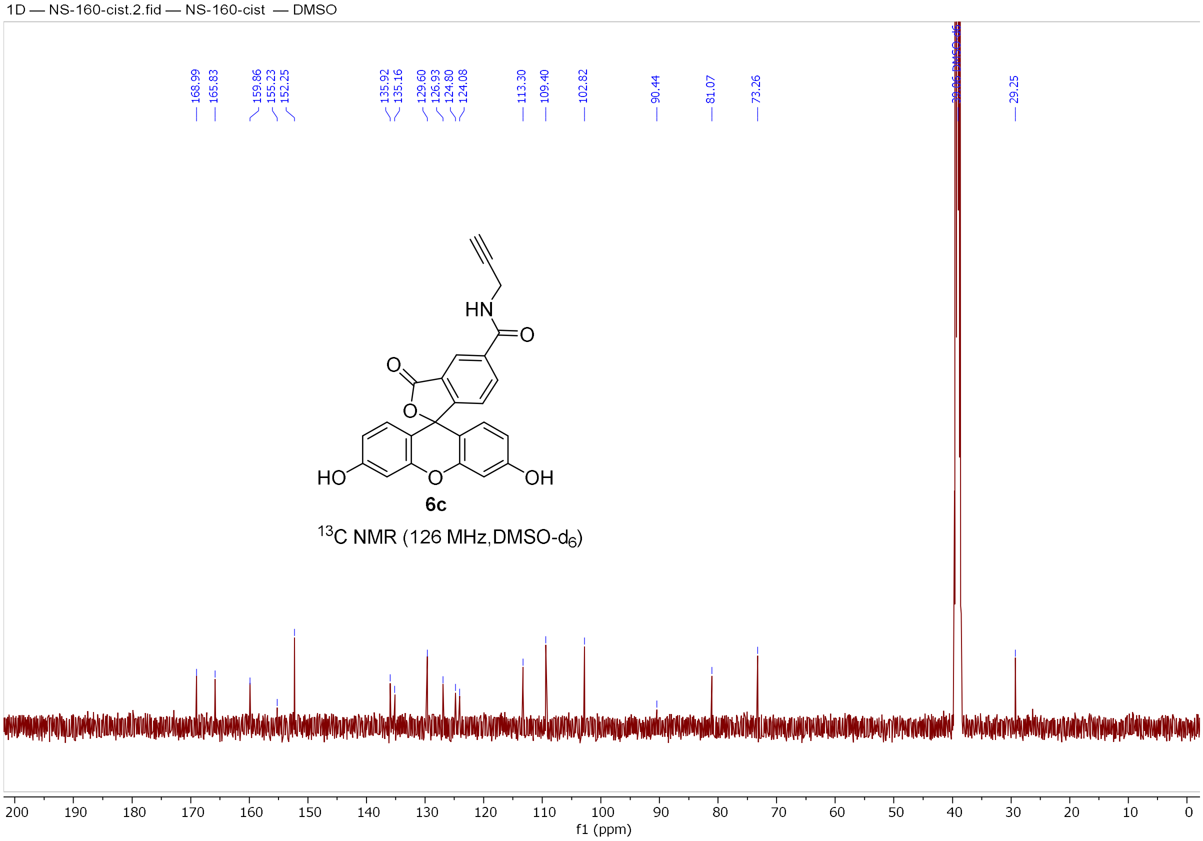
1D — NS-160-cist.2.fid — NS-160-cist — DMSO
168.99
165.83
159.86
155.23
152.25
135.92
135.16
129.60
126.93
124.80
124.08
113.30
109.40
102.82
90.44
81.07
73.26
29.25
HN
O
O
O
HO
O
OH
6c
13C NMR (126 MHz,DMSO-d6)
200 190 180 170 160 150 140 130 120 110 100 90 80 70 60 50 40 30 20 10 0
f1 (ppm)

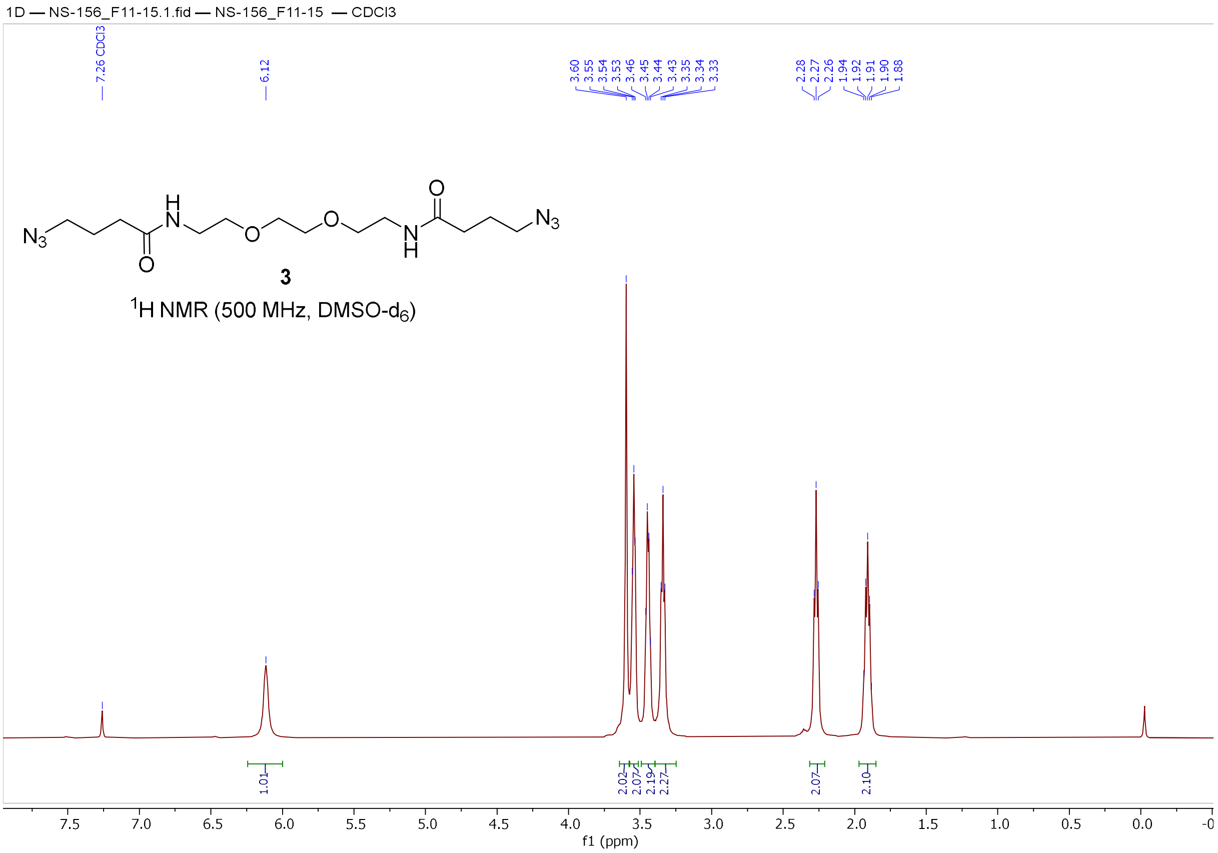
1D — NS-156_F11-15.1.fid — NS-156_F11-15 — CDCl3
3
^{1}H NMR (500 MHz, DMSO-d$_6$)
f1 (ppm)

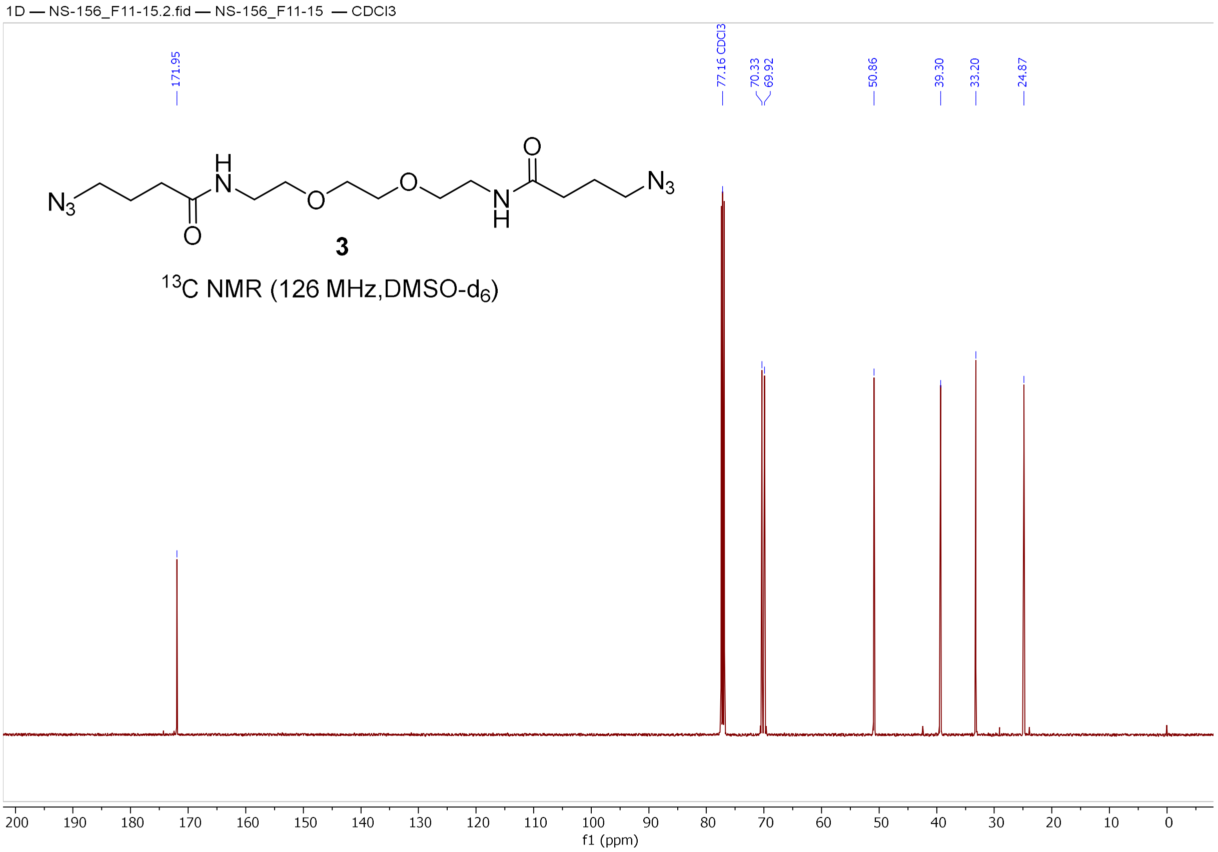
1D — NS-156_F11-15.2.fid — NS-156_F11-15 — CDCl3
3
^{13}C NMR (126 MHz,DMSO-d$_6$)
f1 (ppm)

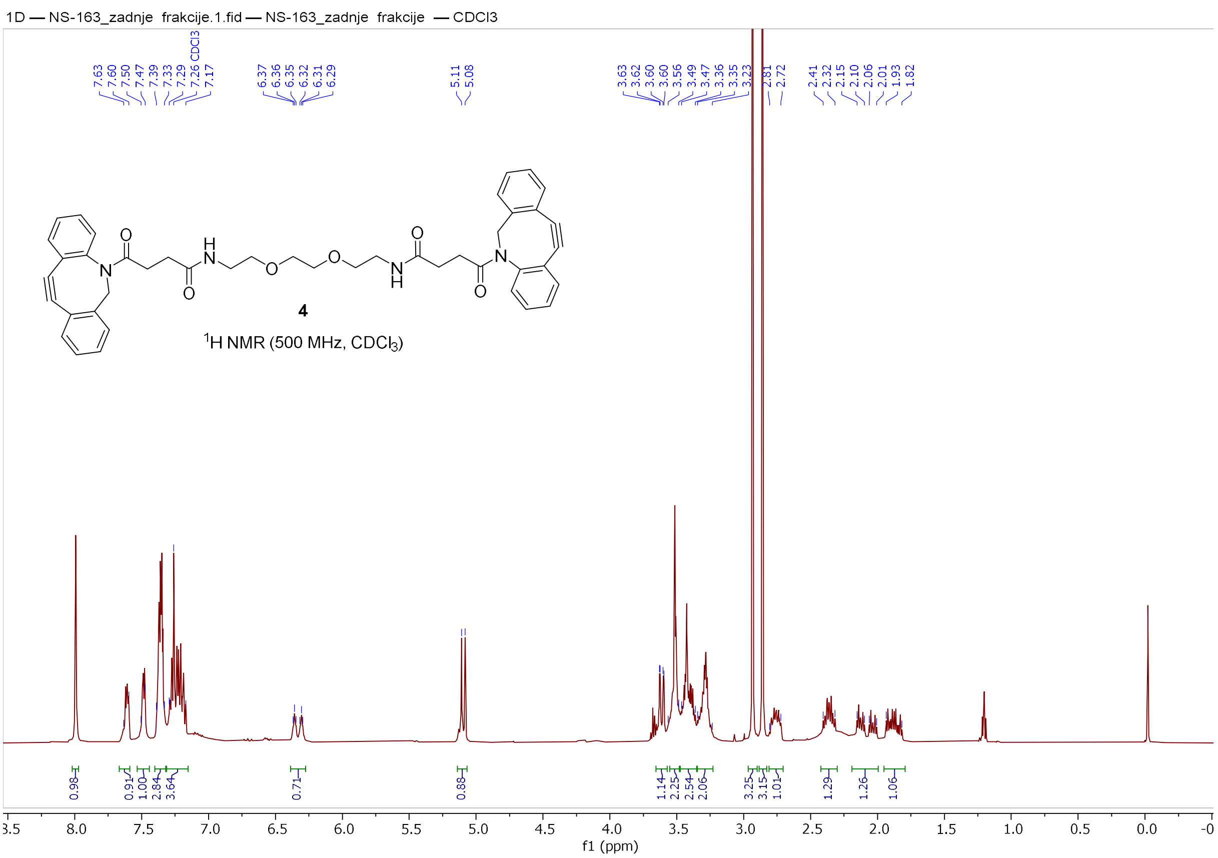
1D — NS-163_zadnje frakcije.1.fid — NS-163_zadnje frakcije — CDCl3
7.63 7.60 7.50 7.47 7.39 7.33 7.29 7.26 CDCl3 7.17
6.37 6.36 6.35 6.32 6.31 6.29
5.11 5.08
3.63 3.62 3.60 3.60 3.56 3.49 3.47 3.36 3.36 3.35 3.23 2.81 2.72
2.41 2.32 2.15 2.10 2.06 2.01 1.93 1.82
4
^{1}H NMR (500 MHz, CDCl$_3$)
0.98 0.91 1.00 2.84 3.64 0.71 0.88 1.14 2.25 2.54 2.06 3.25 3.15 1.01 1.29 1.26 1.06
f1 (ppm)

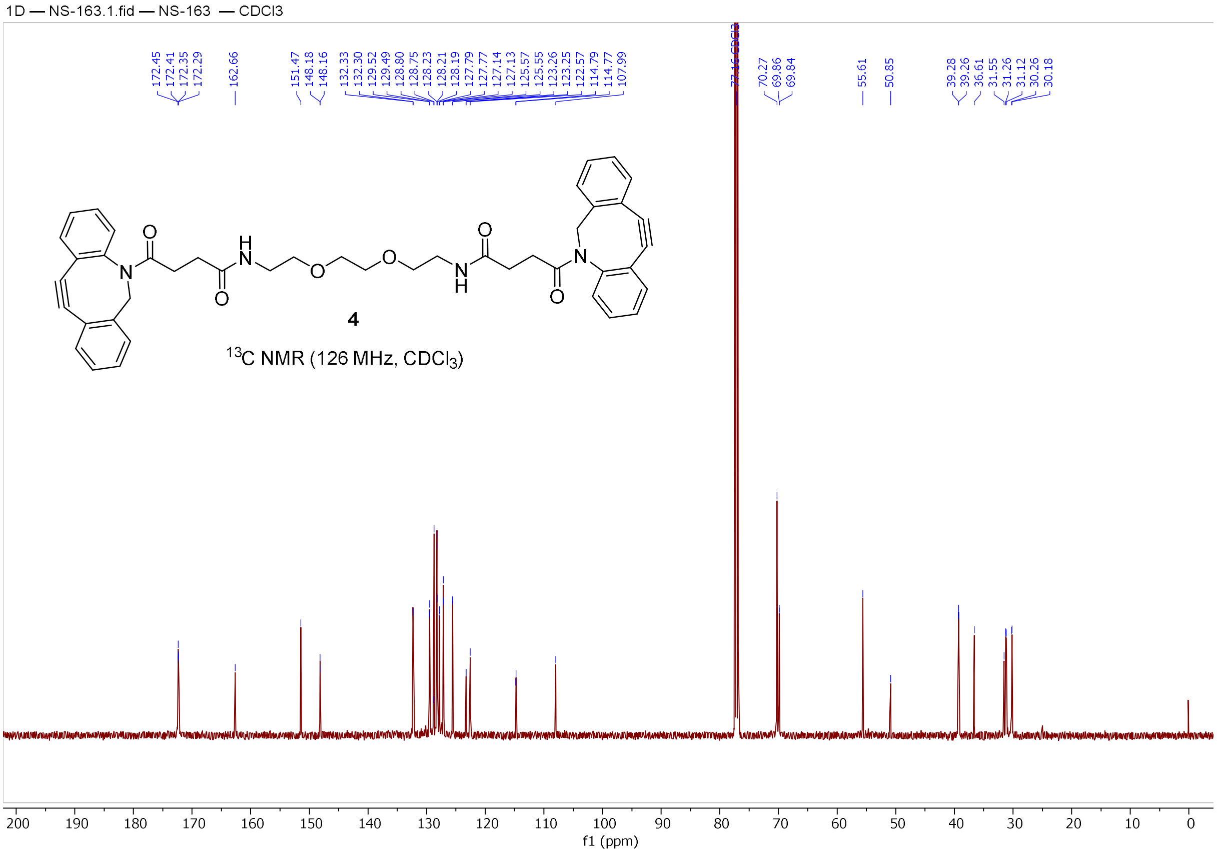
1D — NS-163.1.fid — NS-163 — CDCl3
172.45 172.41 172.35 172.29 162.66
151.47 148.18 148.16
132.33 132.30 129.52 129.49 128.80 128.75 128.23 128.21 128.19 127.79 127.77 127.14 127.13 125.57 125.55 123.26 123.25 122.57 114.79 114.77 107.99
77.16 CDCl3 70.27 69.86 69.84
55.61 50.85
39.28 39.26 36.61 31.55 31.26 31.12 30.26 30.18
4
^{13}C NMR (126 MHz, CDCl$_3$)
f1 (ppm)

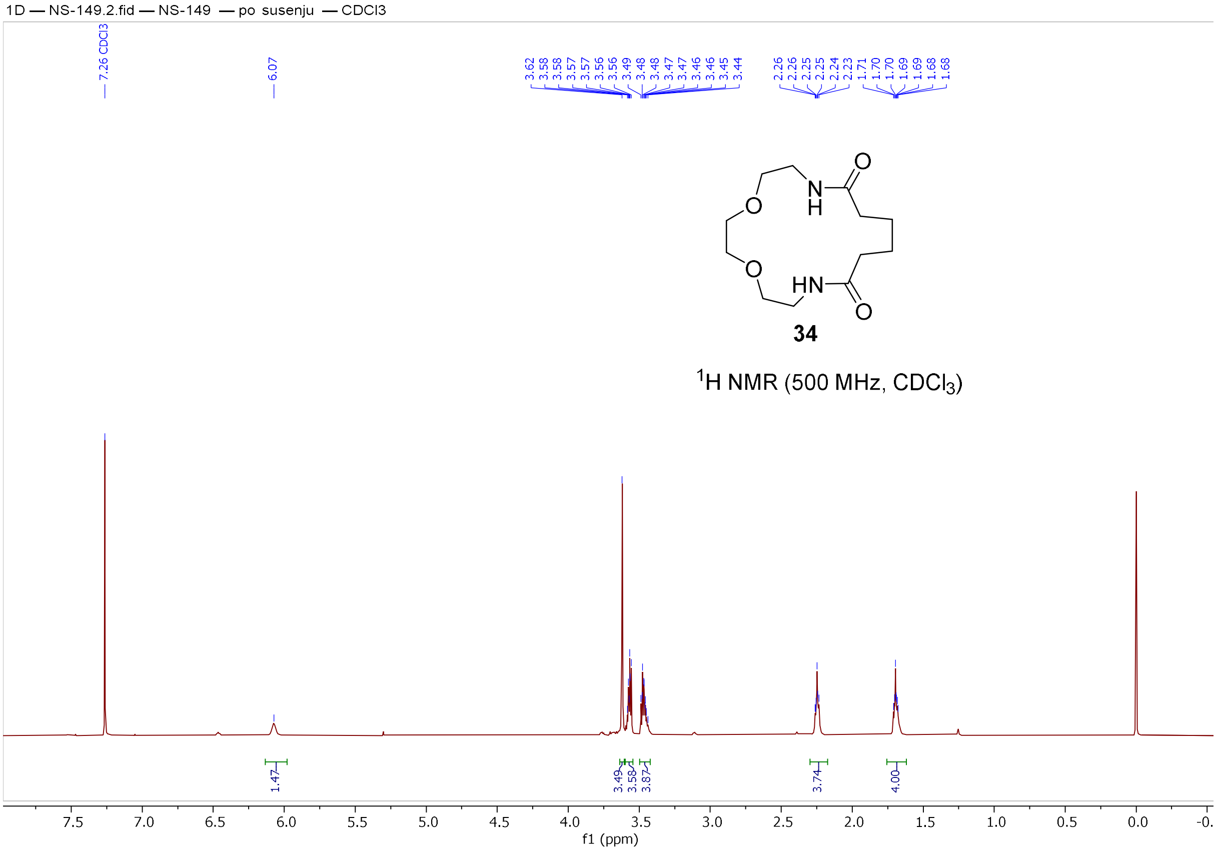
1D — NS-149.2.fid — NS-149 — po susenju — CDCl3
34
^{1}H NMR (500 MHz, CDCl$_3$)
f1 (ppm)

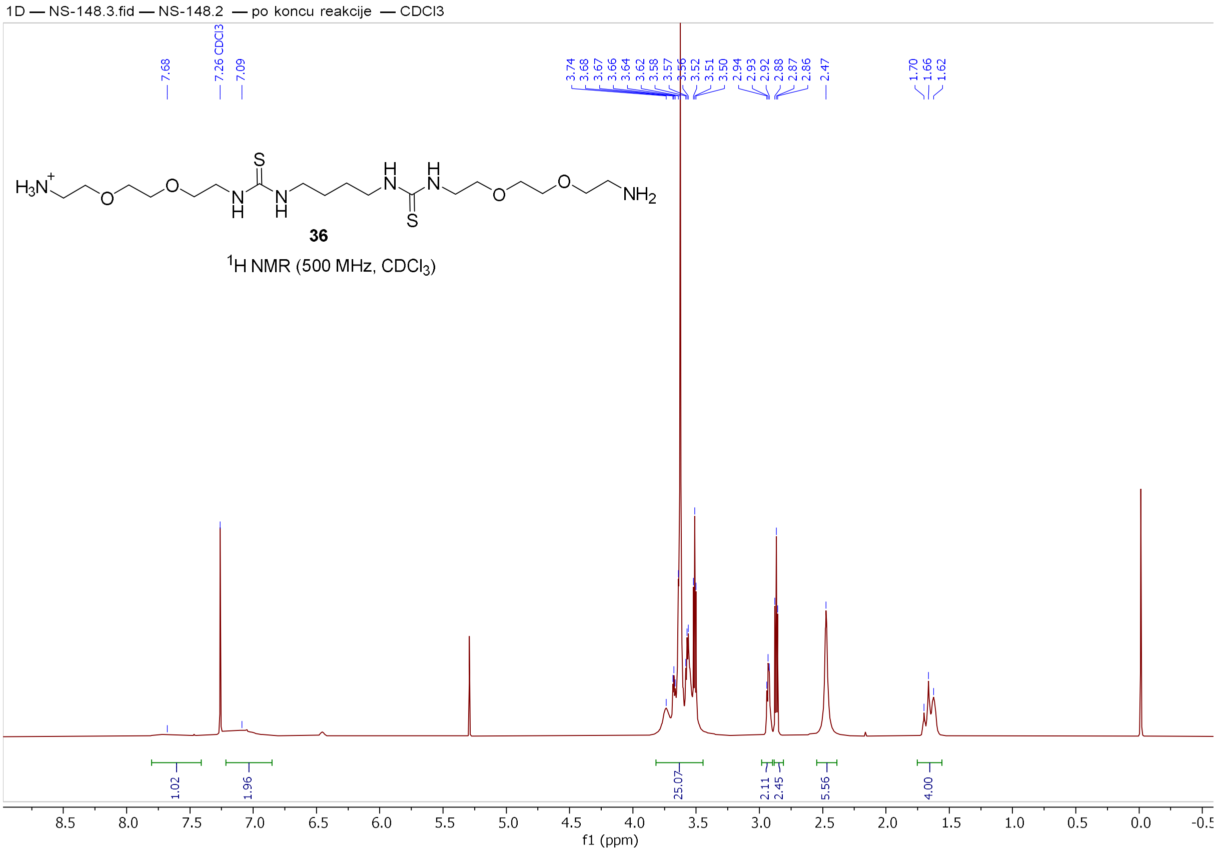
1D — NS-148.3.fid — NS-148.2 — po koncu reakcije — CDCl3
36
^{1}H NMR (500 MHz, CDCl$_3$)
f1 (ppm)

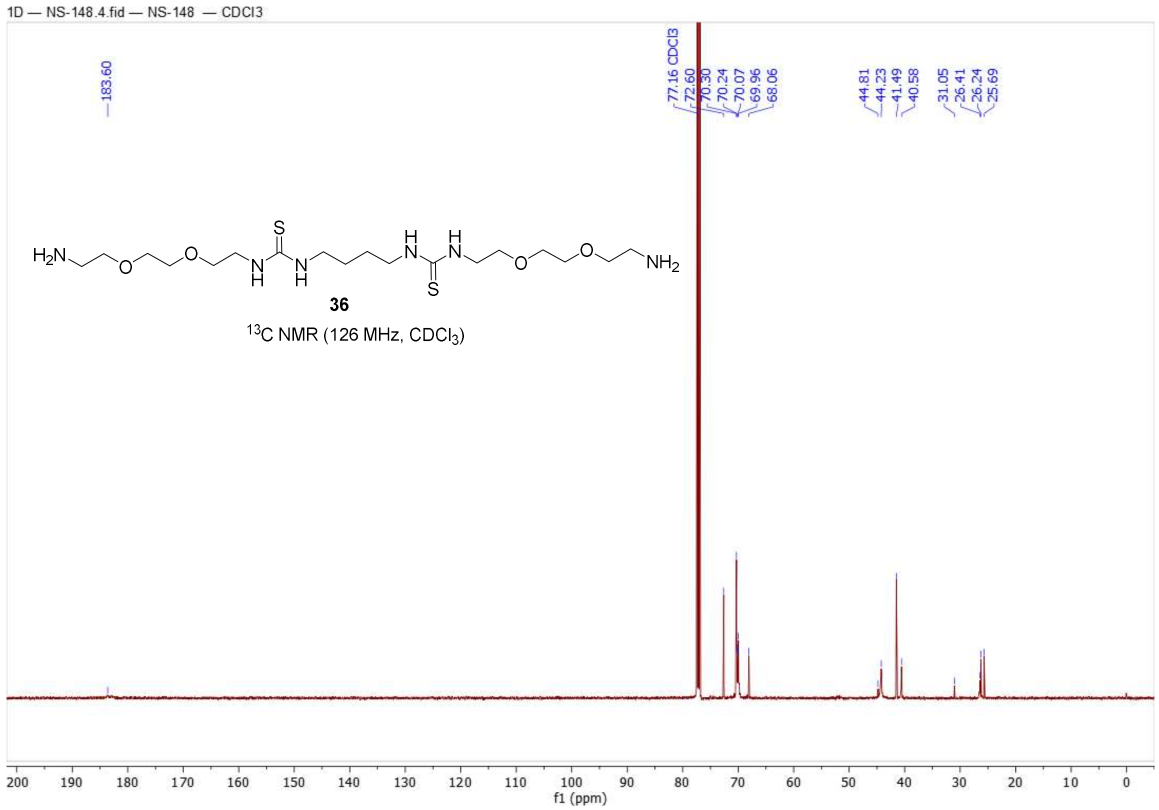
1D — NS-148.4.fid — NS-148 — CDCl3
183.60
77.16 CDCl3
72.60
70.30
70.24
70.07
69.96
68.06
44.81
44.23
41.49
40.58
31.05
26.41
26.24
25.69
36
13C NMR (126 MHz, CDCl3)
f1 (ppm)
